# Immunoliposome-based targeted delivery of the CRISPR/Cas9gRNA-IL30 complex inhibits prostate cancer and prolongs survival

**DOI:** 10.1038/s12276-024-01310-2

**Published:** 2024-09-04

**Authors:** Cristiano Fieni, Carlo Sorrentino, Stefania Livia Ciummo, Antonella Fontana, Lavinia Vittoria Lotti, Sofia Scialis, Darien Calvo Garcia, Massimo Caulo, Emma Di Carlo

**Affiliations:** 1 Department of Medicine and Sciences of Aging, “G. d’Annunzio” University of Chieti-Pescara, 66100 Chieti, Italy; 2 Anatomic Pathology and Immuno-Oncology Unit, Center for Advanced Studies and Technology (CAST), “G. d’Annunzio” University of Chieti-Pescara, 66100 Chieti, Italy; 3 Department of Pharmacy, “G. d’Annunzio” University of Chieti-Pescara, 66100 Chieti, Italy; 4 UDA-TECHLAB Research Center, “G. d’Annunzio” University of Chieti-Pescara, 66100 Chieti, Italy; 5 Department of Experimental Medicine, La Sapienza University of Rome, 00161 Rome, Italy; 6 Department of Neuroscience, Imaging and Clinical Sciences, “G. d’Annunzio” University of Chieti-Pescara, 66100 Chieti, Italy; 7 Institute for Advanced Biomedical Technologies (ITAB), “G. d’Annunzio” University of Chieti-Pescara, 66100 Chieti, Italy

**Keywords:** Prostate cancer, Cancer microenvironment

## Abstract

The development of selective and nontoxic immunotherapy targeting prostate cancer (PC) is challenging. Interleukin (IL)30 plays immunoinhibitory and oncogenic roles in PC, and its tumor-specific suppression may have significant clinical implications. CRISPR/Cas9-mediated IL30 gene deletion in PC xenografts using anti-PSCA antibody-driven lipid nanocomplexes (Cas9gRNA-hIL30-PSCA NxPs) revealed significant genome editing efficiency and circulation stability without off-target effects or organ toxicity. Biweekly intravenous administration of Cas9gRNA-hIL30-PSCA NxPs to PC-bearing mice inhibited tumor growth and metastasis and improved survival. Mechanistically, Cas9gRNA-hIL30-PSCA NxPs suppressed ANGPTL 1/2/4, IL1β, CCL2, CXCL1/6, SERPINE1-F1, EFNB2, PLG, PF4, VEGFA, VEGFD, ANG, TGFβ1, EGF and HGF expression in human PC cells while upregulated CDH1, DKK3 and PTEN expression, leading to low proliferation and extensive ischemic necrosis. In the syngeneic PC model, IL30-targeting immunoliposomes downregulated NFKB1 expression and prevented intratumoral influx of CD11b^+^Gr-1^+^MDCs, Foxp3^+^Tregs, and NKp46^+^RORγt^+^ILC3, and prolonged host survival by inhibiting tumor progression. This study serves as a proof of principle that immunoliposome-based targeted delivery of Cas9gRNA-IL30 represent a potentially safe and effective strategy for PC treatment.

## Targeted immunotherapy suppresses prostate cancer growth and metastasis

Prostate cancer is an age-related disease and a leading cause of cancer death in men worldwide. Although its incidence has remained stable over the past decade, the proportion of tumors diagnosed in the advanced stages is rising, highlighting the need for improved and personalized treatments, especially for older patients with additional health problems. Di Carlo and coworkers created a NanoLiposome delivery system to transport a gene-editing tool, CRISPR/Cas9, directly to prostate cancer cells, to target and inhibit a particular gene involved in cancer growth. This study tested this approach’s effectiveness and safety on human prostate cancer cells and mouse models.　The findings show the potential of using NanoLiposomes for targeted gene editing in cancer. The researchers conclude that this method could provide a new and effective way to treat prostate cancer, particularly in patients requiring gentler treatment options.

This summary was initially drafted using artificial intelligence, then revised and fact-checked by the author.

## Introduction

Prostate cancer (PC) is a leading cause of cancer-related death in men worldwide. Although its incidence has remained stable over the past decade, a 4 to 6% annual increase in the incidence of advanced disease has been observed, and the proportion of patients diagnosed with PC in advanced stages has subsequently increased from 3.9 to 8.2%^1^. Furthermore, given that PC mainly affects patients older than 50 years of age, an increase in new cases is expected in the near future due to population aging. However, great strides in therapy have not been made.

The development of personalized treatments that are well-tolerated by elderly patients with comorbidities is a major challenge and would have a high impact on the health care system.

Recent advances in nanobiotechnology could fulfill these needs by providing safe and flexible nanostructures suitable for precision medicine^2^.

Nanoliposomes, or submicron bilayer lipid vesicles, are a state-of-the-art tool suitable for the targeted delivery of bioactive agents that can overcome the toxicity of traditional drugs^3^. Due to their biodegradability, high entrapment efficiency and possibility for surface functionalization, nanoliposomes are very promising *smart* drug delivery vehicles^4^ and are currently being investigated for the treatment of aggressive and highly metastatic tumors^5^.

We recently discovered the tumor-promoting potential of an immunoregulatory molecule, the p28 subunit of interleukin (IL)27, also known as IL30, which is expressed by activated macrophages and myeloid-derived suppressor cells^6–9^, but is also found as a membrane-anchored cytokine in the cytoplasmic membrane of cancer cells^10–12^.

IL30 expression has been detected in approximately 77% of metastatic PC patients, localized in cancer and/or infiltrating myeloid cells, and is correlated with disease grade and stage^7^. Targeted deletion of the *IL30* gene in PC cells using CRISPR/Cas9 genome editing inhibits a cascade of autocrine and paracrine signals, which foster cancer proliferation, migration, and immune evasion, resulting in tumor suppression and prolonged host survival^12^. CRISPR/Cas9 is the most developed and widely used tool for current genome editing strategies^13^, because it is highly specific, efficient, and versatile^14^, and has entered clinical trials for the treatment of different human diseases, such as hematological disorders, cancer, inherited eye disease, diabetes, infectious and inflammatory diseases, and protein-folding pathologies (https://innovativegenomics.org/news/crispr-clinical-trials-2022/). However, many challenges remain to be addressed to increase its efficacy, among which the delivery system plays a key role.

In this study, using a microfluidic device, we designed and synthesized a nonimmunogenic biocompatible cationic lipid nanocomplex coated with PEG (NxP) and conjugated with anti-PSCA antibodies (Abs) for the selective delivery of the Cas9 guide (g) RNA-IL30 complex to human (h)-derived PSCA^+^IL30^+^ PC xenografts and in a syngeneic, fully immunocompetent, PSCA^+^IL30^+^ PC model. We demonstrated the efficient uptake of Ab-conjugated Cas9gRNA-IL30 NxPs in PC cells both in vitro and in vivo, after systemic administration. The NxPs led to high-efficiency editing, tumor-selective suppression of IL30 signaling pathways and remodeling of the intratumoral immune cell context, resulting in substantial tumor growth inhibition and improved survival without evident toxicity. Our investigation provides a proof of concept that immunoliposome delivery of the Cas9gRNA-hIL30 complex for *IL30* genome editing at the tumor site is a clinically valuable tool for the safe and effective immunotherapy of PC.

## Materials and methods

### Cell lines

Human PC cell lines derived from the metastases of high-grade PCs, DU145^12,15^ and PC3^16^ cells, and the murine PC cell line TRAMP-C1 cells isolated from a 32-week-old C57BL/6J male TRAMP mouse^17^ were purchased from the ATCC (Manassas, VA, USA) and were authenticated using STR profile analysis. The generation of the *Il30* expression, or empty, lentiviral vector and its transfection into TRAMP-C1 cells to obtain IL30-TRAMP-C1 and EV-TRAMP-C1 cells were performed as described previously^12^.

The cell lines were cultured in RPMI-1640 with 10% FCS (Seromed, Biochrom KG, Berlin, Germany), confirmed to be mycoplasma-free by PCR analysis, and passaged for fewer than 6 months after initial thawing.

### CRISPR/Cas9-mediated *IL30* gene knockout in PC cells

CRISPR/Cas9 technology was used to generate *IL30* gene knockout (IL30KO) in both DU145 and PC3 cells, and abrogation of IL30 expression was validated by western blotting, as previously described^12^.

### Flow cytometry and antibody conjugation efficiency

PSCA expression on DU145, PC3, wild-type TRAMP-C1 and IL30-TRAMP-C1 cells and the conjugation efficiency between the anti-PSCA Abs and the aldehyde-modified DSPE-PEG2000 lipid present on the external layer of the NxP were assessed using flow cytometry, as described in the Supplementary Methods.

### Synthesis of nanoliposomes and Cas9IL30 NxPs and functionalization with anti-PSCA Abs

Cationic lipid nanocomplexes coated with PEG (NxP) were synthesized using a microfluidic device from Dolomite Microfluidics (Royston, UK) and lipids, as described in detail in the Supplementary Methods. All components were approved, for clinical use, by the European Medicines Agency (EMA) and Food and Drug Administration (FDA).

### Physical characterization of nanoliposomes

The size and zeta potential of the nanoliposomes were analyzed using dynamic laser light scattering (DLS). Their size and shape were also investigated using transmission electron microscopy (TEM), as described in the Supplementary Methods. Details on the assessment of their serum and pH stability are reported in the Supplementary Methods.

### Encapsulation efficiency and release rate of the Cas9IL30 NxPs

The encapsulation efficiency of Cas9gRNA-IL30 into nanoliposomes and the release rate of the Cas9IL30 NxPs were determined as reported in the Supplementary Methods.

### Editing quantification and analysis of off-target cleavage in vitro and in vivo

The true guide synthetic (s)gRNAs used for *IL30* gene editing were designed and synthesized by Thermo Fisher Scientific (Waltham, MA, USA) using a validated proprietary algorithm that predicts high-efficiency editing (Supplementary Figs. 1, 2 and Supplementary Table 1). Editing quantification and evaluation of off-target cleavage events for the selected sgRNAs (identified using COSMID software, https://crispr.bme.gatech.edu, and the CRISPR-Cas9 guide RNA design checker (https://eu.idtdna.com/site/order/designtool/index/CRISPR_SEQUENCE) (Supplementary Table 2) were performed as described in the Supplementary Methods. Whole-genome next-generation sequencing (NGS) was performed by Lexogen GmbH (Wien, Austria) using the Illumina NovaSeq 6000 platform system and Dragen software.

### Efficiency of genome editing in Cas9IL30-PSCA NxP-treated human and murine PC cells by tracking indels using decomposition analysis or inference of CRISPR edits

The efficiency of genome editing was quantified using the Tracking of Indels by Decomposition (TIDE) method in DU15 and PC3 cells or the Inference of CRISPR Edits (ICE) method in TRAMP-C1 cells, as previously reported^18,19^ and briefly described in the Supplementary Methods.

### Ultrastructural analyses of PC cells treated with Cas9IL30-PSCA NxPs

The intracellular uptake of NxPs by PC cells in vitro was analyzed using TEM, as reported in the Supplementary Methods.

### Cytotoxicity and proliferation assay

PC cell viability and proliferation after treatment with empty NxPs or Cas9IL30-loaded NxPs were assessed using the CellTiter 96 AQueous One Solution Cell Proliferation Assay (#G3582; Promega, Madison, WI, USA) according to the manufacturer’s instructions.

### Biosafety and distribution of nanoliposomes in vivo

- *Assessment of markers of organ and hematological toxicity, analyses of the immunogenicity of NxPs and analyses of weight changes due to the treatment* were performed as described in the Supplementary Methods. To assess the immunogenicity of NxPs and any weight changes due to treatment, four groups of five fully immunocompetent BALB/c male mice were intravenously (i.v.) injected with PBS, the naked Cas9hIL30 complex, empty-PSCA NxPs or Cas9hIL30-PSCA NxPs. Blood samples were collected at 6 and 24 hrs post injection to measure IL6 and TNFα levels, as described in the Supplementary Methods. Mice weight was constantly monitored for 63 days, consistent with the longest treatment schedule duration (12 administrations, 6 weeks), to assess the systemic toxicity of NxPs.
- *Histopathological and ultrastructural analyses of the intracellular uptake and toxicity of NxPs* in vivo were performed as described in the Supplementary Methods.
- *Laser scanning confocal (LSC) microscopy analyses of the lung transit and tumor uptake of the NxPs* were performed as described in the Supplementary Methods.

### Pharmacokinetic studies

For pharmacokinetic studies, 3 groups of 5 PC3 and 3 groups of 5 DU145 tumor-bearing NSG mice and 3 groups of 5 TRAMP-C1 tumor-bearing C57BL/6J mice were i.v. treated when the tumors reached a mean volume of ~700 mm^3^, with Cas9hIL30 NxPs or Cas9mIL30 NxPs conjugated or not conjugated with anti-PSCA Abs or free Cas9 (in PBS) at a dose of 1.5 mg of Cas9 equivalent/kg. Subsequently, blood was collected at predetermined time intervals (5 min, 15 min, 30 min, 1 hr, 2 hrs, 4 hrs, 6 hrs, 8 hrs, 10 hrs, and 12 hrs) for ELISA (Cas9 ELISA Kit, # PRB-5079, Cell Biolabs, San Diego, CA, USA).

### Magnetic resonance imaging (MRI)

The biodistribution of NxPs and their efficiency in targeting the tumor site were determined by using a 3T scanner (Philips Medical Systems, Nederland), as described in the Supplementary Methods.

### Western blot quantification of the tissue distribution of Cas9 protein

Analysis of Cas9 protein in tumors and organs from NSG mice treated with anti-hPSCA Ab-conjugated or unconjugated NxPs was performed as reported in the Supplementary Methods.

### In vivo testing of Cas9gRNA-IL30 NxP treatment in PC models

Eight-week-old NSG male mice were purchased from Charles River (Wilmington, MA, USA) and housed under high barrier conditions according to the Jackson Laboratory’s guidelines (https://www.jax.org/jax-mice-and-services/find-and-order-jax-mice/nsg-portfolio/housing-and breeding-considerations-for-nsg-mice) in the animal facility of the Center for Advanced Studies and Technology at the “G. d’Annunzio” University of Chieti-Pescara, Italy.

To study the effects of IL30 target treatment on tumor growth and progression, three groups of twenty-seven 8-week-old, NSG male mice were subcutaneously (s.c.) injected with 5 × 10^5^ wild-type (CTRL) DU145 cells or with 1 × 10^6^ PC3 cells. To study the effects of IL30-targeted treatment on the tumor immune microenvironment and tumor behavior, three groups of twenty-seven 8-week-old C57BL/6J male mice were s.c. injected with 5 × 10^5^ mIL30 lentiviral-DNA-transfected TRAMP-C1 cells (IL30-TRAMP-C1).

Mice were treated with Cas9IL30-PSCA NxPs, empty-PSCA NxPs, or PBS as soon as the tumors became palpable and twice a week thereafter, as shown in Supplementary Fig. 3. Two additional groups of twenty-seven 8-week-old NSG male mice were s.c. injected with nontargeting guide RNA-transfected (NTgRNA) or IL30 knockout (IL30KO) DU145 or PC3 cells, whereas an additional group of twenty-seven 8-week-old C57BL/6J male mice was s.c. injected with wild-type TRAMP-C1 cells. These control groups were monitored for tumor growth but were left untreated. Tumors were measured with calipers as soon as they were palpable (2 mm in diameter). Based on the tumor growth and progression rates, 12 mice from each group were euthanized at (4) key time points (3 mice per point) for histopathological and ultrastructural analyses. The remaining 15 mice per group were kept alive until tumors reached 2 cm^3^ or evidence of suffering was observed. Autopsy and histopathological examinations of the different organs (heart, liver, lungs, kidney, spleen and prostate) were performed.

An overall sample size of 15 mice per group allowed the detection of a statistically significant difference in tumor growth among the five groups (ANOVA) with 80% power at a 0.05 significance level (G*Power, RRID:SCR_013726). The animal procedures were performed in accordance with the European Community and ARRIVE guidelines and were approved by the Institutional Animal Care Committee of “G. d’Annunzio” University and by the Italian Ministry of Health (Authorization n. 892/2018-PR).

### Histology, immunohistochemistry, and TUNEL assays

Histology, immunohistochemistry (performed with the Abs listed in Supplementary Table 3), assessment of apoptotic (TUNEL assay) and proliferation (Ki-67 immunostaining) indices, and evaluation of the microvessel density (MVD) in tumors were performed as described in the Supplementary Methods.

### Statistics

For in vitro and in vivo studies, between-group differences were assessed using the Student’s *t* test or ANOVA, followed by Tukey’s HSD test. Survival curves were constructed using the Kaplan‒Meier method, and survival differences were analyzed using the log-rank test. All the statistical tests were evaluated at an α level of 0.05 using Stata, version 13 (StataCorp, College Station, TX, USA; RRID:SCR_012763). For MRI data assessment, the mean signal intensities of the tumor, prostate, lungs, liver, kidneys, spleen, heart, bones, and brain on both the T1- and T2-weighted images were calculated at scheduled time points for each group. The results are expressed as the mean ± standard deviation, and Prism 6 (GraphPad Software Inc., La Jolla, CA) was used to perform all the statistical analyses.

### Study approval

All animal procedures were performed in accordance with the European Community and ARRIVE guidelines and were approved by the Institutional Animal Care Committee of “G. d’Annunzio” University and by the Italian Ministry of Health (Authorization n. 892/2018-PR).

The human prostate tissue samples for immunostaining with anti-PSCA Abs were obtained from the institutional Biobank of the Local Health Authority n. 2 Lanciano - Vasto - Chieti (Italy). The personal data processing complied with the data protection laws.

## Results

### Cationic lipid nanocomplexes were loaded with Cas9gRNA-hIL30 to delete the *IL30* gene and functionalized by conjugation with anti-hPSCA Abs to target PC cells

The well-established antitumor efficacy of *IL30* gene deletion in PC cells^12^ has led to the development of a therapeutic approach to selectively inhibit its expression at the tumor site, minimizing the risk of side effects.

**Fig. 1:**
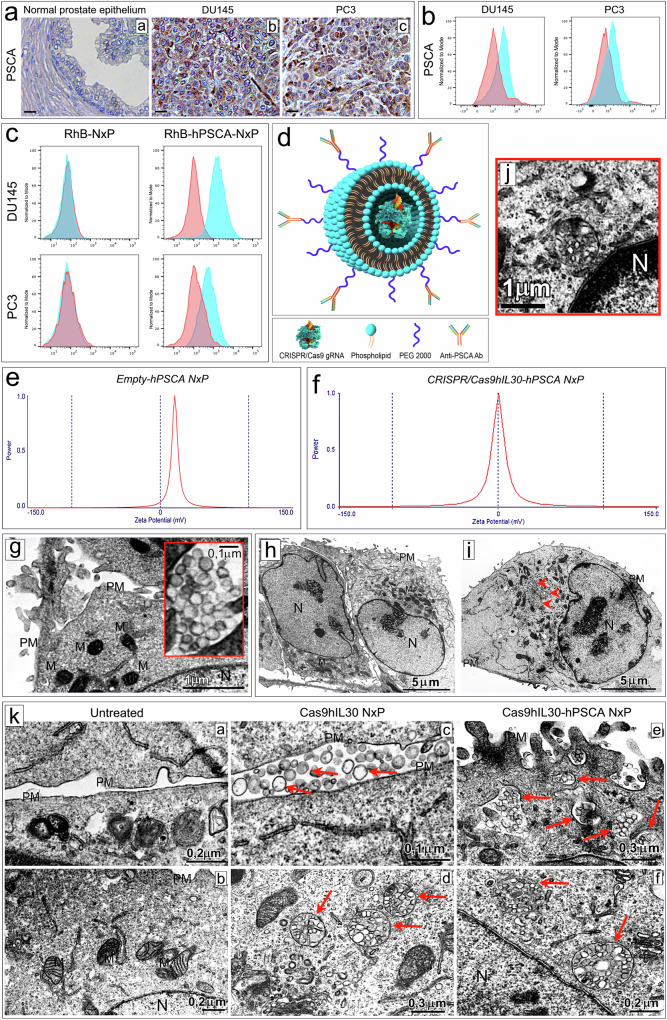
PSCA expression in PC3 and DU145 cells and specific binding and uptake of nanoliposomes conjugated with anti-hPSCA Ab by tumor cells. **a** PSCA expression is almost absent in the normal epithelium of the human prostate gland (a), while it is marked in the neoplastic epithelium of tumors that develop after s.c. implantation of DU145 (b) or PC3 (c) cells in NSG mice. Magnification: 630×. Scale bars: 10 µm. **b** Cytofluorimetric analyses of PSCA expression on the surface of DU145 (images on the left) and PC3 (images on the right) cells. Blue areas: specific Abs. Red areas: isotype controls. Representative images of the experiments performed in triplicate are shown (additional images from the flow cytometric analyses are shown in Supplementary Fig. 4a and b). **c** Specific binding of anti-hPSCA Ab-conjugated/rhodamine B-labeled nanoliposomes (RhB-hPSCA-NxP) to the surface of DU145 (top right picture) and PC3 (bottom right picture) cells compared to unconjugated/rhodamine B-labeled nanoliposomes (RhB-NxP) (top left and bottom left pictures). Blue areas: anti-hPSCA Ab-conjugated or unconjugated NxPs. Red areas: isotype controls. The experiments were performed in triplicate. **d** The immunoliposome used in this study consists of a bilayer phospholipid spheroid vesicle containing the CRISPR/Cas9gRNA-hIL30 complex and functionalized with anti-hPSCA Abs linked to PEGylated lipids. **e**, **f** DLS analysis of the zeta potential of empty-hPSCA NxP (29.17 ± 3.56 mV) (**e**) and of Cas9hIL30-hPSCA NxP (3.42 ± 1.49 mV) (**f**). **g** Electron microscopy analysis of nanoparticles cultured with DU145 cells showing their spherical shape and submicron size, and the high homogeneity of both features. On the right, a magnified detail of the image illustrating the ultrastructural features of the NxPs. PM: plasma membrane. M mitochondrion. N nucleus. The values of the scale bars are reported in the images. **h**–**j** Ultrastructural features of DU145 cells untreated (**h**) or treated with Cas9hIL30-hPSCA NxPs (**i**), which contain spherical-shaped vesicles composed of one or more phospholipid bilayers (arrowheads). **j** The image shows a magnification of one of these vesicles located close to the nuclear envelope. PM: plasma membrane. M mitochondrion. N nucleus. The values of the scale bars are reported in the images. **k** Ultrastructural features of DU145 cells that were not treated (a, b) or treated with Cas9hIL30 NxPs (c, d) or Cas9hIL30-hPSCA NxPs (e, f). Similar to that observed in the untreated PC cells (a, b), the treated cells contained intact mitochondria and endoplasmic reticulum (c–f) with no signs of ultrastructural damage to the cytoplasmic organelles. After 2 hrs of treatment, the unconjugated NxPs largely remained on the tumor cell surface (c, arrows), whereas the anti-hPSCA Ab-conjugated NxPs were endocytosed by the tumor cell and were mostly found in the cell cytoplasm (e, arrows). Three hours after treatment, spherical vesicles containing more phospholipid bilayers (arrows) were detected in the cytoplasm of both tumor cells treated with Ab-conjugated (f) and unconjugated (d) NxPs. These results are comparable to those obtained with PC3 cells. PM plasma membrane. M mitochondrion. N nucleus. The values of the scale bars are reported in the images.

Prostate stem cell antigen (PSCA) is a prostate-specific glycosylphosphatidylinositol-anchored protein that is minimally detected in normal prostatic tissue (Fig. 1a, a) and is upregulated in most PCs. Its expression is positively related to disease grade and stage and to androgen independence^20,21^. Both AR^+^DU145 and AR^-^PC3 cells, as well as tumors originating from their subcutaneous (s.c.) implantation in NSG mice (Fig. 1a, b and c), express PSCA (Fig. 1b, Supplementary Fig. 4a, b); therefore, to increase NxP accumulation at the tumor site, anti-human (h) PSCA Abs were conjugated to the PEG derivatives present on the external bilayer of preformed NxPs using an aldehyde-maleimide reaction. The specificity of the binding of the anti-hPSCA Ab-conjugated/rhodamine B-labeled NxP (RhB-hPSCA-NxP) to the PSCA on the surface of PC cells was determined using flow cytometry (Fig. 1c).

NxPs were loaded with the Cas9gRNA-hIL30 complex, which demonstrated a fair entrapment efficiency (58%) when considering the substantial size of the Cas9 protein (160 kDa), and then conjugated with anti-hPSCA Abs to generate Cas9gRNA-hIL30-hPSCA NxPs, hereinafter referred to as Cas9hIL30-hPSCA NxPs. Alternatively, NxPs were left empty and conjugated with anti-PSCA Abs to generate empty-hPSCA NxPs, which were used as controls.

DLS measurements of the average particle size and zeta electromotive force determined that the empty-hPSCA NxPs and Cas9hIL30-hPSCA NxPs were submicron vesicles (Fig. 1d–f; Supplementary Table 4). Both immunoliposomes had a spherical structure and uniform size, and no particle precipitation was observed. These features were confirmed by TEM analyses of PC cells treated with Cas9hIL30-hPSCA NxPs, which were identified as round nanostructures that were homogeneous in size (0.1 μm) and shape and in close contact with PC cells (Fig. 1g). More rapid cellular uptake was noted for Cas9hIL30-hPSCA NxPs than for unconjugated Cas9hIL30 NxPs given that after 2 hrs of coculture with PC cells, most of them had been endocytosed, whereas the unconjugated Cas9hIL30 NxPs appeared largely crowded on the cell surface, suggesting that conjugation with Abs improves nanoparticle uptake by cancer cells (Fig. 1h–k). By the third hour of coculture, both the Ab-conjugated and unconjugated NxPs had been endocytosed by the tumor cells.

### Cas9hIL30-hPSCA NxPs demonstrate genome editing efficiency in human PC cells, no off-target activity and high serum and pH stability

**Fig. 2:**
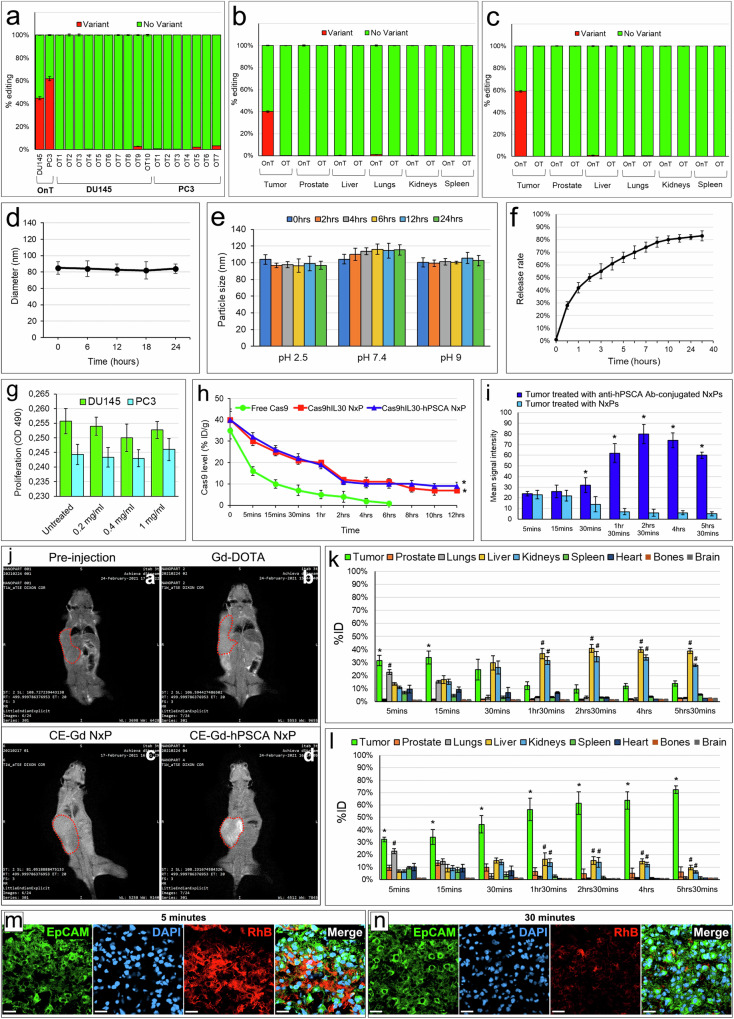
Physical characterization and biodistribution of Cas9hIL30-hPSCA NxPs. **a** On-target and off-target characterization of CRISPR/Cas9gRNA-mediated *hIL30* editing delivered using immunoliposomes in vitro. Average frequency of CRISPR/Cas9-induced variants in the *IL30* gene (editing efficiency or on-target effects, OnT) and in off-target sites corresponding to recognized genetic loci (off-target effects, OTs) in DU145 and PC3 cell cultures treated with Cas9hIL30-hPSCA NxPs. The frequency of variants (OnT and OTs) in cells treated with PBS or Empty-hPSCA NxPs (controls) was <0.1%. The experiments were performed in triplicate. **b**, **c** On-target and off-target characterization of CRISPR/Cas9gRNA-mediated *hIL30* editing by immunoliposomes in vivo. Average frequency of CRISPR/Cas9-induced variants in the *IL30* gene (editing efficiency or on-target effects, OnT) and in off-target sites (off-target effects, OTs) in the indicated organs of DU145 (**b**) and PC3 (**c**) tumor-bearing NSG mice treated with Cas9hIL30-hPSCA NxPs. The frequency of variants (OnT and OTs) in the organs of mice treated with PBS or empty-hPSCA NxPs (controls) was <0.1%. **d**, **e** Serum (**d**) and pH (**e**) stability of Cas9hIL30-hPSCA NxPs measured at different time points over a 24-h period. The experiments were performed in triplicate. **f** Cas9 release profile of Cas9gRNA-hIL30 NxP. The experiments were performed in triplicate. **g** Viability of DU145 (green bars) and PC3 (light blue bars) cells after 48 hrs of incubation with different concentrations (0.2, 0.4, or 1.0 mg/ml) of empty-hPSCA NxPs *versus* PBS-treated cells. ANOVA, *p* > 0.05. The results obtained from untreated cells were comparable to those from PBS-treated cells. The experiments were performed in triplicate. **h** Pharmacokinetics of free Cas9 and Cas9hIL30 NxPs, conjugated or not with anti-hPSCA Abs, in PC3 tumor-bearing NSG mice. %ID/g = percentage of total injection dose per weight. ANOVA, *p* < 0.001. *p < 0.01, Tukey HSD test *versus* free Cas9. **i** Quantitative analysis of T1-weighted MR images of tumors from mice treated with CE-Gd-hPSCA NxPs *versus* tumors from mice treated with CE-Gd NxPs at different time points after nanoparticle injection. ANOVA: *p* < 0.001. *p < 0.01, Tukey HSD test *versus* tumors of mice injected with unconjugated NxPs at the same time point. **j** In vivo T1-weighted MR images of tumor-bearing NSG mice before (a) and 2 hrs 30 min after intravenous injection with Gd-DOTA (b), CE-Gd NxPs (c) or CE-Gd-hPSCA NxPs (**d**). The tumors are indicated with red dotted circles. MRI data obtained from DU145 tumor-bearing mice are comparable to those obtained from PC3 tumor-bearing mice. All experiments were performed in triplicate. **k** Quantitative analysis of T1-weighted MR images of tumors *versus* organs in mice treated with CE-Gd NxPs at different time points after nanoparticle injection. Signal intensity is reported as a percentage of the injected dose (%ID). ANOVA: *p* < 0.001. *p < 0.01, Tukey HSD test *versus* organs at the same time point. ^#^*p* < 0.01, Tukey HSD test *versus* tumor and other organs at the same time point. **l** Quantitative analysis of T1-weighted MR images of tumors *versus* organs in mice treated with CE-Gd-hPSCA NxPs at different time points after nanoparticle injection. Signal intensity is reported as a percentage of the injected dose (%ID). ANOVA: *p* < 0.001. *p < 0.01, Tukey HSD test *versus* organs at the same time point. ^#^*p* < 0.01, Tukey HSD test *versus* other organs at the same time point. **m**, **n** Confocal microscopy images of lungs labeled with anti-EpCAM Abs (green) from DU145 tumor-bearing NSG mice, 5 min (**m**) and 30 min (**n**) after i.v. inoculation of RhB-hPSCA-NxPs (red). Similar results were obtained in mice inoculated with RhB-NxPs. DAPI: DNA-stained nuclei. Magnification: 400×. Scale bars: 10 µm.

One of the potential issues associated with CRISPR/Cas9 therapies is the genotoxicity caused by nonspecific genome cleavage. We evaluated cleavage specificity by performing deep sequencing of the off-target regions (Supplementary Table 2) of the selected single-guide RNAs (sgRNAs) in PC3 and DU145 cell cultures, and in NSG mice treated with Cas9hIL30-hPSCA NxPs (10 mg/ml, 48 hrs) or PBS. Our results showed that the frequency of variants of the off-target regions in cell cultures (Fig. 2a, Supplementary Fig. 5a) and in mice (Fig. 2b, c; Supplementary Fig. 5b, c) treated with Cas9hIL30-hPSCA NxPs was comparable to that observed in cells or mice treated with PBS (controls) (<0.1%), confirming the specificity of genome editing.

TIDE analyses revealed efficient genome editing in both DU145 cells and, particularly, in PC3 cells. In PC3 cells, the total editing efficiency of the targeted *IL30* gene was 63.8% for the forward strand and 62.6% for the reverse strand (Supplementary Fig. 6), whereas in DU145 cells, the total editing efficiency of the targeted *IL30* gene was 41.1% for the forward strand and 45.5% for the reverse strand (Supplementary Fig. 7). Deep sequencing of the *IL30* gene region in tumor tissues confirmed in vivo these data and allowed us to analyze the mutation patterns and the allelic mutation frequency of Cas9hIL30-hPSCA NxPs (Supplementary Figs. 8 and 9). The total editing efficiency of the targeted *IL30* gene was 59% in PC3 tumors and 40% in DU145 tumors (Fig. 2b, c).

Given that the physical stability of lipid nanocomplexes in the blood seriously affects their use as CRISPR/Cas9 drug delivery systems, we next evaluated the serum stability of our immunoliposomes by measuring overtime changes in their size in the presence of medium containing 10% FBS. Cas9hIL30-hPSCA NxPs exhibited great serum stability, and their size was relatively stable over a wide range of pH values, as measured over 24 hrs (Fig. 2d, e). This finding suggests that microenvironmental pH does not influence the physical stability of NxPs. As shown in Fig. 2f and Supplementary Fig. 10, at 30 min, the release rates of Cas9 by Cas9hIL30 NxPs and Cas9hIL30-hPSCA NxPs were 28% and 32%, respectively. These findings comply with the liposome formulation guidelines in the international pharmacopoeia (https://digicollections.net/phint/2020/index.html#d/b.1), which recommends a release rate of less than 40% at 0.5 hr.

Toxicity screening of the immunoliposomes assessed in both DU145 and PC3 cells exposed to increasing concentrations (0.2, 0.4, or 1 mg/ml) of empty-hPSCA NxPs for 48 hrs, revealed that the viability of PC cells treated with NxPs and PBS (or untreated) was comparable (ANOVA: *p* > 0.05), indicating that the immunoliposome formulation had no obvious toxicity (Fig. 2g).

Importantly, pharmacokinetic studies revealed that Cas9hIL30-hPSCA NxPs exhibited prolonged blood circulation, with a half-life (t1/2) of 1 hr, which was significantly longer than that of free Cas9, which was eliminated quickly from the bloodstream (t1/2 = 5 min) (ANOVA, *p* < 0.001) (Fig. 2h). Unconjugated NxPs showed a half-life comparable to that of Ab-conjugated NxPs (1 hr), suggesting that the prolonged circulation time was due to the lipidic shell, which efficiently protects the CRISPR/Cas9gRNA complex from enzymatic degradation. The pharmacokinetic results obtained from the DU145 tumor-bearing NSG mice were comparable to those from the PC3 tumor-bearing mice (Fig. 2h).

### Intravenously administered Cas9hIL30-hPSCA NxPs specifically targets and is efficiently taken up by PC xenografts

To assess the biodistribution of NxPs and their efficiency in selectively targeting the tumor site, magnetic resonance imaging (MRI) of tumor-bearing mice was performed before and 5 min, 15 min, 30 min, 1 hr 30 min, 2 hrs 30 min, 4 hrs, and 5 hrs 30 min after intravenous (i.v.) inoculation of core-encapsulated (CE) gadolinium (Gd) nanoliposomes conjugated or not with anti-PSCA Abs, herein referred to as CE-Gd-hPSCA NxP and CE-Gd NxP, respectively, or Gd-DOTA, which served as a control contrast agent. T1-weighted MR revealed that both CE-Gd-hPSCA NxP and CE-Gd NxP, as well as Gd-DOTA, were detectable in the tumor as early as 5 min after administration (Fig. 2i). A greater contrast effect was observed in the tumor xenografts of mice inoculated with CE-Gd-hPSCA NxPs than in the tumors of mice inoculated with CE-Gd NxPs starting 30 min after inoculation, and a progressive increase in the difference in signal intensity, which peaked at 2 hrs 30 min, was evident (Fig. 2i, j). The distribution of the Cas9 protein, as assessed by western blotting, in tumors from mice treated with Cas9hIL30-hPSCA NxPs *versus* tumors from mice treated with unconjugated NxPs, at different time points after nanoparticle injection, confirmed these data (Supplementary Fig. 11).

In animals treated with CE-Gd NxPs, the signal intensity, which is reported as the dose in tissue sample/injected dose × 100 (percentage of the injected dose (%ID), was significantly greater in the tumor than in the other organs 5 min and 15 min after i.v. injection and subsequently decreased (Fig. 2k). After NxP injection, the signal intensity progressively increased in the liver and kidney, the organs primarily involved in liposome metabolism and clearance^22^, in which a greater contrasting effect, compared to the other organs, was detected starting 1 hr 30 min after inoculation of CE-Gd NxPs or CE-Gd-hPSCA NxPs (Fig. 2k, l). Unlike tumors from mice that received unconjugated NxPs, tumors from mice inoculated with Abs-conjugated NxPs generated a consistently greater signal intensity than all other organs at all time points (Fig. 2l). Western blot (WB) quantification of the Cas9 protein in tumors *versus* organs from mice treated with unconjugated or anti-hPSCA Ab-conjugated NxPs, at different time points post injection, was consistent with the MRI results (Supplementary Figs. 12 and 13).

**Fig. 3:**
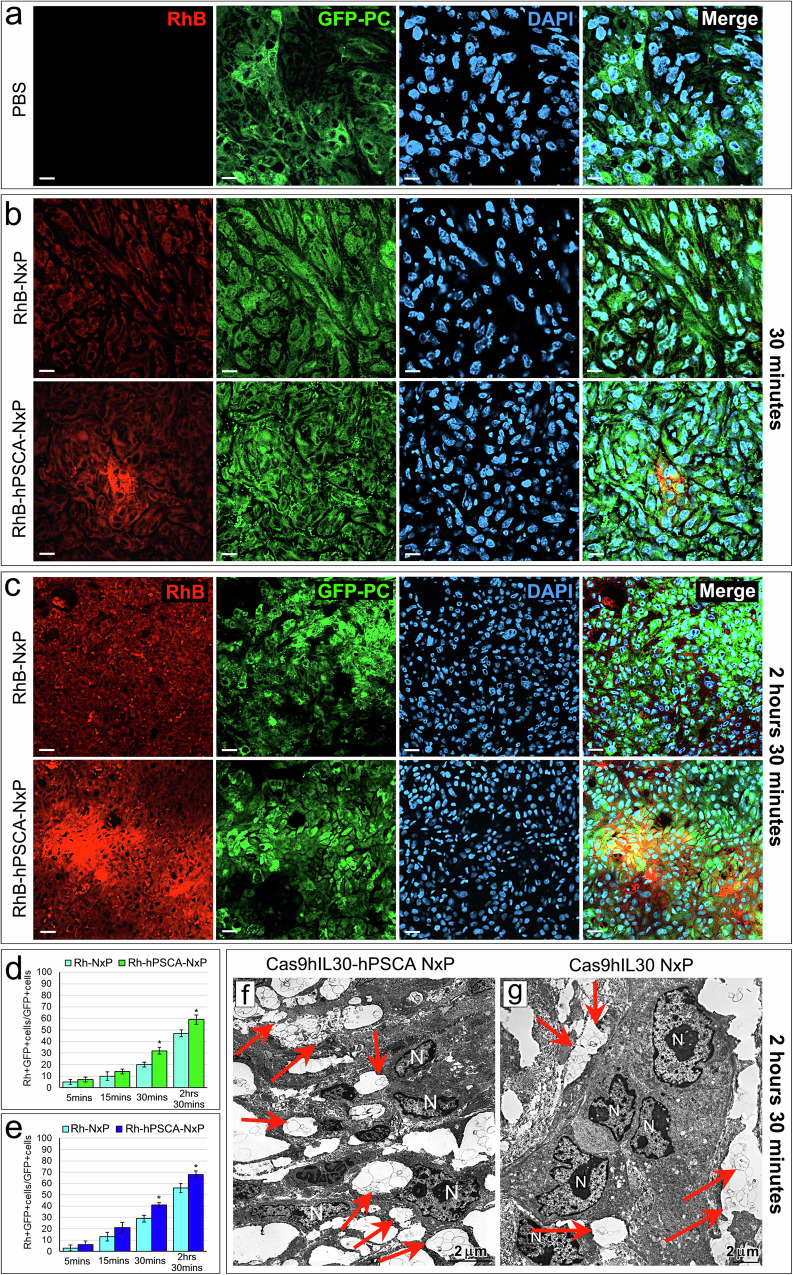
Tumor uptake of intravenously injected rhodamine B (RhB)-labeled NxPs (RhB-NxP) and RhB-labeled NxPs conjugated with anti-hPSCA Abs (RhB-hPSCA-NxP) in tumor-bearing NSG mice. **a**–**c** Confocal microscopy images of tumors from PBS-treated (**a**) or RhB-labeled nanoparticle-treated (**b**, **c**) mice showing RhB-hPSCA-NxP (red) or RhB-NxP (red) uptake by GFP-labeled DU145 tumors (green), 30 min (**b**) or 2 hrs 30 min (**c**) after i.v. inoculation of NxPs. Images of tumors from PBS-treated mice were comparable at each time point. Similar results were obtained for GFP-labeled PC3 tumor-bearing NSG mice inoculated with RhB-hPSCA-NxPs or RhB-NxPs. DAPI: DNA-stained nuclei. Magnification: 400×. Scale bars: 10 µm. **d**, **e** Quantification of NxP uptake in DU145 (**d**, green bars) and PC3 (**e**, blue bars) tumors that developed in NSG mice using LSC microscopy. Tumor uptake of the RhB-NxPs (light green or blue) and RhB-hPSCA-NxPs (dark green or blue) is expressed as the mean percentage ± SD of RhB+GFP+ cells/total number of GFP+ cells. *p < 0.01, Student’s *t* test vs RhB-NxPs at the same time point. **f**, **g** TEM images of tumors 2 hrs 30 min after treatment of mice with Cas9hIL30-hPSCA NxPs (**f**) or Cas9hIL30 NxPs (**g**) showing that, at this time point, unconjugated NxPs (arrows) were poorly endocytosed and predominantly localized among tumor cells. In contrast, the majority of anti-hPSCA Ab-conjugated NxPs were endocytosed and primarily found in the cell cytoplasm (arrows). N nucleus. Scale bars: 2 μm.

NxP uptake by PC xenografts was also investigated using laser scanning confocal (LSC) microscopy and TEM. LSC images of lungs from GFP-labeled DU145 or PC3 tumor-bearing NSG mice inoculated i.v. with rhodamine B (RhB)-labeled NxP conjugated or not with anti-PSCA Abs, namely, RhB-hPSCA-NxP and RhB-NxP, respectively, showed a distinct RhB fluorescence signal 5 min after inoculation, its strong decline 10 min later, and its nearly lost after 30 min (Fig. 2m, n). No signs of intravascular aggregation of NxPs were observed. A faint RhB signal was detected in the tumors as early as 5 min post-NxP inoculation, but its intensity progressively increased and differed substantially between the RhB-NxP- and RhB-hPSCA-NxP-treated tumors, which generated a stronger signal starting 30 min after injection and peaking 2 hrs 30 min after injection (Fig. 3a–e), confirming the MRI data. At this time point (i.e., 2 hrs 30 min post NxP injection), TEM images of the tumors (Fig. 3f, g) revealed that the uptake and internalization of NxPs in neoplastic cells were very efficient when they were conjugated with anti-hPSCA Abs (Fig. 3f). In contrast, the uptake and internalization of NxPs was poor when they were not conjugated (Fig. 3g) because unconjugated NxPs remained largely located among the neoplastic cells.

### Cas9hIL30-hPSCA NxP treatment inhibits tumor growth and improves survival in NSG mice bearing human PC xenografts

Given that MRI and LSC microscopy revealed an increased ability of Ab-conjugated *versus* unconjugated nanoliposomes to selectively reach tumor masses expressing PSCA (Fig. 1a, b and c), the effects of anti-hPSCA Ab-conjugated Cas9hIL30 NxPs were compared to those of anti-hPSCA Ab-conjugated empty NxPs to assess the antitumor potential of targeted delivery of the Cas9gRNA-hIL30 complex.

**Fig. 4:**
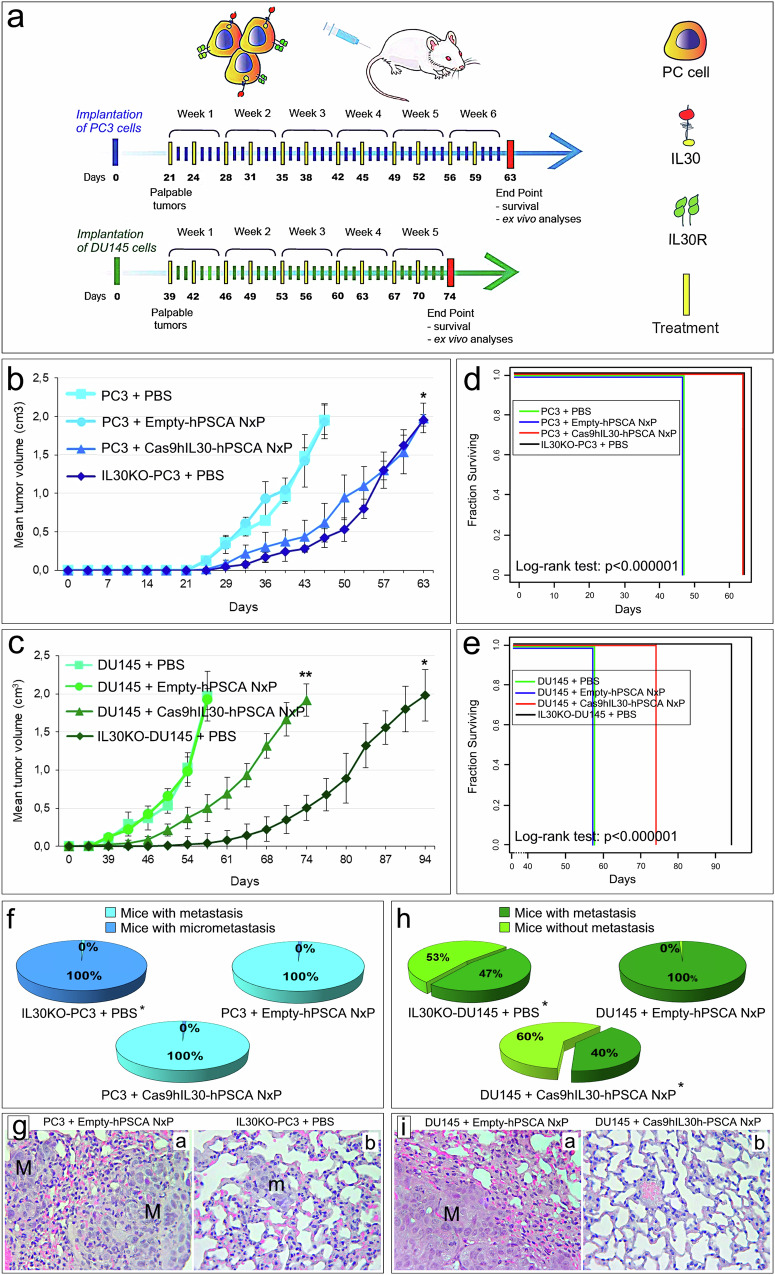
Tumor growth and survival in PC xenograft-bearing mice treated with Cas9hIL30-hPSCA NxPs. **a** Treatment schedules for NSG mice bearing human-derived subcutaneous PC3 (blue arrow) or DU145 (green arrow) tumors that express membrane-anchored IL30. In both xenograft models, treatment with two weekly doses of NxPs (10 mg/ml) started when the tumors were palpable (Ø 2 mm) and stopped when the tumors reached 2 cm^3^ and the mice were sacrificed. **b** The mean volume of subcutaneous tumors developed in 4 groups of fifteen NSG male mice after s.c. implantation of IL30KO or wild-type PC3 cells and treatment with PBS, empty-hPSCA NxPs, or Cas9hIL30-hPSCA NxPs. ANOVA: *p* < 0.0001. *p < 0.01, Tukey HSD test *versus* PC3 tumors treated with PBS or empty-hPSCA NxPs. The results obtained from tumors treated with Cas9-NTgRNA-hPSCA NxPs were comparable to those from tumors treated with empty-hPSCA NxPs. The results are expressed as the mean ± SD. **c** The mean volume of subcutaneous tumors developed in 4 groups of 15 NSG male mice after s.c. implantation of IL30KO or wild-type DU145 cells and treatment with PBS, empty-hPSCA NxPs, or Cas9hIL30-hPSCA NxPs. ANOVA, *p* < 0.0001. *p < 0.01, Tukey HSD test *versus* DU145 tumors treated with PBS or empty-hPSCA NxPs or Cas9hIL30-hPSCA NxPs. **p < 0.01, Tukey HSD test *versus* DU145 tumors treated with PBS or with empty-hPSCA NxPs. The results obtained from tumors treated with Cas9-NTgRNA-hPSCA NxPs were comparable to those from tumors treated with empty-hPSCA NxPs. The results are expressed as the mean ± SD. **d** Kaplan–Meier survival curves of 4 groups of fifteen NSG male mice bearing tumors developed after s.c. implantation of IL30KO PC3 cells or wild-type PC3 cells and treated with empty-hPSCA NxPs, Cas9hIL30-hPSCA NxPs or PBS. Mice bearing wild-type PC3 tumors, treated with Cas9hIL30-hPSCA NxPs, and mice bearing IL30KO-PC3 tumors survived longer than mice bearing wild-type PC3 tumors, treated with PBS or empty-hPSCA NxPs (63 *versus* 47 days; log-rank test: *p* < 0.000001). Mice were sacrificed when tumors reached 2 cm^3^ in size. **e** Kaplan–Meier survival curves of 4 groups of fifteen NSG male mice bearing tumors developed after s.c. implantation of IL30KO DU145 cells or wild-type DU145 cells and treated with empty-hPSCA NxPs, Cas9hIL30-hPSCA NxPs or PBS. Mice bearing wild-type DU145 tumors treated with Cas9hIL30-hPSCA NxPs survived longer than mice bearing wild-type DU145 tumors treated with PBS or empty-hPSCA NxPs (74 *versus* 58 days) but less than mice bearing IL30KO-DU145 tumors (94 days) (log-rank test: *p* < 0.000001). The mice were sacrificed when the tumors reached 2 cm^3^ in size. **f** Percentages of mice which developed lung metastases (>500 µm) or micrometastases (≤500 µm) in wild type (or IL30KO) PC3 tumor-bearing NSG mice, treated with Cas9hIL30-hPSCA NxPs or Empty-hPSCA NxPs. The results from PBS-treated wild-type PC3 tumor-bearing mice were comparable to those from empty-NxP-treated mice. *Fisher’s exact test, *p* = 0.0000000012 *versus* mice bearing wild-type PC3 tumors and treated with PBS, empty-hPSCA NxPs or Cas9hIL30-hPSCA NxPs. **g** Histopathological features of lung metastasis (M) that developed in empty-hPSCA NxP-treated mice bearing PC3 tumors (a), and lung micrometastases (m) that developed in PBS-treated mice bearing IL30KO-PC3 tumors (b). Magnification: 400×. Scale bars: 10 µm. **h** Percentage of lung metastases spontaneously developed in wild-type (or IL30KO) DU145 tumor-bearing NSG mice treated with Cas9hIL30-hPSCA NxPs or empty-hPSCA NxPs. The results from PBS-treated wild-type DU145 tumor-bearing mice were comparable to those from empty-hPSCA NxP-treated mice. *Fisher’s exact test, *p* = 0.0003 *versus* mice bearing wild-type DU145 tumors and treated with PBS or Empty-hPSCA NxPs. **i** Histopathological features of lung metastasis (M) in empty-hPSCA NxP-treated mice bearing DU145 tumors (a) and healthy lung tissue from Cas9hIL30-hPSCA NxP-treated mice bearing DU145 tumors, which had not developed metastases (b). Magnification: 400×. Scale bars: 10 µm.

The treatment schedule consisted of two i.v. injections of immunoliposomes per week (250 µl dose with a 10 mg/ml lipid concentration) starting from the onset of a palpable tumor (~2 mm Ø) and continuing throughout the lifespan of the mice (Fig. 4a).

To compare the effects on tumor growth and progression of the immunoliposome-based delivery of CRISPR/Cas9gRNA-hIL30 complex *versus* a direct *IL30* gene editing in PC cells, which generated IL30KO-DU145 and IL30KO-PC3 cells, in addition to three groups of NSG (NOD scid gamma) mice s.c. implanted with wild-type DU145 or PC3 cells, and then treated with Cas9hIL30-hPSCA NxPs, or empty-hPSCA NxPs, or PBS; two groups of mice implanted with IL30KO-DU145 or IL30KO-PC3 cells, and then treated with PBS, were included in the study.

In both PC xenograft models, treatment with Cas9hIL30-hPSCA NxPs substantially inhibited tumor growth compared to treatment with empty-hPSCA NxPs or PBS (ANOVA: *p* = 0.0001; Fig. 4b, c). The growth curve of PC3 tumors in mice treated with Cas9hIL30-hPSCA NxPs matched that of IL30KO-PC3 tumors. In both groups, the mean tumor volume was lower than that in PBS- or empty-hPSCA-treated mice (ANOVA: *p* = 0.0001; Fig. 4b). Kaplan–Meier analysis revealed that both groups of mice survived longer than mice treated with PBS or empty-hPSCA NxPs (63 *versus* 47 days, log-rank test: *p* < 0.000001; Fig. 4d). DU145 tumor-bearing mice, treated with Cas9hIL30-hPSCA NxPs, also survived longer than mice treated with PBS or empty-hPSCA NxPs (74 *versus* 58 days, log-rank test: *p* < 0.000001) but less than mice bearing slow-growing IL30KO-DU145 tumors (94 days) (Fig. 4c, e).

In the PC3 model, Cas9hIL30-hPSCA NxP treatment had no effect on lung metastases (lesions >500 µm in size). Although the IL30KO tumor-bearing mice had only micrometastases (≤500 µm in size, ref. ^23^), all the mice eventually developed metastases (Fig. 4f, g, Supplementary Fig. 14). In the DU145 model, 60% (9/15) of the mice treated with Cas9hIL30-hPSCA NxPs and 53% (8/15) of the mice bearing IL30KO tumors were metastasis free, whereas all the mice treated with empty-hPSCA NxPs or PBS developed lung metastases (Fisher’s exact test: *p* = 0.0003; Fig. 4h, i). In mice that developed metastatic disease, no difference was observed in the mean number of metastases per mouse between the groups (Supplementary Table 5).

### Cas9hIL30-hPSCA NxPs dramatically suppresses IL30 expression and inhibits proliferation, vascularization, and cancer driver genes in PC xenografts without evidence of toxicity or immunogenicity

**Fig. 5:**
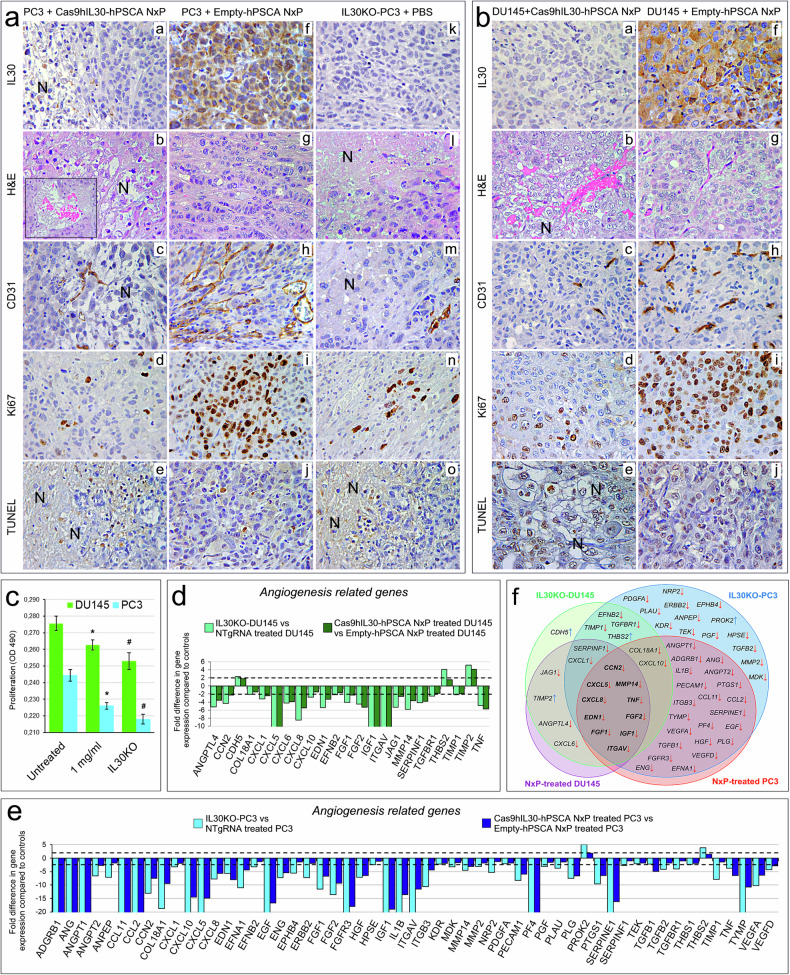
Immunopathological and molecular features of PC xenografts from mice treated with Cas9hIL30-hPSCA NxPs or Empty-hPSCA NxPs. **a**, **b** Subcutaneous PC3 (**a**) and DU145 (**b**) tumors from Cas9hIL30-hPSCA NxP-treated animals show the absence of IL30 expression (a and b, a), multiple areas of vascular leakage (see the inset in **a**, **b**) and ischemic-coagulative necrosis (a and b, b) associated with defective vascularization (a and b, c) and low cancer cell proliferation (a and b, d), as observed in IL30KO tumors from PBS-treated mice (a, k–n). In contrast, tumors from Empty-hPSCA NxP-treated mice expressed IL30 (a, f; b, f), lacked ischemic and hemorrhagic damage (a, g; b, g), and exhibited a well-developed vascular network (a, h; b, h) and robust proliferative activity (a, i; b, i). Apoptotic events were comparable in tumors from the different treatment groups (a, e and j, o; b, e and j). The results obtained from wild-type PC3 or DU145 tumors developed in PBS-treated mice were comparable to those of tumors from empty-PSCA-treated mice. N, necrosis. Magnification: 400×. Scale bars: 10 µm (inset in **a**, **b**, 15 µm). **c** DU145 (green bars) and PC3 (light blue bars) cell viability after 72 h of incubation with (1.0 mg/ml) Cas9hIL30-hPSCA NxPs *versus* untreated and IL30KO cells. ANOVA: *p* < 0.001. *p < 0.01, Tukey HSD test *versus* untreated cells. ^#^*p* < 0.01, Tukey HSD test *versus* Cas9hIL30-hPSCA NxP-treated cells and untreated cells. The results from untreated cells were comparable to those obtained from Empty-hPSCA NxP-treated cells. The experiments were performed in triplicate. **d**, **e** Human angiogenesis PCR array. Fold differences in the mRNAs of angiogenesis-related genes between IL30KO-DU145 cells and control NTgRNA-treated DU145 cells (**d**; light green bars) or PC3 cells (**e**; light blue bars) and between Cas9hIL30-hPSCA NxP-treated DU145 cells and control empty-hPSCA NxP-treated DU145 cells (**d**; dark green bars) or PC3 cells (**e**; dark blue bars). A significant threshold of a twofold change in gene expression corresponded to *p* < 0.001. Only genes with a fold change >2 are shown. The experiments were performed in duplicate. The dashed lines represent the twofold change cutoff. **f** Venn diagram representing the “*Angiogenesis Driver Genes*”, which are downregulated (red arrows pointing downward) or upregulated (blue arrows pointing upward) by IL30KO in DU145 (green circle) and PC3 cells (blue circle) and by Cas9hIL30-hPSCA NxP treatment of DU145 (purple circle) and PC3 cells (red circle). Overlapping circles illustrate the shared IL30-regulated genes between the different *IL30* gene-editing approaches in the different cell lines.

Immunopathological analysis of DU145 and PC3 tumors revealed a lack of IL30 expression following Cas9hIL30-hPSCA NxP treatment, as observed in IL30KO tumors, and extensive areas of ischemic hemorrhagic necrosis in association with poor vascularization and reduced tumor cell proliferation (Fig. 5a, b and Supplementary Table 6), compared with tumors from PBS- or empty-hPSCA NxP-treated mice. In addition, few apoptotic events occurred, as found in tumors from PBS- or empty-hPSCA NxP-treated mice (Supplementary Table 6). The histopathological features of wild-type tumors from Cas9hIL30-hPSCA NxP-treated mice and IL30KO tumors from PBS-treated mice were comparable (Fig. 5a, b). Macrophage infiltration, mostly localized to the tumor edge, was comparable between DU145 or PC3 tumors from mice treated with empty or Cas9hIL30-loaded immunoliposomes or with PBS, suggesting the non-immunogenicity of the NxP formulations (Supplementary Fig. 15).

The molecular events driving Cas9hIL30-hPSCA NxP-dependent inhibition of tumor proliferation and angiogenesis were investigated by assessing PC cell viability and angiogenic profile in response to nanoliposomes. DU145 and PC3 cell proliferation was substantially inhibited after 72 hrs of treatment with Cas9hIL30-hPSCA NxPs. These results were similar to that obtained with CRISPR/Cas9gRNA-mediated editing of the *IL30* gene in PC cells (Fig. 5c). Albeit to a lesser extent when compared to CRISPR/Cas9gRNA-mediated *IL30* gene editing, nanoliposome-based delivery of CRISPR/Cas9gRNA*-*hIL30 complex dramatically inhibited the angiogenic potential of both PC cell lines, because it reshaped their transcriptional profiles and downregulated a wide range of proangiogenic genes, including *ANGPTL 1/2/4, IL1β, CCL2, CXCL1, CXCL6, SERPINE1, SERPINF1, EFNB2, PLG, PF4, VEGFA, VEGFD, ANG, TGFβ1, EGF,* and *HGF*, and a few negative regulators of vascular network formation, such as *ADGRB1, COL18A1, CXCL10,* and *TIMP2* (Fig. 5d, e). The substantial downregulation of *IGF1, CCN2, CXCL5, CXCL8, EDN1, ITGAV, TNF, MMP14, FGF1,* and *FGF2* in both DU145 and PC3 cells was a shared effect between direct and nanoliposome-mediated *IL30* gene editing (Fig. 5f). Immunopathological detection of the downregulation, or lack of expression, of angiogenic drivers in IL30KO tumors and nanoparticle-treated tumors substantiated the molecular in vitro findings.

**Fig. 6:**
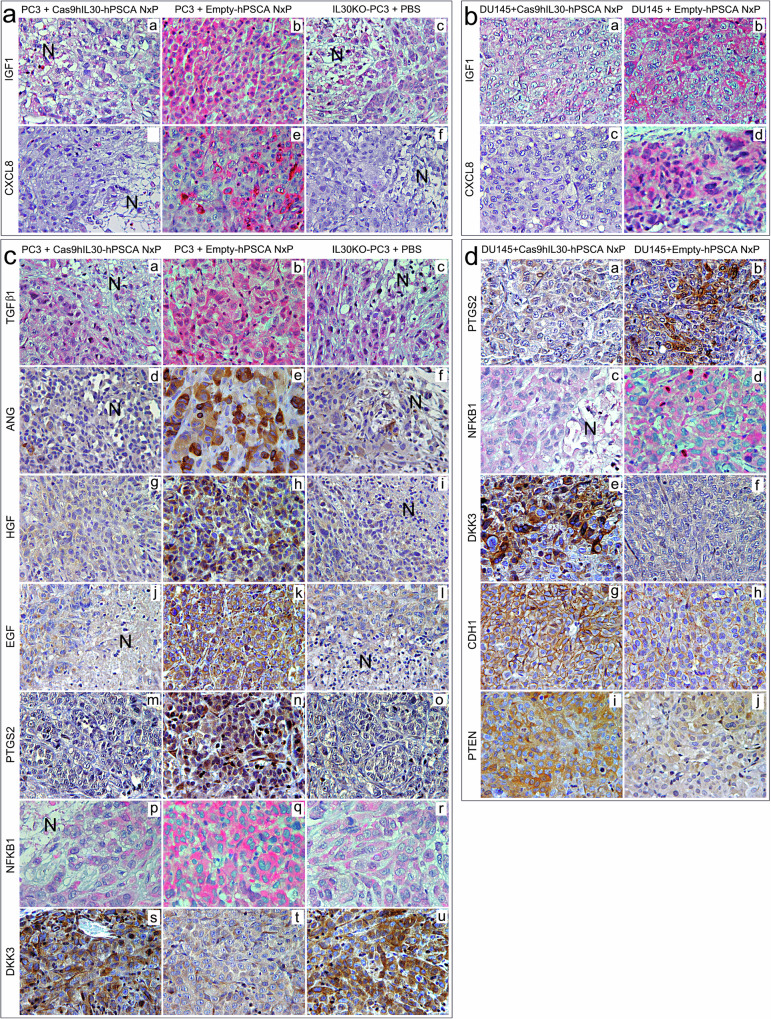
Immunopathological aspects of PC xenografts from mice treated with Cas9hIL30-hPSCA NxPs or Empty-hPSCA NxPs. **a**, **b** Expression of the proangiogenic genes IGF1 (g, a and b, c; h, a and b) and CXCL8 (g, d–f; h, c and d) was minimal to absent in Cas9hIL30-hPSCA NxP-treated PC3 (g, a and d) and in Cas9hIL30-hPSCA NxP-treated DU145 (h, a and c) tumors compared to empty-hPSCA NxP-treated PC3 (g, b and e) and empty-hPSCA NxP-treated DU145 (h, b and d) tumors, respectively. The immunohistochemical features of Cas9hIL30-hPSCA NxP-treated PC3 tumors (g, a and d) were similar to those observed in PBS-treated IL30KO-PC3 tumors (g, c and f). The immunohistochemical features of the control tumors that developed in the NSG after implantation of the NTgRNA-treated cells were comparable to those of the empty-hPSCA NxP-treated tumors and to those of the untreated wild-type tumors. N: necrosis. Magnification: 400×. Scale bars: 10 µm. **c**, **d** Subcutaneous PC3 (a) and DU145 (b) tumors from Cas9hIL30-hPSCA NxP-treated animals were characterized by marked inhibition of proangiogenic genes, such as TGFβ1 (a, a), ANG (a, d), HGF (a, g), EGF (a, j), and PC driver genes, such as PTGS2 (a, m; b, a) and NFKB1 (a, p; b, c), whereas tumor suppressors, such as DKK3 (a, s; b, e), CDH1/E-Cadh (b, g) and PTEN (b, i), were strongly expressed compared to empty-hPSCA NxP-treated tumors (a: b, e, h, k, n, q, t; b: b, d, f, h, j). The immunopathological features of Cas9hIL30-hPSCA NxP-treated PC3 tumors (a: a, d, g, j, m, p, s) were similar to those of PBS-treated IL30KO-PC3 tumors (a: c, f, l, o, r, u). The immunopathological features of control tumors that developed in NSGs after implantation of NTgRNA-treated cells were comparable to those of empty-hPSCA NxP-treated tumors and untreated wild-type tumors. N Necrosis. Magnification: 400× (NFKB1 immunostaining, 630×). Scale bars: 10 µm (NFKB1 immunostaining, 5 µm).

In addition to IL30 expression, treatment with Cas9hIL30-hPSCA NxPs effectively suppressed the expression of previously identified IL30-regulated oncogenes^12^, such as *IGF1* (Fig. 6a, b; Supplementary Tables 7 and 8), which also functions as an angiogenic factor, and *PTGS2* and *NFKB1*, which promote cancer cell survival and inflammation-associated cancer^24^. In contrast, the expression of tumor suppressors, such as *CDH1, DKK3,* and *PTEN* (which were assessed only in DU145 tumors because PC3 cells have a homozygous deletion of the *PTEN* gene and are therefore negative for *PTEN* expression, ref. ^25^), was markedly upregulated, as we described previously in IL30KO-DU145 tumors^12^ and confirmed here in IL30KO-PC3 tumors (Fig. 6c, d; Supplementary Tables 7 and 8).

**Fig. 7:**
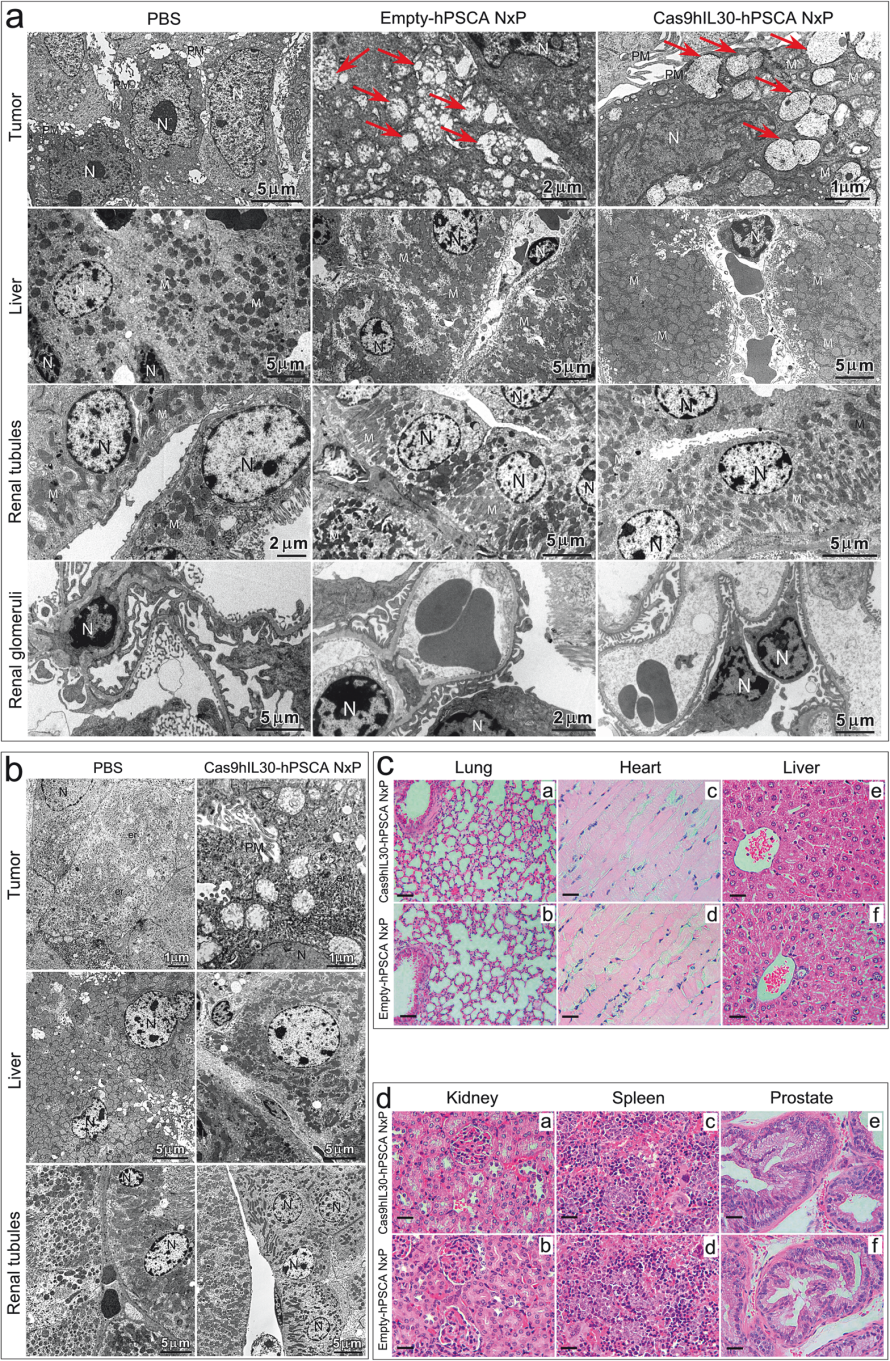
Morphological features of PC xenografts and organs of mice treated with Cas9hIL30-hPSCA NxPs or empty-hPSCA NxPs. **a**, **b** TEM analyses of subcutaneous tumor xenografts, liver, and kidneys from PC3 (**a**) and DU145 (**b**) tumor-bearing NSG mice treated with Cas9hIL30-hPSCA NxPs, empty-hPSCA NxPs or PBS. Compared with the tumors of the PBS-treated mice (**a**, **b**), the tumors of the empty-hPSCA NxP- or Cas9hIL30-hPSCA NxP-treated mice exhibited cytoplasmic vacuoles that were consistent with aspects of nanoparticle endocytosis (arrows). The liver, glomeruli, and renal tubules of treated mice showed no signs of cell damage or ultrastructural features comparable to those of the respective organs of PBS-treated mice. N nucleus. M mitochondrion. PM plasma membrane. The values of the scale bars are reported in the images. **c**, **d** H&E staining showing that the lungs, heart, liver (**c**), kidneys, spleen, and prostate (**d**) of DU145 tumor-bearing NSG mice treated with Cas9hIL30-hPSCA NxPs (a, c, e) are histologically normal, with no signs of cell damage, and similar to those observed in empty-hPSCA NxP-treated mice (b, d, f). Similar results were obtained from the histopathology of the organs of PBS-treated mice. Magnification; **c**, a and b: 200×; **c**, c–f: 400×; **d**, a–f: 400×. Scale bars: **c** (a, b): 25 µm; **c** (c–f) and **d** (a–f): 15 µm.

Ultrastructural analyses, using TEM, revealed that both tumors treated with empty-hPSCA NxPs and those treated with Cas9hIL30-hPSCA NxPs showed intracytoplasmic spherical membranes. These membranes were visualized both in the peripheral and deep areas of the tumor masses, but no signs of damage to the cytoplasmic organelles or cytotoxicity were detected in non necrotic neoplastic areas (Fig. 7a, b).

Histopathological analyses, performed both at the end of the experiment and at different time points of the treatment, showed that the lungs, heart, liver, kidney, spleen, and prostate from Cas9hIL30-hPSCA NxP- or empty-hPSCA NxP-treated animals were histologically normal and comparable to the organs obtained from PBS-treated animals (Fig. 7c, d). TEM investigations of the liver (to monitor the main organ that expresses key enzymes for lipid metabolism) and kidney tissues (to assess the risk of lipotoxic renal damage) confirmed the absence of cellular damage at the ultrastructural level (Fig. 7a, b).

The safety of Cas9hIL30*-*hPSCA NxPs was further assessed using serological, hemolytic, and immunogenicity tests. To assess the toxicity of the treatment in specific organs, the levels of the following markers were measured 21 days after starting treatment (after 6 treatments): aspartate aminotransferase (AST) and alanine aminotransferase (ALT), which are markers of liver function; blood urea nitrogen (BUN) and creatinine (Cr), which are markers of renal function; and cardiac troponin-1 (cTnI), creatine kinase (CK) and lactate dehydrogenase (LDH), which are markers of cardiac function. The serum parameters of the animals treated with empty-hPSCA NxPs or Cas9hIL30-hPSCA NxPs were comparable to those of the PBS-treated mice, thus indicating the lack of specific organ damage (Supplementary Table 9). Furthermore, as shown in Supplementary Fig. 16, Cas9hIL30-hPSCA NxPs had no obvious hemolytic effect.

Assessment of NxP immunogenicity in fully immunocompetent BALB/c mice harboring the genetic background of the NSG mice used for treatment revealed that i.v. inoculation of naked Cas9gRNA-hIL30 complex, empty-hPSCA NxPs, or Cas9hIL30-hPSCA NxPs stimulated only minimal TNFα and IL6 responses, which returned to baseline PBS levels 24 hrs post injection. These findings suggest that the nanoliposomes, or their Cas9gRNA-hIL30 content, had no lasting adverse immune or inflammatory activity. In addition, the average weight of mice treated with naked Cas9gRNA-hIL30 complex, empty-hPSCA NxPs, unconjugated Cas9hIL30 NxPs or Cas9hIL30-hPSCA NxPs was comparable (ANOVA: *p* > 0.05) to that of mice treated with PBS (Supplementary Table 10), strongly suggesting the absence of systemic toxicity^26^. Overall, the immunoliposome-based targeted delivery of the Cas9gRNA-hIL30 complex, which selectively inhibits tumor IL30 expression, has antiangiogenic and antiproliferative effects in both PC models and has antimetastatic efficacy in DU145 tumor model, without evidence of hematological or organ toxicity.

### Immunoliposomes loaded with CRISPR/Cas9gRNA to delete murine *Il30* exhibit genome editing efficiency in murine PC cells without off-target or cytotoxic effects

To assess whether IL30-targeting immunoliposomes could reverse the immunosuppressive effects of IL30^9,11,27^ and restore antitumor immunity, we tested this therapeutic approach in a fully immunocompetent syngeneic murine model involving the s.c. implantation of TRAMP-C1 cells engineered to overexpress IL30 (IL30-TRAMP-C1, the clone releasing 458,00 ± 12,06 ng/mL of mIL30, ref. ^14^). These cells produced rapidly progressing and highly metastatic tumors, resulting in decreased survival compared to that of mice bearing control tumors (wild-type TRAMP-C1 and EV-TRAMP-C1).

**Fig. 8:**
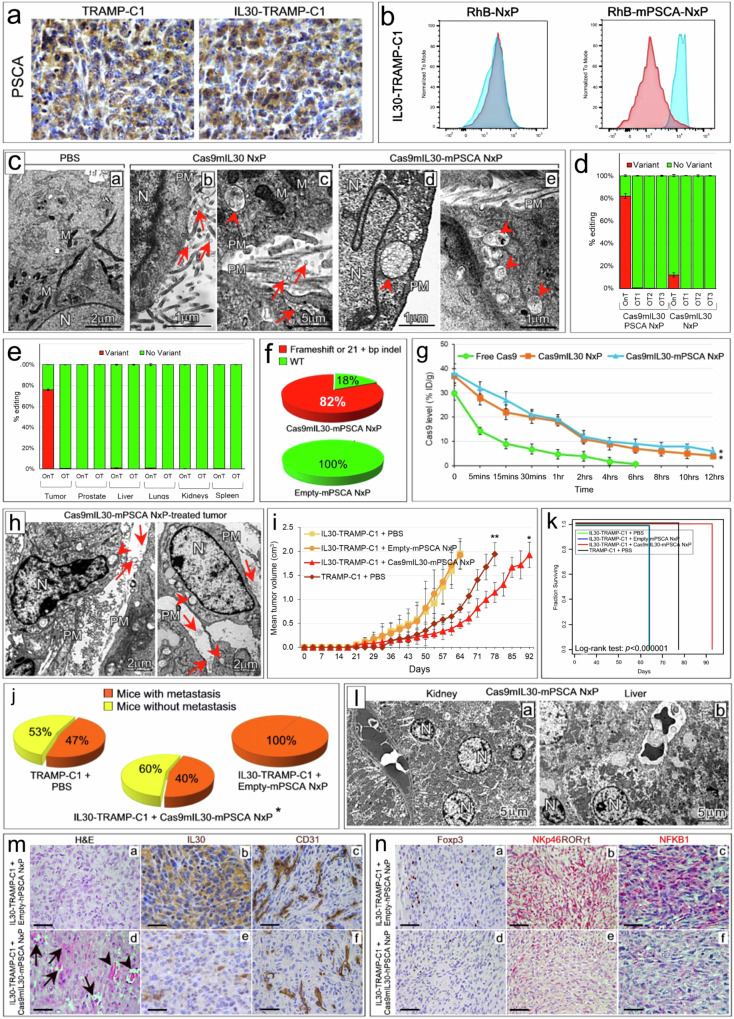
Physical characterization and biodistribution of Cas9mIL30-mPSCA NxPs and their effect on tumor growth and survival in a fully immunocompetent host. **a** PSCA expression in tumors developed after s.c. implantation of wild-type TRAMP-C1 and IL30-TRAMP-C1 cells in syngeneic C57BL/6J mice. Magnification: 630×. Scale bars: 10 µm. **b** Specific binding of anti-mPSCA-conjugated/rhodamine B-labeled nanoliposomes (RhB-mPSCA-NxPs) to the surface of IL30-TRAMP-C1 (right image) cells compared to unconjugated/rhodamine B-labeled nanoliposomes (RhB-NxPs) (left image). Blue areas: anti-mPSCA-conjugated or -unconjugated NxPs. Red areas: isotype controls. The experiments were performed in triplicate. **c** Ultrastructural features of IL30-TRAMP-C1 cells untreated (a) or treated with Cas9mIL30 NxPs (b, c) or Cas9mIL30-mPSCA NxPs (d, e). The nanoparticles appear spherical and have a regular, submicron size (b–e, arrows). After 2 hrs of treatment, the unconjugated NxPs largely remained on the tumor cell surface (b, arrows), showing poor penetration into the cytoplasm (c, arrowhead). In contrast, the anti-mPSCA-conjugated NxPs were endocytosed by the tumor cell and were mostly found in the cell cytoplasm (d, e, arrowheads). M mitochondrion. N nucleus. PM plasma membrane. The values of the scale bars are reported in the images. **d** On-target and off-target characterization of CRISPR/Cas9gRNA-mediated *Il30* editing in vitro. Average frequency of CRISPR/Cas9gRNA-induced variants in the *Il30* gene (editing efficiency or on-target effects, OnT) and in off-target sites corresponding to recognized genetic loci (off-target effects, OTs) in IL30-TRAMP-C1 cell cultures treated with Cas9mIL30-PSCA NxPs or Cas9mIL30-NxPs (total editing efficiency of 82% and 12%, respectively). The frequency of variants (OnT and OTs) in cells treated with empty-mPSCA NxPs or PBS (controls) was <0.1%. The experiments were performed in triplicate. **e** On-target and off-target characterization of CRISPR/Cas9gRNA-mediated *Il30* editing in vivo. Average frequency of CRISPR/Cas9gRNA-induced variants in the *Il30* gene (editing efficiency or on-target effects, OnT) and in off-target sites (OTs) in the indicated organs of IL30-TRAMP-C1 tumor-bearing C57BL/6J mice treated with Cas9mIL30-mPSCA NxPs. The frequency of variants (OnT and OTs) in the organs of mice treated with empty-mPSCA NxPs or PBS (controls) was <0.1%. **f** Sequencing of the sgRNA-targeted *Il30* locus and mutation spectrum analysis using ICE. The image shows the percentage of frameshift or 21+ bp indels identified in IL30-TRAMP-C1 cells treated with Cas9mIL30-mPSCA NxPs or Empty-mPSCA NxPs. The data obtained from PBS-treated cells were comparable to those obtained from cells treated with empty-mPSCA NxPs. **g** Pharmacokinetics of free Cas9 and Cas9mIL30 NxPs conjugated or not conjugated with anti-mPSCA Abs in IL30-TRAMP-C1 tumor-bearing C57BL/6 J mice. %ID/g = percentage of total injection dose per weight. ANOVA, *p* < 0.001. *p < 0.01, Tukey HSD test *versus* free Cas9. **h** TEM analyses of syngeneic tumors from C57BL/6J mice treated with Cas9mIL30-mPSCA NxPs revealed spherical vesicles that were consistent with those of nanoparticles and close to (arrows) or inside (arrowheads) the tumor cells. N nucleus. PM plasma membrane. Scale bars: 2 μm. **i** The mean volume of wild-type and IL30-TRAMP-C1 subcutaneous tumors developed in C57BL/6J mice after treatment with Cas9mIL30-mPSCA, empty-mPSCA NxPs or PBS. ANOVA: *p* < 0.0001. *p < 0.01, Tukey HSD test *versus* IL30-TRAMP-C1 tumors treated with PBS or empty-mPSCA NxPs and PBS-treated wild-type TRAMP-C1 tumors. **p < 0.01, Tukey HSD test *versus* IL30-TRAMP-C1 tumors treated with PBS or Empty-mPSCA NxPs. The results are expressed as the mean ± SD. **j** Incidence of lung metastases in C57BL/6J mice bearing subcutaneous IL30-TRAMP-C1 tumors treated with Cas9mIL30-mPSCA, empty-mPSCA NxPs, or wild-type TRAMP-C1 treated with PBS. *Fisher’s exact test, *p* = 0.0005 *versus* IL30-TRAMP-C1 + empty-mPSCA NxPs. **k** Kaplan–Meier survival curves of 4 groups of fifteen C57BL/6J male mice bearing tumors developed after subcutaneous implantation of IL30-TRAMP-C1 cells or wild-type TRAMP-C1 cells treated with empty-mPSCA NxPs, Cas9mIL30-mPSCA NxPs or PBS. Mice bearing IL30-TRAMP-C1 tumors treated with Cas9mIL30-mPSCA NxPs survived longer (92 days) than mice bearing IL30-TRAMP-C1 tumors treated with PBS or empty-mPSCA NxPs (64 days) or mice bearing wild-type TRAMP-C1 tumors treated with PBS (78 days) (log-rank test: *p* < 0.000001). The mice were sacrificed when the tumors reached 2 cm^3^ in size. **l** TEM images of kidneys (a) and liver (b) from IL30-TRAMP-C1 tumor-bearing C57BL/6J mice treated with Cas9mIL30-mPSCA NxPs showing no signs of ultrastructural cell damage. N: nucleus. M: mitochondrion. Scale bars: 5 μm. **m** Subcutaneously developed IL30-TRAMP-C1 tumors from Empty-mPSCA NxP-treated animals (top panel) show a solid growth pattern with no signs of vascular damage (a), rich vascularization (b) and strong IL30 expression (c). In contrast, tumors from Cas9mIL30-mPSCA NxP-treated animals (bottom panel) showed multiple areas of vascular leakage (d, arrows) and hemorrhagic foci (arrowheads), which were associated with defective vascularization (e) and low to absent IL30 expression (f). Magnification a, c, d, f: 400×. Magnification b, e: 630×. Scale bars: a, c d, f: 25 µm; b, e: 10 µm. **n** Subcutaneously developed IL30-TRAMP-C1 tumors from Empty-mPSCA NxP-treated animals (top panel) showed considerable Foxp3^+^Treg lymphocyte (a) and NKp46^+^RORγt^+^ ILC3 (b) infiltration and high cytoplasmic and nuclear NFKB1 expression (c). Tumors from Cas9mIL30-mPSCA NxP-treated animals (bottom panel) showed few Foxp3^+^ Treg lymphocytes (d) and NKp46^+^RORγt^+^ ILC3 (e), and lower cancer cell expression of NFKB1 (f) compared to tumors from empty-mPSCA NxP-treated animals (c). Magnification: 400×. Scale bars: 25 µm.

Given that IL30-TRAMP-C1 and control tumors express PSCA (Fig. 8a), nanoparticles synthesized to treat murine IL30-producing tumors were functionalized, after loading with the Cas9gRNA-mIL30 complex (hereinafter referred to as the Cas9mIL30 complex), with anti-mouse (m) PSCA Abs (hereinafter referred to as Cas9mIL30-mPSCA NxP), as above reported for treating human IL30-producing PC xenografts. These NxPs specifically bound to mPSCA expressed by tumor cells (Fig. 8b). The liposomal loading capacity of the Cas9mIL30 complex was 63%. Both empty-mPSCA NxPs and Cas9mIL30-mPSCA NxPs were submicron (Supplementary Table 11, Fig. 8c) round nanostructures that were uptaken by tumor cells in vitro more efficiently than unconjugated NxPs (Fig. 8c).

Assessment of cleavage specificity for the selected sgRNA in IL30-TRAMP-C1 cell cultures and in C57BL/6J mice treated with Cas9mIL30-mPSCA NxPs (10 mg/ml, 48 h) or with PBS demonstrated the specificity of genome editing given that the frequency of variants of the off-target regions in cell cultures (Fig. 8d) and in mice (Fig. 8e) treated with Cas9mIL30-mPSCA NxPs was comparable to that observed in cells or mice treated with PBS (<0.1%).

Genome editing was assessed by ICE (Inference of CRISPR Edits), which can significantly expedite CRISPR editing workflows^19^. The findings revealed that the total editing efficiency of the targeted *mIl30* gene was 82% (Fig. 8f). The results of deep sequencing of the *Il30* gene region in IL30-TRAMP-C1 tumor tissues are consistent with these data because the total editing efficiency of the targeted *mIl30* gene reached 76% (Fig. 8e). The mutation pattern introduced by CRISPR/Cas9 editing in *mIl30* gene is described in the Supplementary Fig. 8. The serum and pH stabilities of the Cas9mIL30-mPSCA NxPs were comparable to those of the Cas9hIL30-hPSCA NxPs, and the release profile of Cas9 by Cas9mIL30 NxPs was comparable to that of Cas9hIL30 NxPs.

Importantly, the immunoliposomes used to target TRAMP-derived cancer cells were demonstrated to be nontoxic in vitro given that the viabilities of IL30-TRAMP-C1 cells treated with Empty-mPSCA NxPs or PBS (or untreated) were comparable (ANOVA: *p* > 0.05) (Supplementary Fig. 17). The pharmacokinetics of Cas9mIL30-mPSCA NxPs in IL30-TRAMP-C1 tumor-bearing C57BL/6J mice were comparable to those of Cas9hIL30-hPSCA NxPs in DU145 tumor- and PC3 tumor-bearing mice (Fig. 8g).

### Cas9gRNA-mIL30 targeting of IL30-overexpressing PCs in immunocompetent hosts decreases the recruitment of myeloid-derived cells, Tregs and ILC3s, inhibits NFKB1 expression, and hinders tumor progression

The effects of immunoliposomes targeting tumor-produced murine IL30, in a fully immunocompetent host, were tested by applying the conditions and schedule used for the treatment of PC xenografts (Supplementary Fig. 3). TEM images demonstrated that NxPs functionalized with anti-mPSCA Abs reached the tumor site as early as 30 min after i.v. administration and were efficiently uptaken and internalized by neoplastic cells (Fig. 8h).

NxP uptake by PC xenograft was also investigated by LSC microscopy. As in NSG mice bearing DU145 and PC3 tumors, a faint RhB signal was detected in tumors as early as 5 min post NxP inoculation, and its intensity progressively increased and showed a substantial difference between RhB-NxP and RhB-mPSCA-NxP treated tumors, which generated a stronger signal, starting 30 min after injection and peaking 2 hrs 30 min later (Supplementary Fig. 18).

The effect of Cas9mIL30-mPSCA NxPs on tumor production of IL30, as well as on the tumor microenvironment and biological behavior, was assessed. This experiment included one group of mice implanted with wild-type TRAMP-C1 cells and then treated with PBS in addition to three groups of C57BL/6J mice implanted with IL30-TRAMP-C1 cells and then treated with Cas9mIL30-mPSCA NxPs, empty-mPSCA NxPs, or PBS.

In mice bearing IL30-overproducing PCs, treatment with IL30-targeting immunoliposomes consistently slowed tumor growth compared to that in mice bearing IL-30-TRAMP-C1 tumors, treated with empty-mPSCA NxPs or PBS, and mice bearing wild-type TRAMP-C1 tumors treated with PBS (ANOVA: *p* < 0.0001) (Fig. 8i). At the end of the experiment, all the mice (15/15) bearing IL30-TRAMP-C1 tumors and treated with Empty-mPSCA NxPs or PBS had metastases, in contrast, 47% (7/15) of the mice bearing wild-type TRAMP-C1 tumors and treated with PBS developed lung metastases, and only 40% (6/15) of the mice bearing IL30-TRAMP-C1 tumors and treated with Cas9mIL30-mPSCA NxPs developed metastases (Fig. 8j). Kaplan–Meier analyses revealed that mice bearing IL30-TRAMP-C1 tumors and treated with Cas9mIL30-mPSCA NxPs survived longer (92 days) than controls (PBS- and empty-mPSCA-treated) (64 days) and PBS-treated TRAMP-C1 tumor-bearing mice (78 days) (log-rank test: *p* < 0.000001) (Fig. 8k). The organs from the Cas9mIL30-mPSCA NxP- or empty-mPSCA NxP-treated animals were histologically normal (Supplementary Fig. 19) and free of ultrastructural cell damage (Fig. 8l). Hematological analysis confirmed the absence of specific organ toxicity (Supplementary Table 12). Assessment of NxP immunogenicity in fully immunocompetent C57BL/6J mice demonstrated that i.v. inoculation of naked Cas9gRNA-mIL30 complex, empty-mPSCA NxPs, or Cas9mIL30-mPSCA NxPs induced a minimal increase in the blood levels of TNFα and IL6, which returned to baseline within 24 hrs.

Treatment with Cas9mIL30-mPSCA NxPs suppressed IL30 expression and resulted in multiple foci of necrobiosis and ischemia associated with impaired microvessel density (MVD) and reduced cancer cell proliferation (ANOVA: *p* < 0.0001) compared with those in tumors treated with empty-mPSCA NxPs or PBS (Fig. 8m and Supplementary Table 13). Immunopathological studies demonstrated that treatment with Cas9mIL30-mPSCA NxPs prevented the intratumoral recruitment of Foxp3^+^ regulatory T lymphocytes (Tregs) (Fig. 8n) and nonspecific immune cells, such as F4/80^+^ macrophages, CD11b^+^Gr-1^+^ myeloid-derived cells (MDCs) and Ly-6G^+^ granulocytes, which characterize the IL30-TRAMP-C1 tumor microenvironment (TME). In contrast, CD3^+^CD8^+^ and CD3^+^CD4^+^ T lymphocyte levels were comparable to those observed in empty-mPSCA NxP-treated tumors or PBS-treated IL30-overexpressing and wild-type tumors (automated immune cell quantitation in the TME is reported in Supplementary Fig. 20). Furthermore, NKp46^+^RORγt^+^ cells, which are known as innate lymphoid cells 3 (ILC3), heavily infiltrated untreated and empty-mPSCA NxP- or PBS-treated IL30-TRAMP-C1 tumors, as observed in IL30-overexpressing syngeneic breast cancers^11^, whereas their number was consistently reduced in Cas9mIL30-mPSCA NxP-treated IL30-TRAMP-C1 tumors (Fig. 8n, Supplementary Fig. 20). The reduced intratumoral immune cell infiltration due to IL30-targeting treatment was accompanied by substantial inhibition in cancer cells expression of NFKB1 (Fig. 8n), which is a key player in cancer-immune cell crosstalk and in modulating antitumor immunity^28^.

## Discussion

Immunotherapy has been a breakthrough in the treatment of advanced tumors^29^, but limited results have been obtained in PC. The identification of new targetable immunosuppressive oncogenes would be useful.

IL30 has demonstrated “*prostate cancer driving*” properties. Indeed, the IL30/IL6R/gp130 axis promotes inflammatory, immunosuppressive and cancer progression programs and is critical for PC onset and metastasis^7,9,12,30^. Given that PC mainly affects the fragile and progressively expanding elderly population, a tumor-selective and well-tolerated therapeutic approach is especially needed. Nanobiotechnology can provide innovative tools for the development of precision cancer immunotherapy, and IL30 represents a potential therapeutic target in PC.

In this study, we used a microfluidic platform and EMA- and FDA-approved biocompatible components suitable for clinical use to produce sterically stable submicron particles consisting of self-assembled lipids and an anionic ribonucleoprotein complex formed by Cas9 and sgRNA^31^ to knock out the *IL30* gene.

The encapsulation efficiency, which was not particularly high due to the large size of the loaded protein^32^, nevertheless ensures a good level of genome editing and efficient deletion of the *IL30* gene, without off-target effects both in vitro and in vivo. PEGylation prevents nanoliposome opsonization and clearance and allows the nanoparticles to retain their size and integrity under a wide range of pH conditions^33,34^; therefore, PEGylation provides high serum stability and a sufficiently long circulation time after intravenous injection, suggesting resistance to macrophage uptake and enzymatic degradation^35^. Moreover, the lipid formulation we used, including DOTAP, DOPE, and cholesterol, was selected to promote both bilayer stability, which ensures the structural integrity of liposomes, and endolysosomal escape^36^. Nanoparticle functionalization with Abs^37^ that specifically recognize and bind to PSCA, which is overexpressed by most PCs^20^, ensures efficient site-specific accumulation and favors internalization into PC cells, as revealed by ultrastructural studies.

Our nanocarriers are ineffective on cancer cell viability. When loaded with the Cas9gRNA-IL30 complex and conjugated with antitumor-specific Abs, that promote their uptake by PC cells, nanoparticles exert antiproliferative effects on both cell cultures and palpable tumors. Histopathological analysis of the liver, kidneys, spleen, heart, lungs, and prostate of immunoliposome-treated mice, together with the ultrastructural study of the liver and kidneys, the organs primarily involved in lipid metabolism^22^, excluded the possibility of organ damage or obvious inflammatory responses. These data were substantiated by the lack of significant changes in weight and serum levels of inflammatory cytokines and markers of cardiac, kidney, and liver function, confirming the absence of side effects.

Chronic biweekly treatment with immunoliposomes, starting at tumor onset, substantially inhibited tumor growth and progression. After traveling through the pulmonary circulation, intravenously administered immunoliposomes enter the systemic circulation but exclusively accumulate in PSCA^+^ tumors, yielding inhibitory effects on tumor progression programs.

In the PC3 model, the antitumor effect of the treatment was comparable to that obtained from knocking out the *IL30* gene in cancer cells using classical CRISPR/Cas9-mediated gene editing. In the DU145 tumor model, the efficacy of the nanotherapy in slowing tumor growth and improving survival was lower than what was observed when the *IL30* gene was deleted in cancer cells before xenograft implantation, however, the antimetastatic effect was comparable to that of DU145 IL30KO tumors of the same size and resulted in more than half of the animals being free from lung metastases. In contrast, all control mice developed lung metastasis. These results corroborate data on the antimetastatic potential of targeting IL30^9,12,30^.

Notably, in both the AR^+^ (DU145) and AR^-^ (PC3) IL30-expressing tumor models, treatment with CRISPR/Cas9gRNA-hIL30-loaded immunoliposomes substantially improved survival. Large areas of ischemic necrosis associated with severely compromised vascularization, due to the overwhelming downmodulation of proangiogenic genes compared to antiangiogenic genes, which reprogram PC-endothelium crosstalk^38^, and reduced tumor cell proliferation are the hallmarks of nanotherapy-induced tumor growth inhibition. The extensive remodeling of the gene expression signature of PC cells include the suppression of a broad range of angiogenic and angiocrine factors, such as *IGF1*^39^*, CCN2*^40^*, CXCL5, CXCL8*^41^*, EDN1*^42^*, ITGAV*^43^*, TNF*^44^*, MMP14*^45^*, FGF1,* and *FGF2*^46^, associated with the downregulation of oncogenes, such as *PTGS2*^47^, and a key driver of inflammation and PC cell survival, namely, *NFKB1*^24^, and the upregulation of tumor suppressors, such as *CDH1*^48^*, DKK3*^49,50^, and *PTEN*^51^. These pathways are among the molecular mechanisms triggered by both classical and nanotherapy-mediated *IL30* genome editing, which underlies its antitumor efficacy. These mechanisms are also largely involved in the response to treatment with the IL30-targeting immunoliposome in the syngeneic model of IL30-overexpressing PC in fully immunocompetent hosts, of which the inhibition of angiogenesis and proliferation and the downmodulation of NFKB1^28^ are hallmarks. In addition, in the syngeneic PC model, treatment with Cas9gRNA-mIL30-loaded nanoparticles prevents intratumoral infiltration of macrophages, MDCs and granulocytes and inhibits Treg^52^ and ILC3^53–55^ influx, thus abrogating the immune cell landscape orchestrated by IL30 overproducing tumor^12^.

Remodeling the TME in immunocompetent hosts is associated with substantial inhibition of tumor growth and metastasis, leading to improved survival of *IL30-*targeting immunoliposome-treated mice and strengthening the results obtained in xenograft tumor models.

Notably, human IL30 is membrane-anchored, whereas its murine counterpart,^12^ with which it shares 73% homology, is a secreted cytokine. However, the consequences of *IL30* gene editing on xenograft and syngeneic tumor behavior largely overlap. As observed for many antiangiogenic drugs, particularly those targeting the VEGF/VEGFR pathway^56,57^, the occurrence of adaptive resistance and tumor regrowth due to the selective pressure exerted by an IL30-targeting therapy cannot be excluded. Although the molecular mechanisms that may cause treatment failure remain to be investigated, the emergence of treatment resistance and resumption of tumor progression could be limited by combining IL30 suppression with immunotherapy or other personalized treatments, as observed in the combined treatment protocols that have been successfully used for the management of different types of advanced stage cancer^58,59^.

To date, different liposome-based formulations are approved for clinical use in the treatment of various advanced stage tumors^60,61^. However, immunoliposomes that selectively target tumors and deliver a CRISPR/Cas9 complex to edit tumor-promoting and immunoregulatory genes are absent from the current therapeutic landscape.

By rescuing the TME from immunosuppression, IL30-targeting nanotherapy exhibits the potential to overcome resistance to T-cell-based immunotherapies^61^, which have thus far been unsuccessful in the treatment of PC^62,63^. Immunocompetent models of PC may provide insight into whether targeting IL30 in the context of combination therapy may reverse the disappointing results (presented at the ESMO Congress 2023) achieved in the latest clinical trials with Sipuleucel-T (Provenge®), the autologous dendritic cell vaccine, or with Pembrolizumab, the humanized anti-PD-1 monoclonal Ab, both of which are FDA-approved for the treatment of metastatic castration-resistant PC (https://dailyreporter.esmo.org/esmo-congress-2023/genitourinary-cancers).

Immunopathological and clinical data show that among patients who underwent prostatectomy due to high-grade and locally advanced PC, those with IL30^negative^ cancer had a better tumor immune response and a longer disease-free survival than patients with IL30^positive^ cancer^43^, emphasizing the clinical value of an IL30-targeting therapeutic strategy.

The dramatic disruption of proangiogenic and tumor progression programs and the overcoming the immunosuppressive TME, attributed to the selective nanoliposome-mediated deletion of the *IL30* gene in cancer cells, paves the way for the development of a modern and safe immuno-nanotherapy for advanced PC that meets the needs of healthy aging.

## Supplementary information

SUPPLEMENTARY MATERIAL

## Data Availability

The data generated in this study are available upon request from the corresponding author.
