# Supplementary material for: Immunoliposome-based targeted delivery of the CRISPR/Cas9gRNA-IL30 complex inhibits prostate cancer and prolongs survival
Source: Exp Mol Med. 2024 Sep 4;56(9):2033–51. doi: 10.1038/s12276-024-01310-2 (PMC11447253; doi:10.1038/s12276-024-01310-2)
Supplement: Supplementary file 1 — SUPPLEMENTARY MATERIAL [file 12276_2024_1310_MOESM1_ESM.pdf]

SUPPLEMENTARY MATERIALS AND METHODS

Flow cytometry and antibody conjugation efficiency

To assess PSCA expression, DU145, PC3, IL30-TRAMP-C1 and TRAMP-C1 cells were harvested and mechanically dissociated into a single cell suspension. The cells were pelleted and resuspended in PBS with 0,5% BSA and incubated for 30mins, at 4 °C, with anti-PSCA Ab (Thermo Fisher Scientific Cat# PA5-65080, RRID:AB_2662130, for human PC cells and Creative Biolabs [Shirley, NY, USA], HPAB-1900-FY-S(P), for murine PC cells). Subsequently, cells were pelleted and washed in PBS, and incubated for 30mins, in at 4 °C, with Alexa fluor 488 secondary Ab (Thermo Fisher Scientific Cat# A-11008, RRID:AB_143165). Acquisition was performed using a BD Scientific Canto II Flow Cytometer (RRID:SCR_018056), and the data were analyzed using FlowJo software (RRID:SCR_008520). Dead cells were excluded by 7AAD staining.

Conjugation efficiency, between the anti-PSCA Ab and the Aldheyde-modified DSPE-PEG2000 lipid present on the external layer of the NxP, was assessed by flow cytometry. Briefly, $1x10^6$ PC cells were seeded in T25 cell culture flasks and, after 24h, they were incubated for 1h with Rhodamine labelled NxPs, conjugated or not with anti-PSCA Abs. Cells were harvested and mechanically dissociated into a single cell suspension. The cells were then pelleted by centrifugation at 230g, washed in PBS, and analysed as described above.

Synthesis of nanoliposomes and functionalization with anti-PSCA Abs

Nanoliposomes (NxPs) were synthesized by using Dolomite Microfluidics (Royston, UK), which enables standardized production of NxPs (low polydispersion index, in the range of 0.06-0.25), high encapsulation efficiency and overcomes the need for high temperatures during component assembly, which have proven detrimental to the stability of the Cas9/gRNA complex. Lipids used to produce the NxPs are as follows.

- 1,2-Dioleoyl-3-trimethylammonium-propane (DOTAP, Avanti Polar Lipids, Alabaster, AL, USA), a cationic lipid used to obtain optimal loading efficiency of the Cas9/gRNA complex, which is negatively charged,[1,2] and which also function as PEG linker.
- Fusogenic lipids, specifically, 1,2-dioleoyl-sn-glycero-3-phosphoethanolamine (DOPE, Avanti Polar Lipids, Inc.), useful to foster nanoparticle entry into the targeted cells.[3]
- Stealth lipids, specifically, 1,2-distearoyl-sn-glycero-3-phosphoethanolamine-N-[amino(polyethylene glycol)-2000] (DSPE-PEG2000, Avanti Polar Lipids), used to increase circulation time and to prevent opsonization and subsequent phagocytosis by macrophages.[4]
- DSPE-PEG2000, Aldheyde/Maleimide modified PEG lipids, used for Ab conjugation.[5]
- DOTAP, DOPE and DSPE-PEG2000 were purchased from Avanti Polar Lipids.
- Cholesterol (Sigma-Aldrich Co. St. Louis, MO) was used to provide structural stability to the lipid bilayer.[6]

To produce Empty NxPs, lipids (DOPE, DOTAP, CHOL, DSPEpeg2000, DSPE-PEG-CHO) (Avanti Polar Lipids) were dissolved in ethanol (at molar ratio of 3.45:3.45:2.1:0.75:0.25, respectively) and then mixed, in a micromixer chip, with PBS, at a 1:3 flow rate ratio (lipids:PBS). Then, nanoliposomes were dialyzed with Slide-A-Lyzer Dialysis Cassettes, 20K MWCO (#66003, ThermoFisher Scientific), against PBS, to remove ethanol. Lastly the nanoparticle suspension was concentrated with Pierce™ Protein Concentrator PES, 100K MWCO (#88523X4, ThermoFisher Scientific) in order to reach a final lipid concentration of 10 mg/ml.

To prepare Rhodamine (Rh) labeled nanoliposomes, Lissamine™ Rhodamine B 1,2-Dihexadecanoyl-sn-Glycero-3-Phosphoethanolamine, Triethylammonium Salt (#L1392, ThermoFisher Scientific) was added to the lipid mixture, at a 0.1% molar ratio.

To produce core-encapsulated (CE) gadolinium (Gd) nanoliposomes, CE-Gd-NxPs, lipids were dissolved in ethanol and then mixed, in a micromixer chip, with a Gadoteric acid (Gd-

DOTA; #G360000, SimSon Pharma, Mumbai, India) solution at a 1:3 flow rate ratio (lipids:Gd-DOTA). After mixing, unencapsulated Gd was removed by dialysis with Slide-A-Lyzer Dialysis Cassettes, 20K MWCO (ThermoFisher Scientific). Then, the nanoparticle suspension was concentrated with Pierce™ Protein Concentrator PES, 100K MWCO (ThermoFisher Scientific) in order to reach a final lipid concentration of 10 mg/ml. Lastly, nanoliposomes were conjugated with anti-PSCA Abs (#PA5-65080, ThermoFisher Scientific) and dialyzed with a 300k MWCO membrane (#131456T, ThermoFisher Scientific) to remove unbound Abs.

To produce Cas9IL30 NxPs, lipids were dissolved in ethanol and then mixed, in a micromixer chip, with Cas9gRNA-IL30 complex solution, at a 1:3 flow rate ratio (lipids:Cas9gRNA-IL30). Then, the liposomes were dialyzed against PBS, at a pH of 6.5, with a 300k MWCO membrane (ThermoFisher Scientific) to remove unencapsulated Cas9gRNA-IL30 complex. The nanoparticle suspension was concentrated with Pierce™ Protein Concentrator PES, 100K MWCO (ThermoFisher Scientific), to reach a final lipid concentration of 10 mg/ml.

Subsequently, anti-PSCA specific Abs (ThermoFisher Scientific) were conjugated on the PEG-CHO derivatives (Avanti Polar Lipids) of the external bilayer of the pre-formed nanoliposomes, to obtain Cas9gRNA-hIL30-hPSCA NxP, referred to as Cas9IL30-PSCA NxP, using a displacement reaction mediated by sodium cyanoborohydride (#156159, Merck, Darmstadt, Germany). The nanoliposomes were dialyzed against PBS, at a pH of 7.4, with a 300k MWCO membrane (ThermoFisher Scientific) to remove unbound Abs and excess of sodium cyanoborohydride.

Physical characterization of nanoliposomes

The nanoparticles were characterized as follows.

- *Laser particle size analysis and zeta electromotive force analysis*

To determine particle size and zeta electromotive force, Empty NxPs and Cas9gRNA-IL30 NxPs were dispersed in double-distilled water (final volume of 2 ml) and particle size and zeta electromotive force were measured by using a 90Plus/BI-MAS ZetaPlus multiangle particle size analyzer (Brookhaven Instruments, Holtsville, NY, USA).

- *Serum stability of the Cas9gRNA-IL30 NxPs*

To determine the serum stability of Cas9gRNA-IL30 NxPs, they were resuspended in a medium containing 10% fetal calf serum, and the size of the nanoliposomes was measured every 6 hours, up to 24 hours by using a 90Plus/BI-MAS ZetaPlus multiangle particle size analyzer (Brookhaven Instruments).

- *Stability of Cas9gRNA-IL30 NxPs to different external pH values*

The Cas9gRNA-IL30 NxPs were resuspended in a medium containing 50% fetal calf serum at pH 2.5, pH 6.5, or pH 9, followed by vortex blending. The resuspended solution was placed on a horizontal shaker (70 rpm, 37 ± 1 °C) and, at fixed time intervals (0, 2, 4, 6, 12 and 24 hours), the particle size of the nanoliposome was measured by using a 90Plus/BI-MAS ZetaPlus multiangle particle size analyzer (Brookhaven Instruments).

- *Morphological analyses of Cas9gRNA-IL30 NxPs by transmission electron microscopy (TEM)*

Size and shape of nanoparticles were characterized by TEM. Empty nanoliposomes conjugated with anti-PSCA Abs (Empty-PSCA NxPs) and Cas9gRNA-IL30 NxPs, conjugated with anti-PSCA Abs (Cas9IL30-PSCA NxPs) were incubated in fresh culture medium, at 1:25, 1:50 and 1:100 dilutions, for 3h. Then, the NxPs were fixed in cacodylate-buffered 2.5% glutaraldehyde, post-fixed in osmium tetroxide, and embedded in Epon 812. Ultrathin sections were stained with uranyl acetate–lead citrate and analyzed with a Philips

CM10 and a Fei-Philips Morgagni 268D transmission electron microscope (Philips, Eindhoven, NL).

Encapsulation efficiency and release rate of the Cas9IL30 NxP

The Cas9IL30 NxP was cleaved in dimethyl sulfoxide (DMSO) and diluted in PBS (pH 7.4). The resulting mixture was then centrifuged in a high-speed refrigerated centrifuge at 15,000 rpm for 1h, and Cas9 quantification was performed by ELISA assay (Cas9 ELISA Kit, # PRB-5079, Cell Biolabs, San Diego, CA, USA) on the supernatant. Blank control was prepared by the same method, using the supernatant from the Empty nanoliposomes. The encapsulation efficiency was calculated according to the following formula: Encapsulation rate (%) = [(A2-A1)/A2] × 100%, where A1 was the amount of Cas9 measured in the supernatant and A2 was the initial amount of Cas9 used to prepare the Cas9IL30 NxP.

To assess the release rate of the Cas9IL30 NxP the Cas9IL30 NxP was cleaved in DMSO, suspended in 1 ml of PBS (pH 7.4), and incubated at 37°C with gentle shaking. Subsequently, at scheduled time points (5, 10, 20, 30 minutes, and 1, 2, 3, 4, 5, 6, 7, 8, 10, 12, 24, 36 hours), the suspension was centrifuged at 15,000 rpm and an aliquot of the supernatant was taken for Cas9 quantification (Cas9 ELISA Kit, Cell Biolabs) and replaced with an equal volume of PBS, to continue monitoring of the release rate of NxPs.

Editing quantification and analysis of the off-target cleavage *in vitro* and *in vivo*

For editing quantification and evaluation of off-target cleavage events for the selected sgRNAs (identified using COSMID software, https://crispr.bme.gatech.edu, and CRISPR-Cas9 guide RNA design checker, https://eu.idtdna.com/site/order/designtool/index/CRISPR_SEQUENCE). The whole genome obtained from human and murine PC cell cultures, treated or not with Cas9IL30-PSCA NxPs, and from tumor, prostate, lungs, heart, liver, kidneys, prostate and spleen of

three PC3, DU145, or IL30-TRAMP-C1 tumor bearing mice, treated with one injection of Cas9IL30-PSCA NxPs, and six untreated mice (three NSG and three C57BL/6J, controls), was sequenced by Lexogen GmbH (Wien, Austria), using the Illumina Platform NovaSeq 6000 System and Dragen software. The reads were aligned to the reference genomes and the percentage of sequence reads with insertions or deletions (variants) over the total number of sequence reads were calculated. The average frequency of variants induced in the *IL30* gene represented the On-Target effects (editing efficiency), whereas the average frequency of variants induced in Off-Target sites, represented the Off-Target effects.

To evaluate cumulative editing, three groups of 3 NSG mice bearing PC3 tumors or DU145 tumors, and three groups of 3 C57BL/6J mice bearing IL30-TRAMP-C1 tumors were euthanized after 2, 4 or 6 NxPs injections, DNA was extracted from tumors and the editing efficiency was evaluated as described above.

Efficiency of genome editing in Cas9IL30-PSCA NxP treated human and murine PC cells by Tracking of Indels by Decomposition Analysis or Inference of CRISPR Edits

CRISPR/Cas9 induced insertions/deletions (indels) frequencies, in DU145 and PC3 cells, were quantified using Tracking of Indels by decomposition (TIDE) method. Briefly, genomic DNA was isolated from DU145 and PC3 cells, treated, for 48 hours, with Cas9IL30-PSCA NxPs or Empty-PSCA NxPs (controls), at concentration of 1mg/ml, using QIAamp DNA Mini Kit (#51304, Qiagen, Hilden, Germany) and the DNA stretch enclosing the designed editing site was amplified by PCR. Next, the PCR amplicons were subjected to conventional Sanger sequencing by Eurofins Genomics (Ebersberg, Germany) and the resulting sequence trace files were analyzed using the TIDE web tool (available at http://tide.nki.nl). To evaluate the allelic distribution of the CRISPR/Cas9 induced mutations, DU145 and PC3 cell populations, treated with Cas9IL30-PSCA NxPs, were subcloned in single-cell-derived clones, whose

DNA was extracted and subjected to conventional Sanger sequencing of the editing site by Eurofins Genomics (Ebersberg, Germany).

To analyse the efficiency of the CRISPR/Cas9 system in deleting the *Il30* gene in IL30-TRAMP-C1 cells, the DNA was extracted from cells treated with Cas9mIL30-PSCA NxPs, Empty-PSCA NxPs or PBS, and used as a template to amplify the sgRNA target region, using a standard PCR program. The PCR products were subjected to conventional Sanger sequencing by Eurofins Genomics (Ebersberg, Germany). The sequencing data was subsequently analyzed by ICE (Synthego, https://ice.synthego.com), to quantify the percentage of frameshift or 21+ bp indels, which result in a functional Knockout (KO) of the targeted gene.

Ultrastructural analyses of human and murine PC cells treated with Cas9IL30-PSCA NxP

NxP uptake by PC cells *in vitro* was investigated by transmission electron microscopy (TEM). One million of DU145, or PC3, or IL30-TRAMP-C1 cells were seeded in T25 cell culture flasks and were untreated or treated with a 1:25 dilution of Cas9IL30 NxPs, or Cas9IL30-PSCA NxPs, for 1h, 2h or 3h in fresh culture medium. Then, PC cells were fixed in 2.5% glutaraldehyde, post-fixed in osmium tetroxide, and embedded in Epon 812. Ultrathin sections were stained with uranyl acetate–lead citrate and analyzed with a Philips CM10 and a Fei-Philips Morgagni 268D transmission electron microscope (Philips, Eindhoven, NL).

Biosafety and distribution of nanoliposomes *in vivo*

- *For measuring markers of organ toxicity in serum and assess haemolytic activity,* blood samples from mice treated with PBS, naked Cas9IL30 complex, Empty-PSCA NxPs, Cas9IL30 NxPs or Cas9IL30-PSCA NxPs were collected, 21 days after the start of treatment, from the venous sinus of the mice using a capillary tube.

Aspartate aminotransferase (AST), alanine aminotransferase (ALT), blood urea nitrogen (BUN), creatinine (Cr), cardiac troponin-1 (cTnI), creatine kinase (CK) and lactate dehydrogenase (LDH) were measured in the sera, 21 days after the start of treatment, by using Mouse AST ELISA Kit (ab263882), Mouse ALT ELISA Kit (ab282882), Creatinine Assay Kit (ab65340), Creatine Kinase Activity Assay Kit (ab155901) and LDH Assay Kit (ab102526) (all from Abcam, Cambridge, UK), Urea Nitrogen (BUN) Colorimetric Detection Kit (Thermo Fisher Scientific) and Mouse Troponin I ELISA Kit (Novus Biologicals, Centennial, CO, USA), according to the manufacturers' protocols.

- *For Hemolysis assay*, fresh blood samples were collected from 3 NSG mice and centrifuged at 500×g for 5mins. The plasma was aspirated and replaced with PBS at pH 7.4. Solutions with different concentrations (0.25, 0.5, 1.0 and 1.5 mg/ml) of NxPs were prepared and pipetted into a 96-well plate (each concentration was loaded in triplicates). Untreated blood sample was used as the negative control and Triton X-10% treated blood was used as the positive control. Diluted erythrocytes were pipetted to each well, plates were incubated at 37 °C for 1 h and, subsequently, centrifuged for 5mins at 500×g, to pellet intact erythrocytes. Using a multichannel pipet, 100 µl of supernatant was transferred from each well into a clear, flat-bottomed 96-well plate. Absorbance of supernatants was then measured, using a spectrophotometer, and the results were expressed as the mean percentage of haemolysis ± SD, compared to the positive control, for each concentration.
- *To assess the immunogenicity of NxPs,* four groups of five fully immunocompetent male BALB/c, or C57BL/6J mice were i.v. injected with PBS, or naked CRISPR/Cas9IL30 complex, or Empty-PSCA NxPs or Cas9IL30-PSCA NxPs. Six- and 24-hours post injection, blood samples were collected and processed to assess the serum levels of TNFα and IL6, by using the Mouse TNF-alpha Quantikine ELISA Kit (#MTA00B; R&D

Systems, Minneapolis, MN, USA) and the IL-6 Mouse ELISA Kit (#KMC0061; Thermo Fisher Scientific) according to the manufacturers' protocol.

- *Histopathological and ultrastructural analyses of the intracellular uptake and toxicity of NxP in vivo.*

  For histology, after autopsy of NxP treated and control mice, tissue samples from organs (tumor, lungs, heart, liver, kidneys, prostate and spleen) and tumors, were fixed in 4% formalin, embedded in paraffin, sectioned at 4 µm and stained with hematoxylin and eosin. For ultrastructural analyses with TEM, small tissue samples (1mm^3) from liver, kidneys and from the inner areas and edges of the tumour xenografts from NxP treated and control mice, were fixed in cacodylate-buffered 2.5% glutaraldehyde for 24 h at 4 °C, post-fixed in 1 % osmium tetroxide for 2h, dehydrated with graded acetones and embedded in Epon 812 (Electron Microscopy Sciences). Ultrathin sections (0.5-1 micron-thick) were stained with 1 % methylene blue, and used to select suitable areas of ultrastructural sectioning. Finally, ultrathin sections (60 nm-thick), were collected on copper grids, counterstained with uranyl-acetate and lead citrate and examined with a Philips CM10 (TEM) and/or Fei-Philips Morgagni 268D transmission electron microscopes (FEI, Eindhoven, The Netherlands).

- *Laser scanning confocal (LSC) microscopy analyses of the lung transit and tumor uptake of the NxPs.*

  For LSC microscopy analyses of the lung transit and tumor uptake of the NxPs, two groups of 24 NSG male mice were s.c. inoculated with GFP-labeled DU145 or PC3 cells. When the green-fluorescent tumors reached 0.5 cm^3, each group of mice was divided into two groups of 12 mice, one treated with rhodamine (Rh)-labeled NxPs (Rh-NxP) and the other with Rh-labeled NxPs conjugated with anti-PSCA Abs (Rh-PSCA-NxP). Each group of 12 mice was then divided into 4 groups of three mice, which were sacrificed at different time points (5 mins, 15 mins, 30 mins, 2 hrs and 30 mins) after i.v. administration

of NxPs, as suggested by the MRI captures. For the analyses of the lung transit, and tumor uptake of the NxPs, in TRAMP-C1 tumor-bearing mice, 24 C57BL/6J male mice were s.c. injected with $5x10^5$ GFP-labeled IL30-TRAMP-C1 cells. When the tumors reached 0.5 cm^3, each group of mice was divided into two groups of 12 mice, one treated with rhodamine (Rh)-labeled NxPs (Rh-NxP) and the other with Rh-labeled NxPs conjugated with anti-mPSCA Abs (Rh-mPSCA-NxP). Each group of 12 mice was then divided into 4 groups of three mice, which were sacrificed at different time points (5 mins, 15 mins, 30 mins, 2 hrs 30 mins) after i.v. administration of NxPs.

LSC images of tumors and lungs were captured under an LSM 800 confocal microscope (Zeiss, Oberkochen, Germany, RRID:SCR_015963).

To quantify the tumor uptake of the NxPs, the fluorescence intensity and distribution of Rh-NxPs, or Rh-PSCA-NxPs, and GFP^+ tumor cells were evaluated, using the ZEN Microscopy Software (Zeiss, RRID:SCR_013672), and the tumor uptake of the NxPs was expressed as the mean percentage ± SD of Rh^+GFP^+cells/total number of GFP^+cells. PBS-treated GFP^+ tumor-bearing mice were used as a negative control. The fluorescence intensity and distribution of Rh-NxPs, or Rh-PSCA-NxPs, in the lung parenchyma were visualized, by LSC analyses, after staining of the alveolar epithelial cells with rabbit anti-EpCam antibody (Thermo Fisher Scientific Cat# MA5-35283, RRID:AB_2849185), followed by goat anti-rabbit IgG Alexa-Fluor-488 conjugated antibody (Thermo Fisher Scientific Cat# A32731, RRID:AB_2633280). Three sections per sample were analyzed and six to eight high-power fields were evaluated for each section.

Magnetic Resonance Imaging (MRI)

To assess the biodistribution of NxPs and to determine their efficiency in selectively targeting the tumor site, two groups of nine eight-week-old NSG male mice were s.c. inoculated with $5x10^5$ wild-type DU145 cells, or with $1x10^6$ wild-type PC3 cells. Fifty-three and forty days later, i.e. when DU145 and PC3 tumors, respectively, reached a mean volume of 0.5 cm^3, each group of tumor-bearing mice was divided into three groups, which were i.v. inoculated with contrast agent alone (Gadoteric acid, Gd, 0.6 mmol/kg; GE Healthcare, Chicago, IL, USA), or CE-Gd-NxPs, or CE-Gd-NxP-hPSCA (10 ng/ml). Image acquisition and analysis was performed, using a 3T scanner (Philips Medical Systems Nederland).

Both T1- and T2-weighted images were obtained in tumor-bearing NSG mice, before and after NxPs administration, using a 3 T scanner (Philips Medical System, Best, the Netherlands), equipped with a sense flex surface coil. During image acquisition, mice were anesthetized with 50 mg/kg ketamine and 5 mg/kg xylazine and placed in the surface coil in the supine position. The animal's body temperature was maintained at 37°C by an automatic feed-back heating system throughout the period of MRI data acquisition.

Axial, coronal and sagittal images, covering the mouse from the head to tail, were acquired, using T2-weighted coronal fat-saturated fast spin-echo (FSE)(TR/TE 3000/30 ms), T1-weighted spoiled gradient-echo (SPGR) (TR/TE/FA 800/10.6ms/12°), and T2-weighted (TR/TE 4000/34 ms) fat-saturated FSE sequences, before and after i.v. injection of CE-Gd NxP, CE-Gd-PSCA NxP or control agent (Gadoteric acid, Gd-DOTA). The scanning time points for the T1-weighted images were 5 mins, 15 mins, 30 mins, 1 hr and 30 mins, 2 hrs and 30 mins, 4 hrs, 5 hrs and 30 mins after i.v. administration of NxPs. For quantitative analysis of T1-weighted MR images of tumors *versus* organs, the signal intensity is reported as dose in tissue sample/injected dose x 100 (percentage of the injected dose, %ID). The post-enhanced T2-weighted axial images were then acquired immediately after post-contrast T1-weighted image acquisition. All MR images were analyzed, on a Philips MR workstation, by two radiologists in a blind fashion.

Western blotting quantification of the tissue distribution of Cas9 protein

Tissue samples from tumor, prostate, lungs, liver, kidney and spleen, from NSG mice treated with Cas9IL30-PSCA NxPs or unconjugated NxPs, were homogenized at 4°C and crude homogenates were passed five times through a 22-G needle attached to a syringe and centrifuged for 5mins at 10,000g, 4°C. Total proteins were then quantified using the Bradford assay. Subsequently, lysates were loaded on Mini-PROTEAN TGX Gels 4-20% (#4561094; Bio-Rad, Hercules, CA, USA) and proteins were transferred from the gels on Immuno-Blot PVDF Membranes (#1620177; Bio-Rad, Hercules, CA, USA) in transfer buffer (glycine, tris [pH 8.4] and methanol) using Mini Trans-Blot Cell apparatus (Bio-Rad). Membranes containing the transferred proteins were then blocked with 5% milk (Sigma-Aldrich, St. Louis, MO, USA) in TBST and, subsequently, probed with primary and horseradish peroxidase conjugated secondary antibodies, following standard procedures. The following primary and horseradish peroxidase conjugated secondary antibodies were used: rabbit anti-Cas9 antibody (Thermo Fisher Scientific Cat# MA5-32636, RRID:AB_2809913) and mouse anti-β-Actin (Sigma-Aldrich Cat# A2228, RRID:AB_476697), as loading control, goat anti-rabbit IgG (H + L)-HRP Conjugate (Sigma-Aldrich Cat# A0545, RRID:AB_257896) and rabbit anti-mouse IgG (whole molecule)-Peroxidase (Sigma-Aldrich Cat# A9044, RRID:AB_258431). Membranes were then washed with TBST and developed with Pierce ECL WB Substrate (#32106; Thermo Fisher Scientific, Waltham, MA, USA). Signal intensities were analyzed by ImageJ software (National Institutes of Health, Bethesda, MD, USA) and normalized to β-actin expression.

Histology, immunohistochemistry and TUNEL assay

For histology, tissue samples were fixed in 4%-formalin, embedded in paraffin, sectioned at 4-μm and stained with H&E. Single or double (CD11b/Gr-1, and NKp46/RORγt) immunostainings, on formalin fixed and paraffin-embedded, or frozen, tissue sections, were-performed as described,[7,8] and using the antibodies listed in the Supplementary Table 3.

To eliminate inter-operator bias and to improve data reproducibility, assessment of the proliferation and apoptotic index, of the microvessel count and of the expression of angiogenic and cancer driver genes in tumor samples was performed by light microscopy, at ×400 in an 85,431.59 μm^2 field, on single immunostained sections, with a Leica Imaging Workstation and QWin image analysis software (Leica QWin, RRID:SCR_018940), which ensures the following highly reproducible steps: 1) image acquisition; 2) conversion of RGB image (true colors) to binary image (black and white); 3) filtering to remove noise; 4) counting of immunostained cells or measurement of positively stained area; 5) measurement of the positively stained areas, by assessment of both the widening and the strength of the staining. Six to eight high-power fields were analyzed for each section and three sections per sample were evaluated. The histopathological evaluation was performed excluding necrotic areas. Results were expressed as mean ± SD of positive cells per field (F4/80, Ly-6G, CD11b/Gr-1, CD3, CD8, CD4, Foxp3, NKp46/RORγt), or mean percentage ± SD of positive cells/number of total cells per field (Ki67 or PCNA, and TUNEL positive cells, for proliferation and apoptotic index, respectively) or as a mean percentage ± SD of positively stained areas/total area of the examined field at ×400 (85,431.59 μm^2) (expression of IL30, immunoregulatory and prostate cancer driver genes). TUNEL assay was performed with Click-iT™ TUNEL Colorimetric IHC Detection Kit (Thermo Fisher Scientific) according to the manufacturer's instructions. Microvessels were identified as small tubes or circles marked by CD31 Abs and results were expressed as mean ± SD of positive vessels/field. Immunostained sections were examined by two pathologists in a blind fashion, with very good agreement (κ value = 0.89, 0.80 and 0.85, for the evaluation of proliferation index,

apoptotic index, and microvessel density, respectively). The human prostate tissue samples, for immunostaining with anti-PSCA Abs, were obtained from the institutional Biobank of the Local Health Authority n. 2 Lanciano - Vasto - Chieti (Italy) and the personal data processing complies with Data Protection Laws.

REFERENCES

1. Marty, R., N'soukpoé-Kossi, C.N., Charbonneau, D., Weinert, C.M., Kreplak, L., and Tajmir-Riahi, H.A. Structural analysis of DNA complexation with cationic lipids. *Nucleic Acids Res.* **37**, 849-857 (2009).
2. Sun, W., Ji, W., Hall, J.M., Hu, Q., Wang, C., Beisel, C.L., and Gu, Z. Self-assembled DNA nanoclews for the efficient delivery of CRISPR-Cas9 for genome editing. *Angew Chem. Int. Ed. Engl.* **54**, 12029-12033 (2015).
3. Kube, S., Hersch, N., Naumovska, E., Gensch, T., Hendriks, J., Franzen, A., Landvogt, L., Siebrasse, J.P., Kubitscheck, U., Hoffmann, B., et al. Fusogenic Liposomes as Nanocarriers for the Delivery of Intracellular Proteins. *Langmuir* **33**, 1051-1059 (2017).
4. Immordino, M.L., Dosio, F., and Cattel, L. Stealth liposomes: review of the basic science, rationale, and clinical applications, existing and potential. *Int. J. Nanomedicine* **1**, 297-315 (2006).
5. Sapra, P., and Allen, T.M. Internalizing antibodies are necessary for improved therapeutic efficacy of antibody-targeted liposomal drugs. *Cancer Res.* **62**, 7190-7194 (2002).
6. Kulkarni, J.A., Witzigmann, D., Leung, J., Tam, Y.Y.C., and Cullis, P.R. On the role of helper lipids in lipid nanoparticle formulations of siRNA. *Nanoscale* **11**, 21733-21739 (2019).
7. Sorrentino, C., Ciummo, S.L., D'Antonio, L., Fieni, C., Lanuti, P., Turdo, A., Todaro, M., and Di Carlo, E. Interleukin-30 feeds breast cancer stem cells via CXCL10 and IL23 autocrine loops and shapes immune contexture and host outcome. *J. Immunother. Cancer* **9**, e002966 (2021).
8. Sorrentino, C., D'Antonio, L., Ciummo, S.L., Fieni, C., Landuzzi, L., Ruzzi, F., Vespa, S., Lanuti, P., Lotti, L.V., Lollini PL., et al. CRISPR/Cas9-mediated deletion of Interleukin-30

suppresses IGF1 and CXCL5 and boosts SOCS3 reducing prostate cancer growth and mortality. *J. Hematol. Oncol.* **15**, 145 (2022).

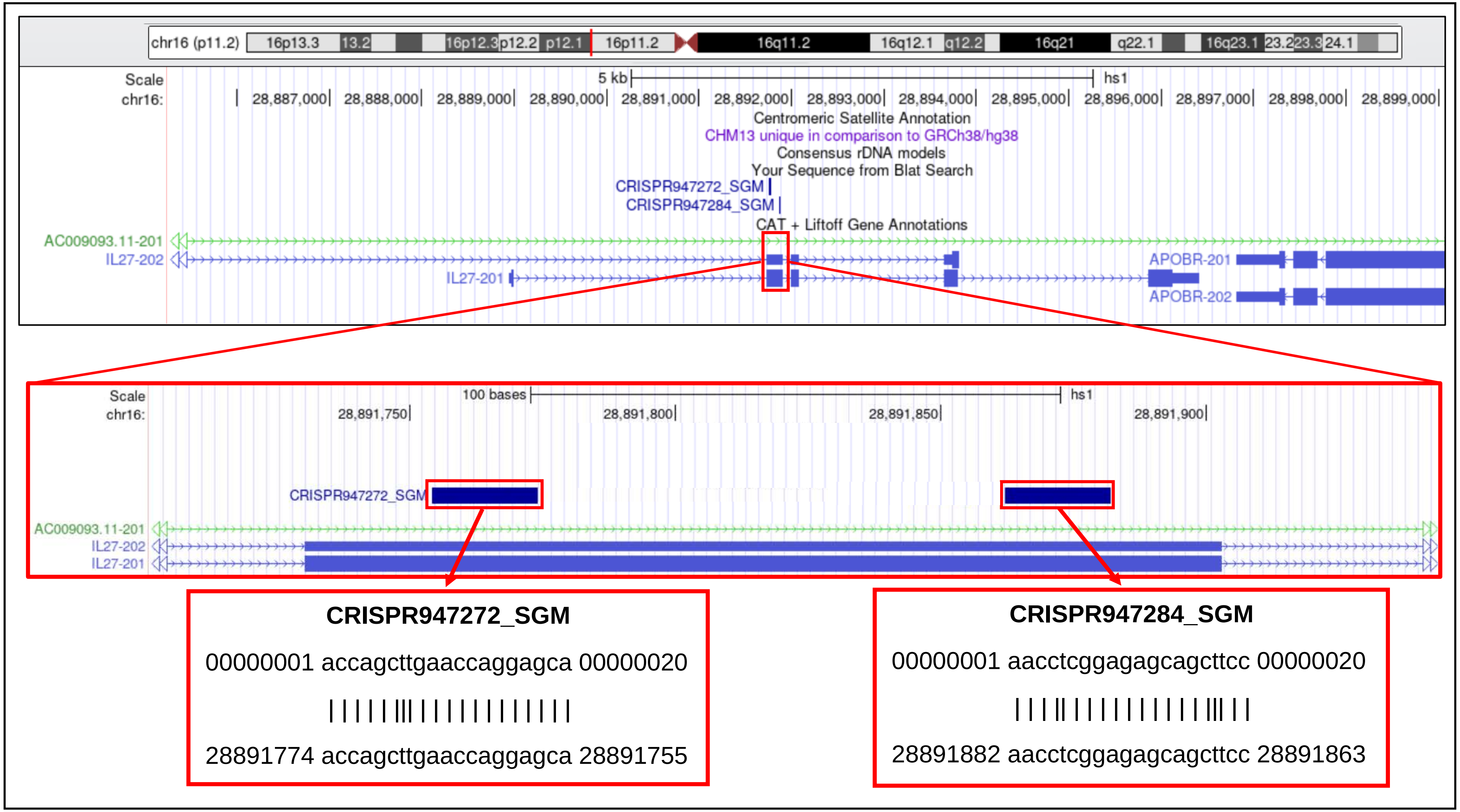

Supplementary Fig. 1. Human sgRNAs used in the study. Graphical representation of human sgRNAs, their target DNA sequences and corresponding alignments (from BLAT Search, *https://genome.ucsc.edu/cgi-bin/hgBlat*).

```
                 1        10        20        30        40        50        60        70        80        90        100       110       120       130
                 |--------+---------+---------+---------+---------+---------+---------+---------+---------+---------+---------+---------+---------|
      mIL30cDNA  ATGGGCCAGGTGACAGGAGACCTTGGCTGGCGGCTCAGCCTGTTGCTGCTACCCTTGCTTCTGGTACAAGCTGGTTCCTGGGGGTTCCCAACAGACCCCCTGAGCCTTCAAGAGCTGCGCAGGGAATTCA
CRISPR283503_SGM
      Consensus  ..................................................................................................................................

                 131     140       150       160       170       180       190       200       210       220       230       240       250       260
                 |--------+---------+---------+---------+---------+---------+---------+---------+---------+---------+---------+---------+---------|
      mIL30cDNA  CAGTCAGCCTGTACCTTGCCAGGAAGCTGCTCTCTGAGGTTCAGGGCTATGTCCACAGCTTTGCTGAATCTCGATTGCCAGGAGTGAACCTGGACCTCCTGCCCCTGGGATACCATCTTCCCAATGTTTC
CRISPR283503_SGM                                                                       TCGATTGCCAGGAGTGAACC
      Consensus  ......................................................................TCGATTGCCAGGAGTGAACC........................................

                 261     270       280       290       300       310       320       330       340       350       360       370       380       390
                 |--------+---------+---------+---------+---------+---------+---------+---------+---------+---------+---------+---------+---------|
      mIL30cDNA  CCTGACTTTCCAGGCATGGCATCACCTCTCTGACTCTGAGAGACTCTGCTTCCTCGCTACCACACTTCGGCCCTTCCCTGCCATGCTGGGAGGGCTGGGGACCCAGGGGACCTGGACCAGCTCAGAGAGG
CRISPR283503_SGM
      Consensus  ..................................................................................................................................

                 391     400       410       420       430       440       450       460       470       480       490       500       510       520
                 |--------+---------+---------+---------+---------+---------+---------+---------+---------+---------+---------+---------+---------|
      mIL30cDNA  GAGCAGCTGTGGGCCATGAGGCTGGATCTCCGGGACCTGCACAGGCACCTCCGCTTTCAGGTGCTGGCTGCAGGATTCAAATGTTCAAAGGAGGAGGAGGACAAGGAGGAAGAGGAAGAGGAGGAAGAAG
CRISPR283503_SGM
      Consensus  ..................................................................................................................................

                 521     530       540       550       560       570       580       590       600       610       620       630       640       650
                 |--------+---------+---------+---------+---------+---------+---------+---------+---------+---------+---------+---------+---------|
      mIL30cDNA  AAGAAAAGAAGCTGCCCCTAGGGGCTCTGGGTGGCCCCAATCAGGTGTCATCCCAAGTGTCCTGGCCCCAGCTGCTCTATACCTACCAGCTCCTTCACTCCCTGGAGCTTGTCCTGTCTCGGGCTGTTCG
CRISPR283503_SGM
      Consensus  ..................................................................................................................................

                 651     660       670       680       690       700  705
                 |--------+---------+---------+---------+---------+----|
      mIL30cDNA  GGACCTGCTGCTGCTGTCCCTGCCCAGGCGCCCAGGCTCAGCCTGGGATTCCTAA
CRISPR283503_SGM
      Consensus  .......................................................
```

Supplementary Fig. 2. Lentiviral vector used for used for TRAMP-C1 cell transfection. Graphical representation of the mouse *Il30* cDNA, cloned into the XbaI-XhoI site of the Tween lentiviral vector used for TRAMP-C1 cell transfection, the specific sgRNA sequence (CRISPR283503_SGM) and their alignment (in red coloured text) (from *Multiple sequence alignment, http://multalin.toulouse.inra.fr/multalin*).

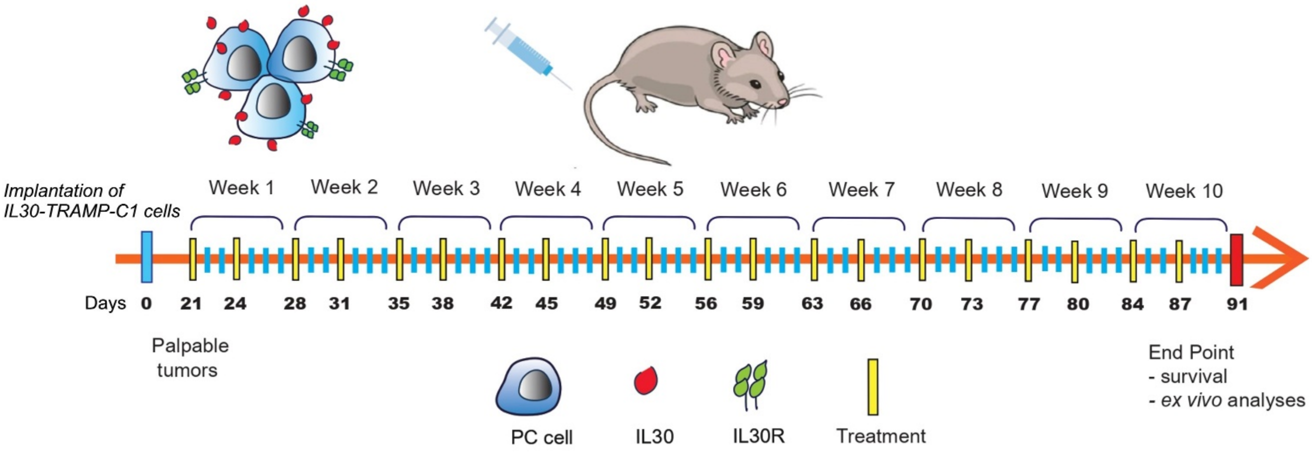

Supplementary Fig. 3. Treatment schedule (orange arrow) applied to C57BL/6J mice, bearing murine-derived subcutaneous IL30-TRAMP-C1 tumors, which overexpress and release soluble IL30. The treatment of two weekly dose of NxPs (10 mg/ml), started when the tumors were palpable (Ø 2mm) and stopped when mice were sacrificed, i.e. when evidence of suffering was observed.

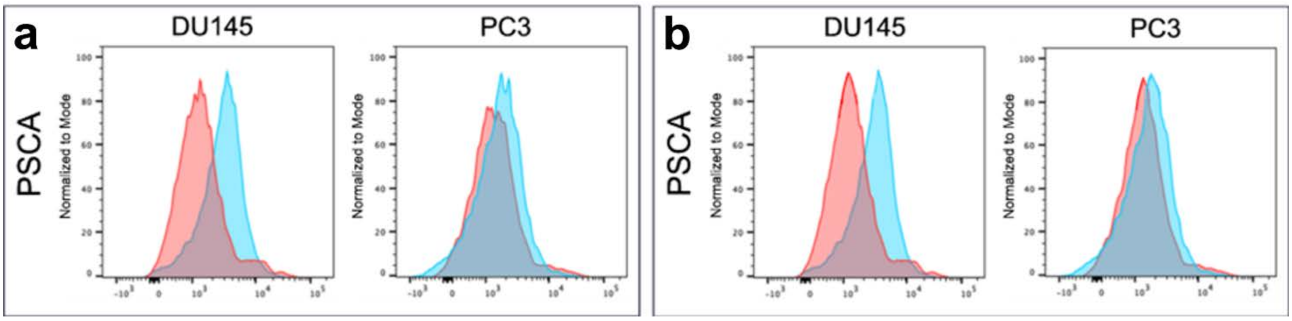

Supplementary Fig. 4. Cytofluorimetric analyses of PSCA expression on the surface of DU145 (picture on the left) and PC3 (picture on the right) cells. Blue areas: specific Abs. Red areas: isotype controls. Panels **a** and **b** represent two out of three biological replicates.

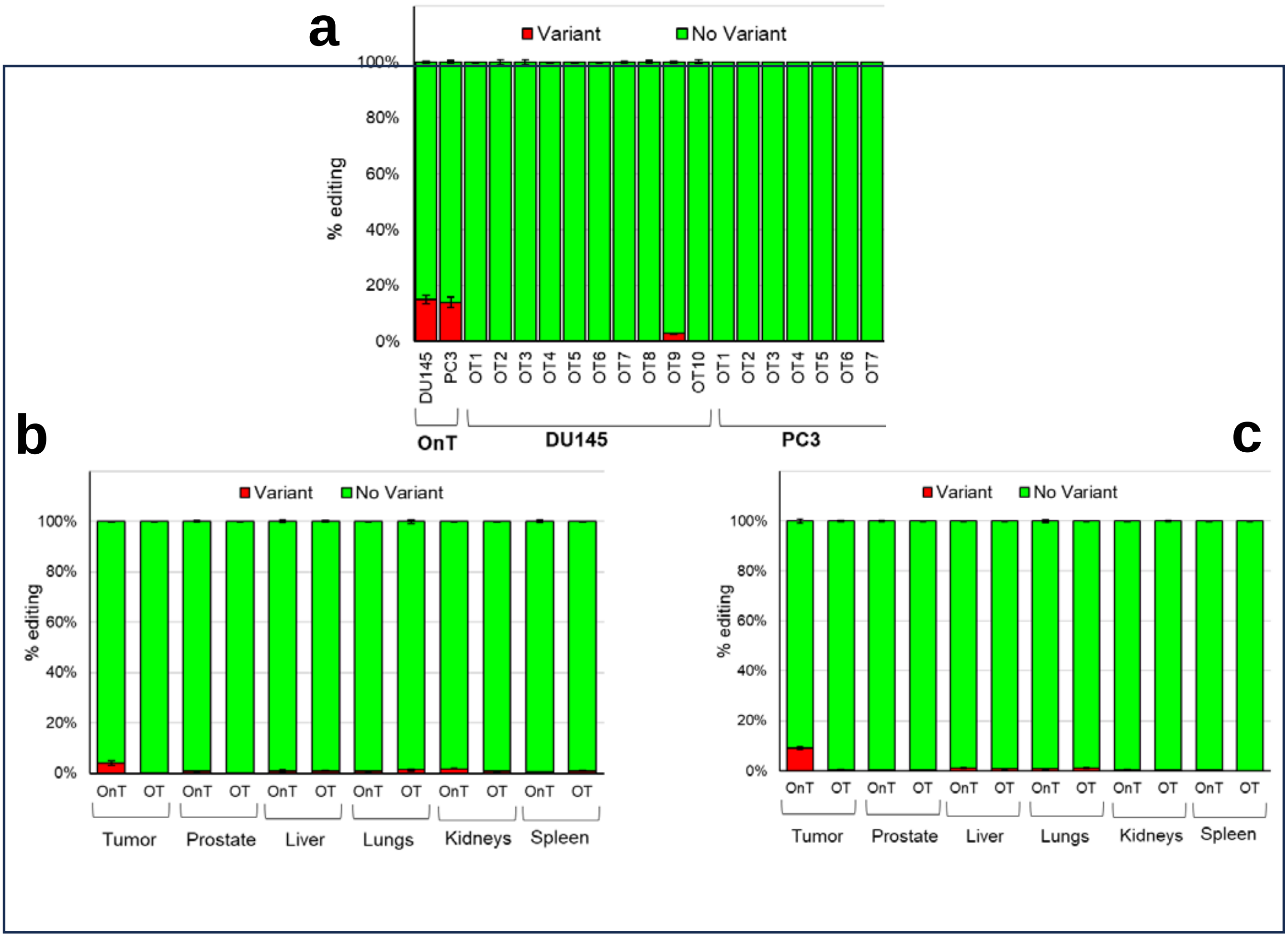

Supplementary Fig. 5.

a. On-target and off-target characterization of Cas9gRNA-mediated *hIL30* editing delivered by unconjugated nanoliposomes, *in vitro*. Average frequency of CRISPR/Cas9-induced variants in the *hIL30* gene (editing efficiency or On-Target effects, OnT) and in off-target sites corresponding to recognized genetic loci (Off-Target effects, OTs), in DU145 and PC3 cell cultures, treated with Cas9hIL30 NxPs.

b. c. On-target and off-target characterization of Cas9gRNA-mediated *hIL30* editing delivered by unconjugated nanoliposomes, *in vivo*. Average frequency of CRISPR/Cas9-induced variants in the *hIL30* gene (editing efficiency or On-Target effects, OnT) and in off-target sites (OTs), in the indicated organs of DU145 **(b)** and PC3 **(c)** tumor-bearing NSG mice, treated with Cas9hIL30 NxPs. Experiments were performed in triplicate.

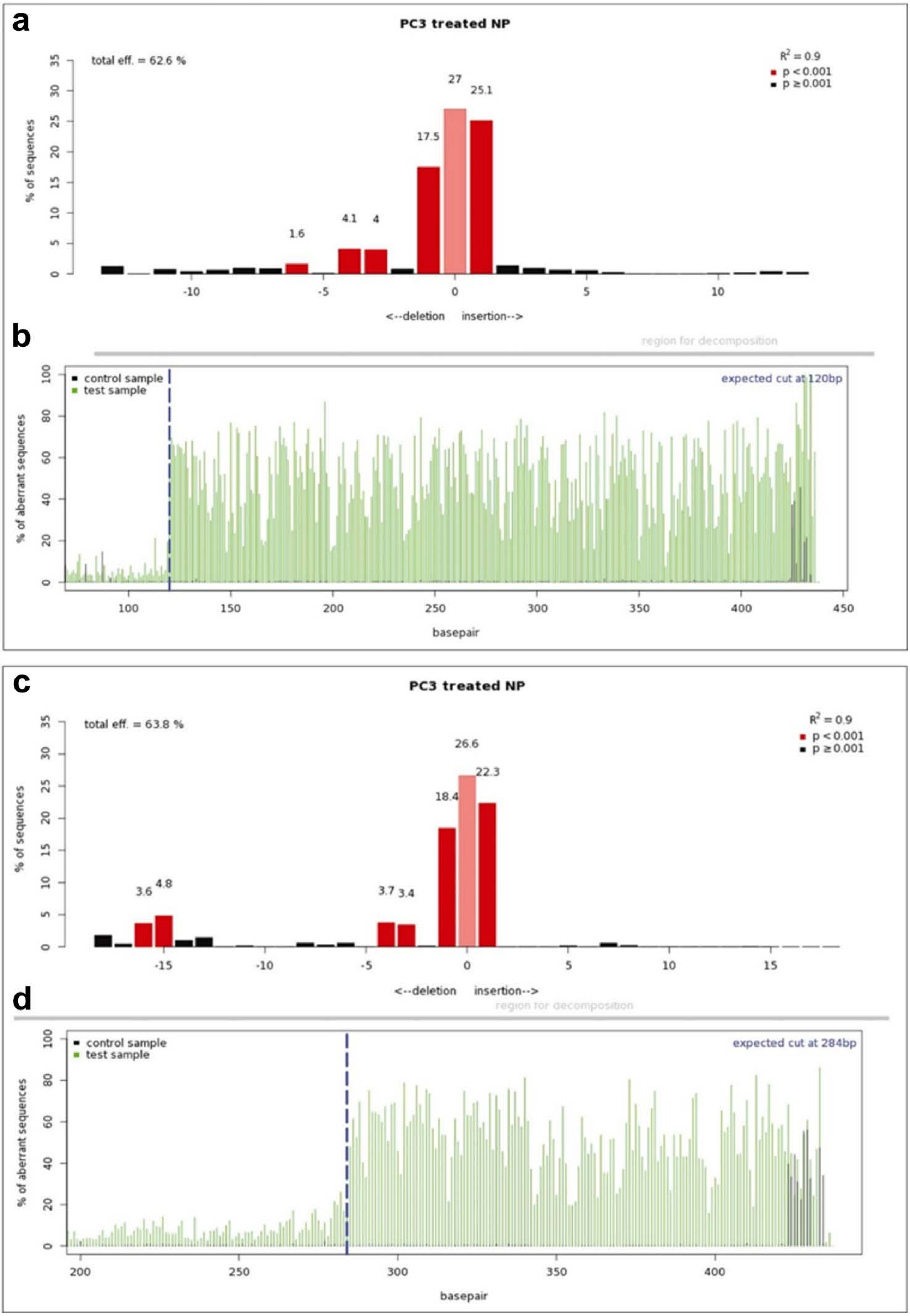
a
PC3 treated NP
total eff. = 62.6 %
R² = 0.9
p < 0.001
p ≥ 0.001
% of sequences
27
25.1
17.5
1.6
4.1
4
<--deletion insertion-->
b
region for decomposition
control sample
test sample
expected cut at 120bp
% of aberrant sequences
basepair
c
PC3 treated NP
total eff. = 63.8 %
R² = 0.9
p < 0.001
p ≥ 0.001
% of sequences
26.6
22.3
18.4
3.6
4.8
3.7
3.4
<--deletion insertion-->
d
region for decomposition
control sample
test sample
expected cut at 284bp
% of aberrant sequences
basepair

Supplementary Fig. 6. TIDE analysis showing the indel spectra and aberrant sequences, within the targeted *hIL30* gene *locus*, in PC3 cells treated with Cas9gRNAhIL30 NxPs *versus* PC3 cells treated with Empty nanoliposomes (controls). The histograms on the top (**a**, **c**) show indel frequencies within ± 20 bp from theoretical gRNA breakpoints. The histograms on the bottom (**b, d**) depict gDNA sequence aberrations in control (black columns) and treated sample (green columns). Theoretical gRNA cuts were indicated by blue lines. Fig. **a** and **b** show the total editing efficiency on the reverse strand of the targeted *hIL30* gene *locus*: 62.6%. Fig. **c** and **d** show the total editing efficiency on the forward strand of the targeted *hIL30* gene *locus*: 63.8%.

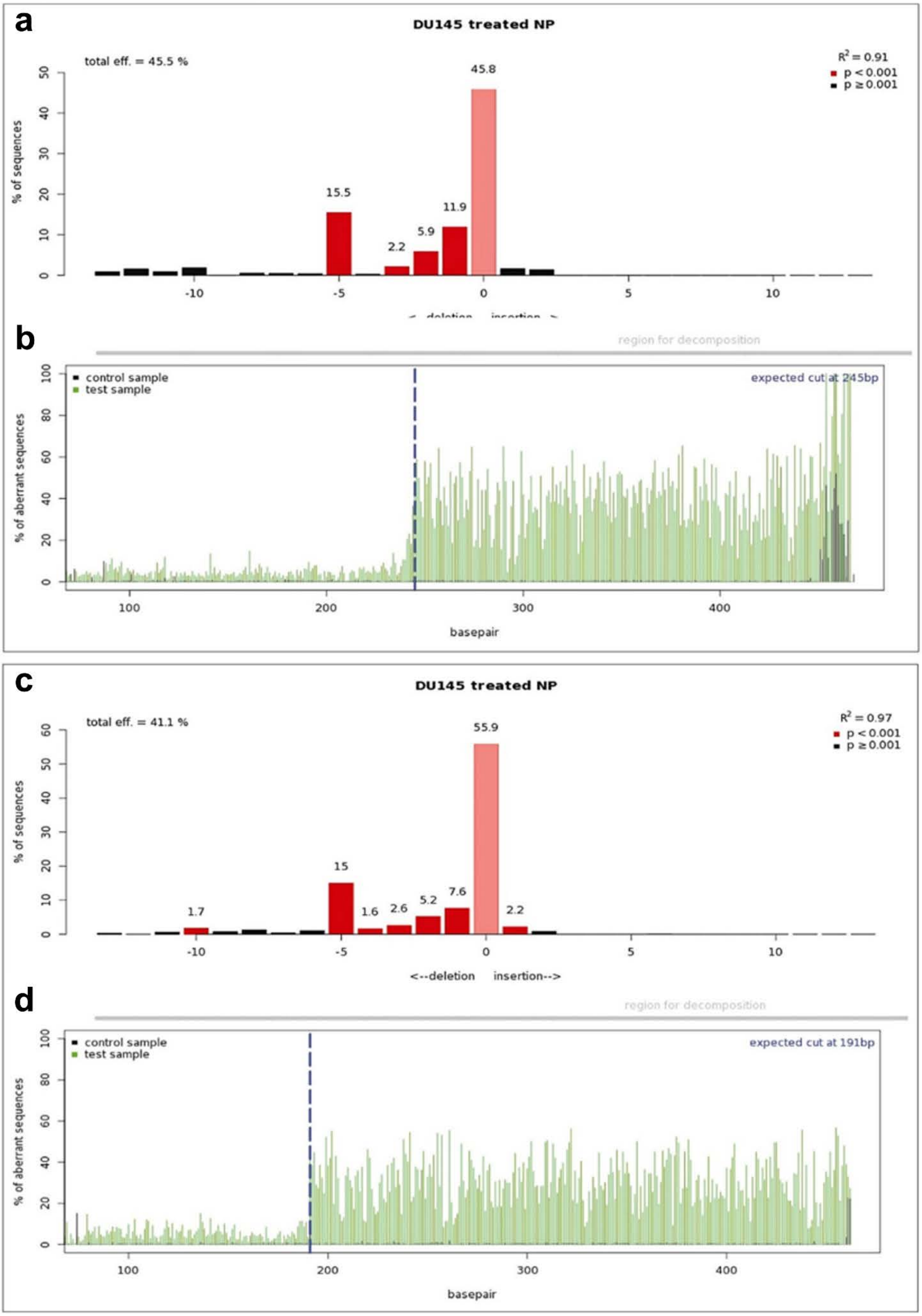
a
DU145 treated NP
total eff. = 45.5 %
$R^2 = 0.91$
p < 0.001
p ≥ 0.001
% of sequences
45.8
15.5
11.9
5.9
2.2
-10
-5
0
5
10
b
region for decomposition
control sample
test sample
expected cut at 245bp
% of aberrant sequences
100
200
300
400
basepair
c
DU145 treated NP
total eff. = 41.1 %
$R^2 = 0.97$
p < 0.001
p ≥ 0.001
% of sequences
55.9
15
7.6
5.2
2.6
1.6
1.7
2.2
-10
-5
0
5
10
<--deletion insertion-->
d
region for decomposition
control sample
test sample
expected cut at 191bp
% of aberrant sequences
100
200
300
400
basepair

Supplementary Fig. 7. TIDE analysis showing the indel spectra and aberrant sequences, within the targeted *hIL30* gene *locus*, in DU145 cells treated with Cas9gRNAhIL30 NxPs *versus* DU145 cells treated with Empty nanoliposomes (controls).

The histograms on the top (**a**, **c**) show indel frequencies within ± 20 bp from theoretical gRNA breakpoints. The histograms on the bottom (**b**, **d**) depict gDNA sequence aberrations in control (black columns) and treated sample (green columns). Theoretical gRNA cuts were indicated by blue lines. Fig. **a** and **b** show the total editing efficiency on the reverse strand of the targeted *hIL30* gene *locus*: 45.5%. Fig. **c** and **d** show the total editing efficiency on the forward strand of the targeted *hIL30* gene *locus*: 41.1%.

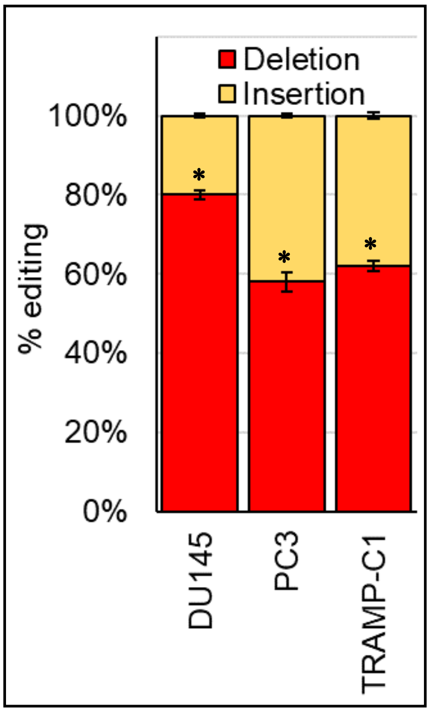

Supplementary Fig. 8. Mutation pattern introduced by Cas9gRNA-mediated editing in *IL30* gene in PC cells. The histogram represents the mutation pattern introduced in human (DU145 and PC3) and murine (TRAMP-C1) PC cells. **p*<0.001, Student's *t*-test *versus* insertion.

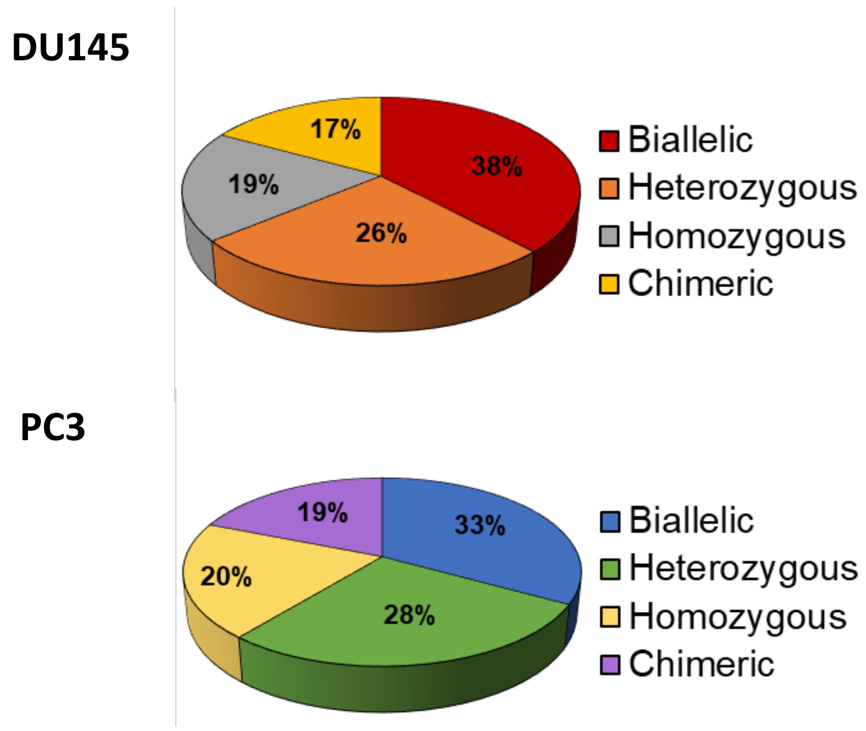

Supplementary Fig. 9. Graphical representation of Cas9gRNA-mediated mutations in PC cells. Allelic distribution of Cas9gRNA-mediated mutations in DU145 and PC3 cell lines, and their frequency.

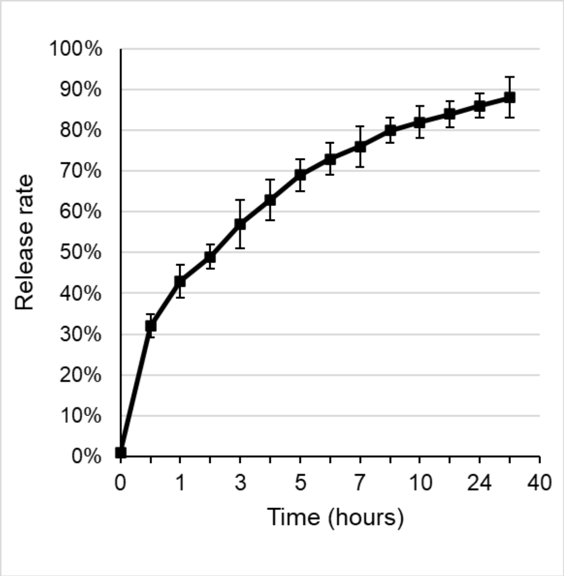

Supplementary Fig. 10. Cas9 release profile of the Cas9hIL30-hPSCA NxPs.

Experiments were performed in triplicate.

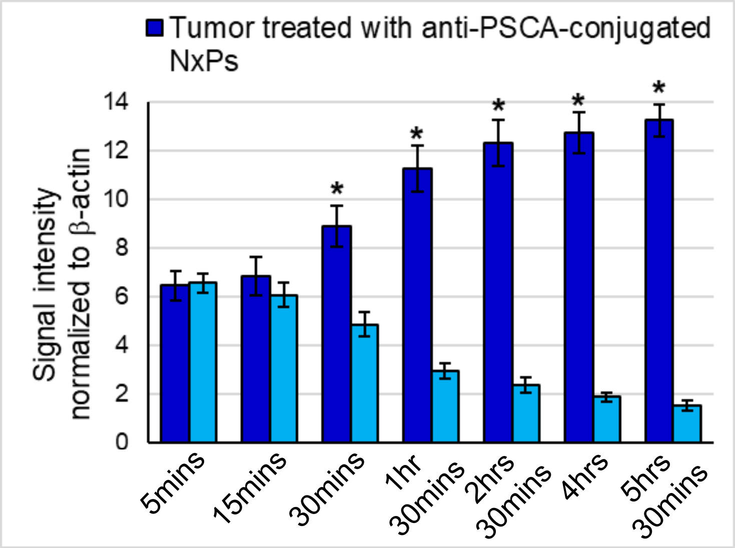

Supplementary Fig. 11. Histogram representing western blotting quantification of Cas9 protein in tumors from mice treated with Cas9hIL30-hPSCA NxPs *versus* tumors from mice treated with unconjugated NxPs, at different time points after nanoparticle injection. Signal intensities were normalized to β-actin expression. ANOVA: $p<0.001$. *$p<0.01$, Tukey HSD test versus tumors of mice injected with unconjugated NxPs, at the same time point.

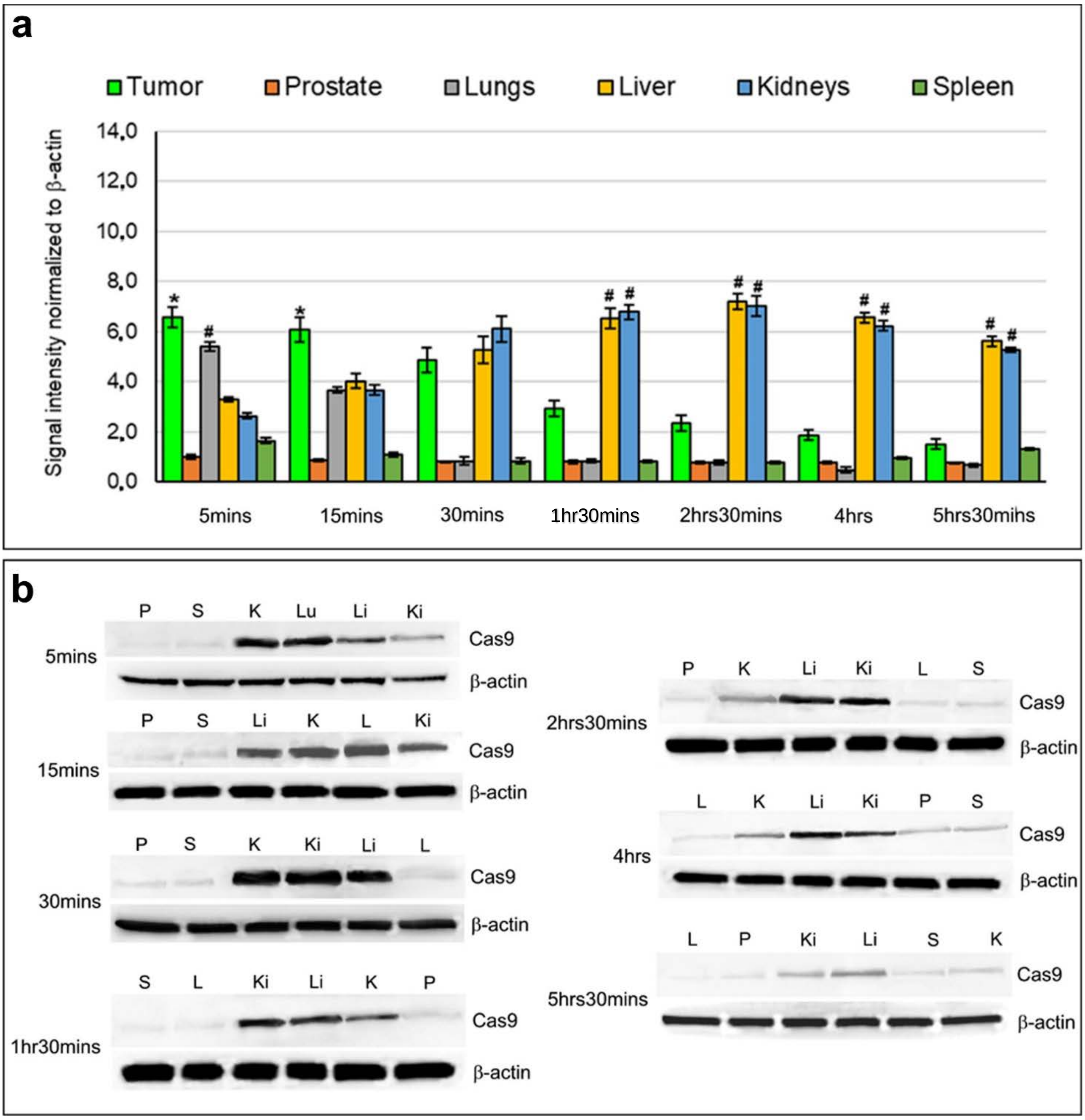

Supplementary Fig. 12. Histogram (a) and images (b) representing western blotting quantification of Cas9 protein, in tumors *versus* organs, in mice treated with unconjugated Cas9hIL30 NxPs, at different time points, after nanoparticle injection. **(a)** Signal intensities normalized to β-actin expression. ANOVA: $p<0.001$. *$p<0.01$, Tukey HSD test *versus* organs at the same time point. $^{\#}p<0.01$, Tukey HSD test *versus* tumor and other organs at the same time point. **(b)** Representative WB images for each time point. K,

tumor; Ki, kidney; Li, liver; Lu, lungs; P, prostate; S, spleen. Molecular weights: β-actin, 42 kDa; Cas9, 160 kDa.

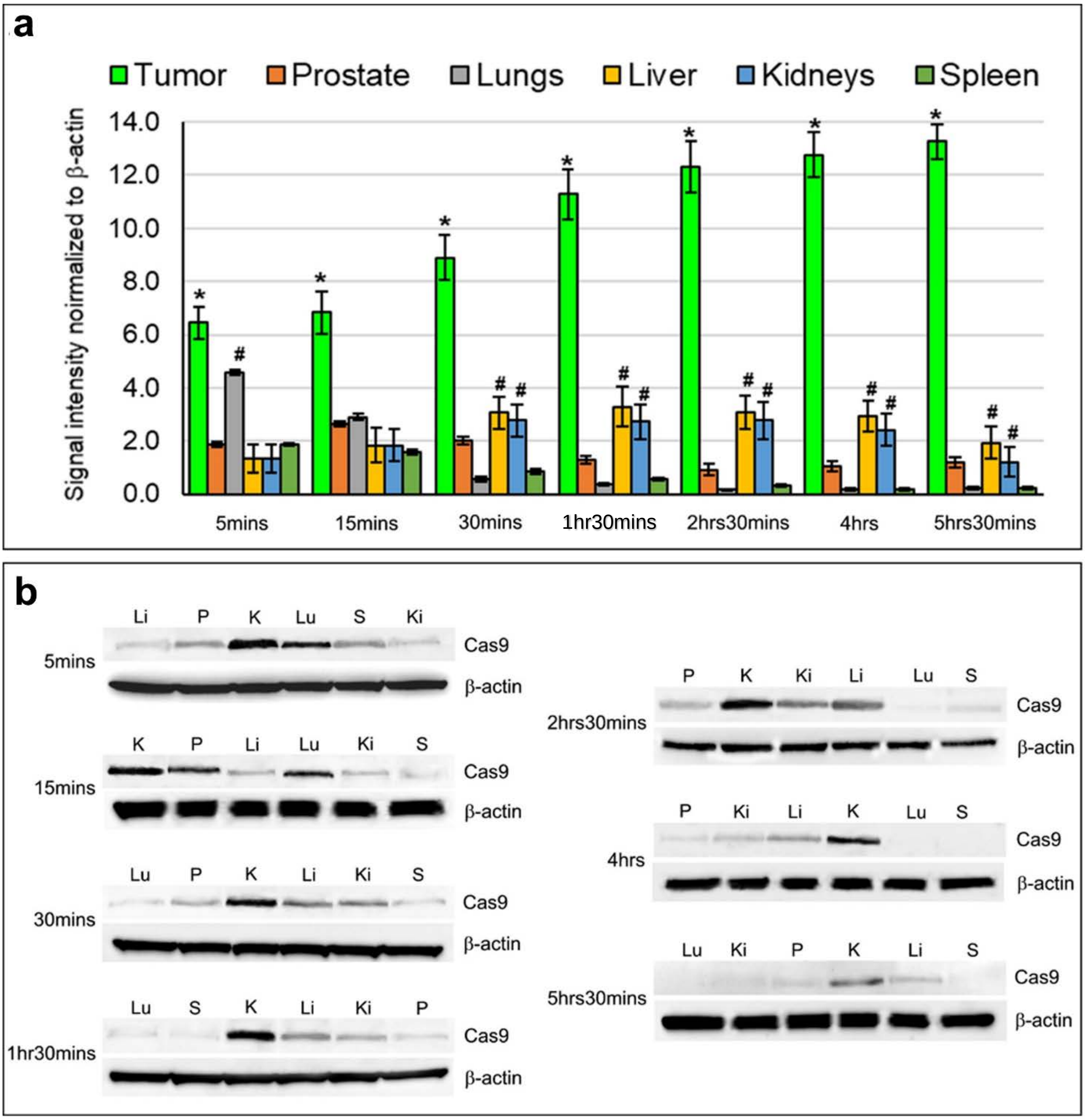

Supplementary Fig. 13. Histogram (a) and images (b) representing western blotting of Cas9 protein, in tumors *versus* organs, in mice treated with Cas9hIL30-hPSCA NxPs, at different time points after nanoparticle injection. (a) Signal intensities normalized to β-actin expression. ANOVA: p<0.001. *p<0.01, Tukey HSD test *versus* organs at the same time point. #p<0.01, Tukey HSD test *versus* other organs at the same time point. **(b)**

Representative WB images for each time point. K, tumor; Ki, kidney; Li, liver; Lu, lungs; P, prostate; S, spleen. Molecular weights: β-actin, 42 kDa; Cas9, 160 kDa.

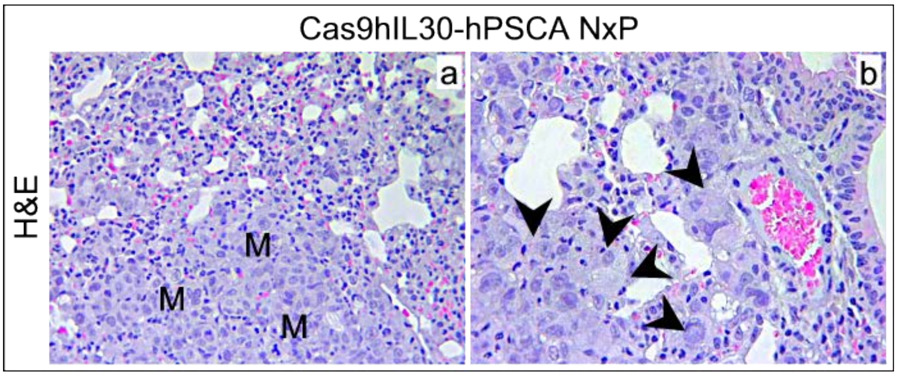

Supplementary Fig. 14. Lung metastasis developed in Cas9hIL30-hPSCA NxP treated NSG mice. Histopathological features of lung metastasis (magnification: **a,** X200; **b**, X400) developed in Cas9hIL30-hPSCA NxP treated mice bearing PC3 tumors are comparable to those observed much earlier (at the time of sacrifice, 55 days *versus* 63 days after s.c tumor cell implantation) in Empty-hPSCA NxP treated mice bearing PC3 tumors (Fig. 4g, a, in the main text). **M,** metastasis. Arrowheads indicate metastatic cancer cells accumulated in the pulmonary interstitium, around the vessels and in the peribronchial area. Magnification: **a**, X200; **b**, X400.

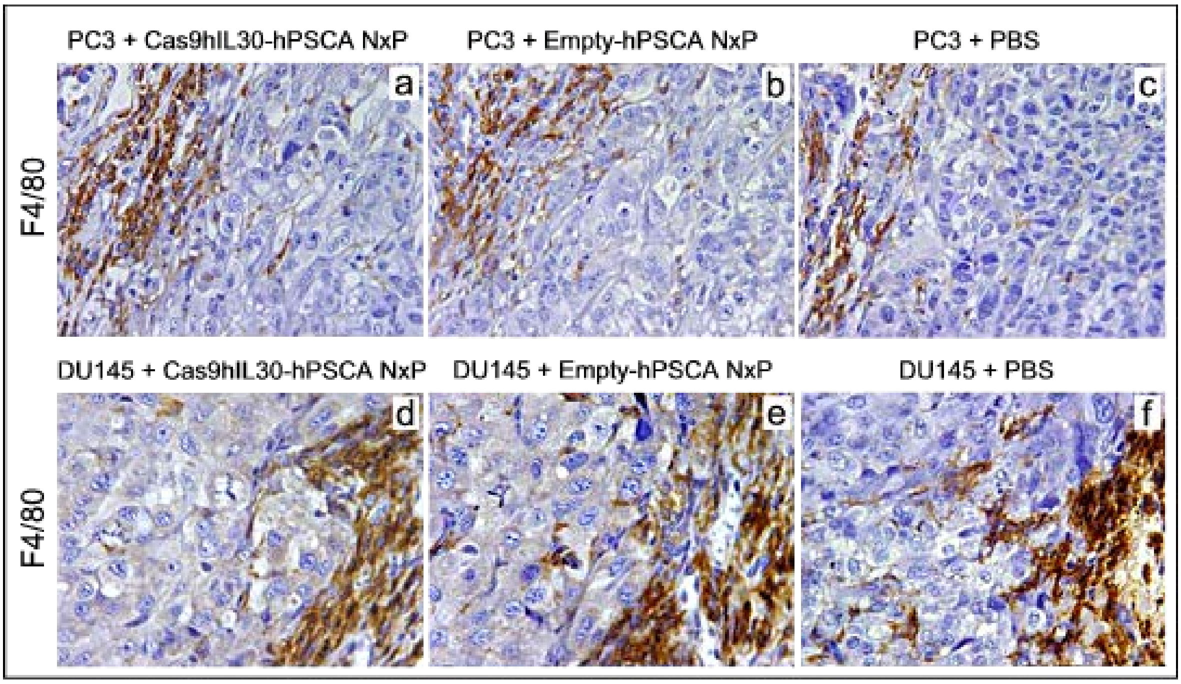

Supplementary Fig. 15. Immunohistochemical detection of macrophages in PC xenografts of mice treated with Empty-hPSCA and Cas9hIL30-hPSCA NxPs.

The macrophage cell content, visualized by anti-F4/80 immunostaining, was primarily detected at the edge of PC3 (**a**, **b**, **c**) and DU145 (**d**, **e**, **f**) tumors and was similar after the treatment with Cas9hIL30-hPSCA (**a**, **d**) or Empty-hPSCA (**b**, **e**), or PBS (**c**, **f**). Magnification: X400.

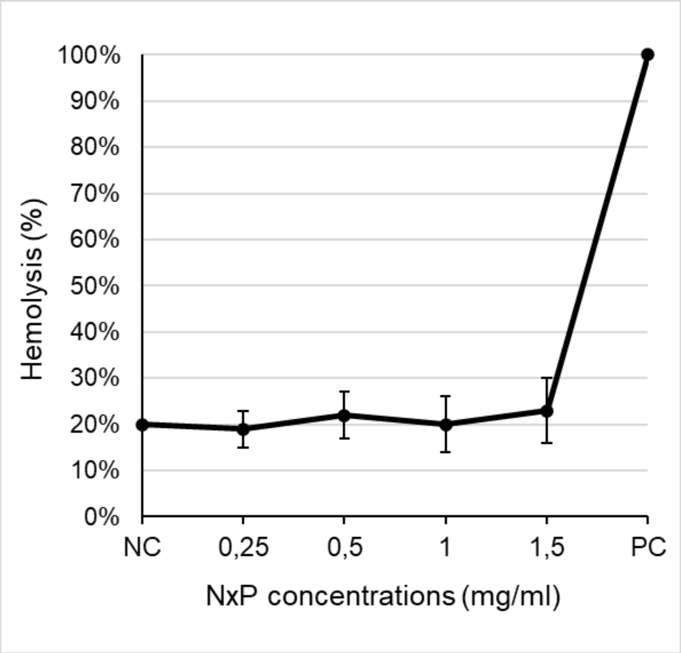

Supplementary Fig. 16. *In vitro* hemolysis induced by different concentrations of Cas9hIL30-hPSCA NxPs. Untreated blood sample was used as negative control (NC) and Triton X-10% treated blood was used as positive control (PC). Data are presented as mean ± SD. Experiments were performed in triplicate.

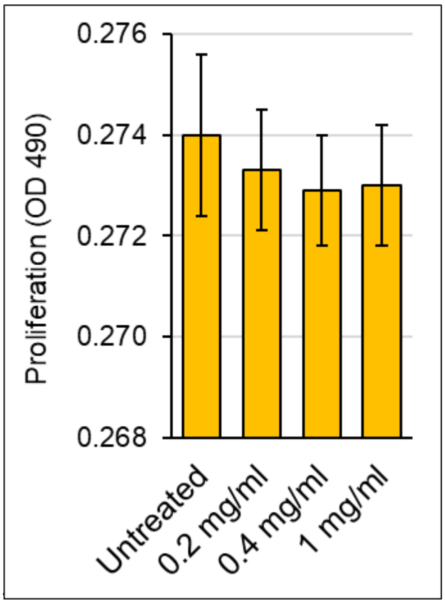

Supplementary Fig. 17. Viability of IL30-TRAMP-C1 cells after 48h incubation with different concentrations (0.2, 0.4, 1.0 mg/ml) of Empty-mPSCA NxPs *versus* PBS treated cells. ANOVA, $p>0.05$. Results obtained from untreated cells were comparable to those from PBS treated cells. Experiments were performed in triplicate.

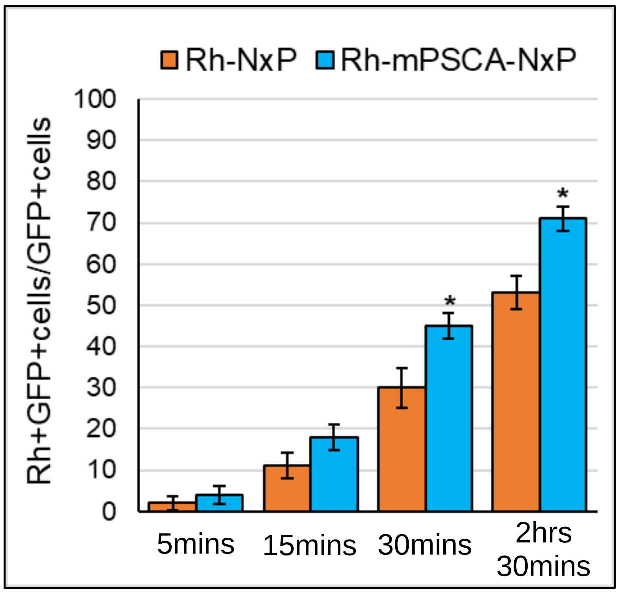

Supplementary Fig. 18. Quantification, by LSC, of the NxP uptake in TRAMP-C1 tumors developed in C57BL/6J mice. The tumor uptake of the Rh-NxPs (orange) and Rh-mPSCA-NxPs (light blue) was expressed as the mean percentage ± SD of $Rh^{+}GFP^{+}$cells/total number of GFP^{+}cells. *p<0.01, Student's *t*-test *versus* Rh-NxPs at the same time point.

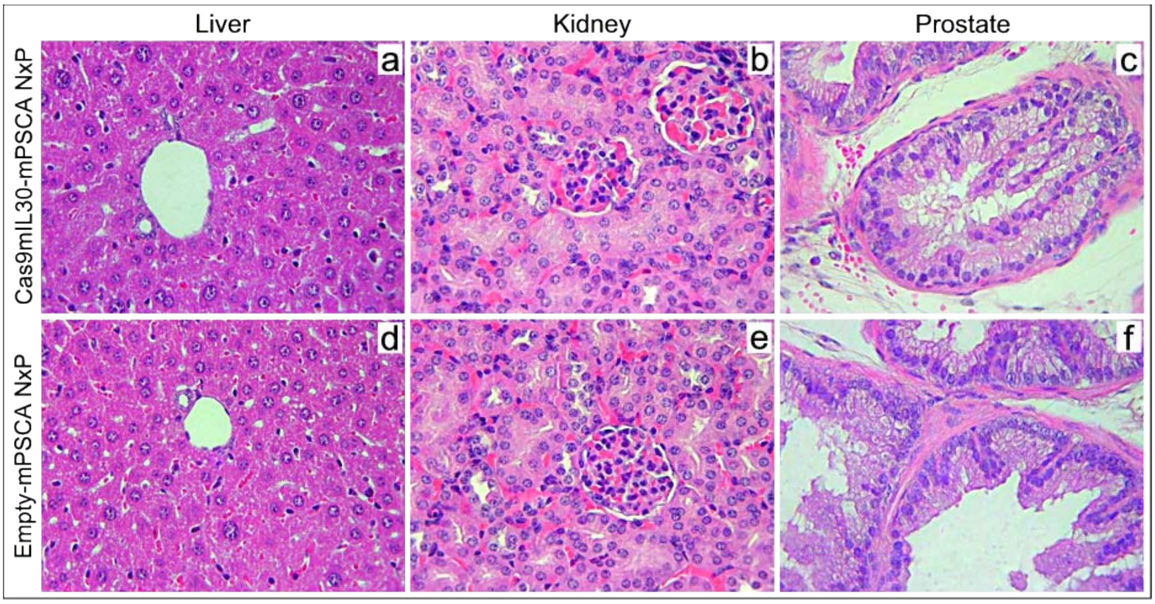

Supplementary Fig. 19. Histological aspects of the organs of IL30-TRAMP-C1 tumor-bearing mice treated with Empty-mPSCA NxPs or Cas9mIL30-mPSCA NxPs. H&E staining shows that liver (**a**), kidneys (**b**) and prostate (**c**) of tumor-bearing C57BL/6J mice, treated with Cas9mIL30-mPSCA NxPs, are histologically normal, with no signs of cell damage, and fully comparable to those of Empty-mPSCA NxP treated mice (**d, e, f**). Similar results were obtained from histopathological analysis of the organs of PBS treated mice. Magnification: X400.

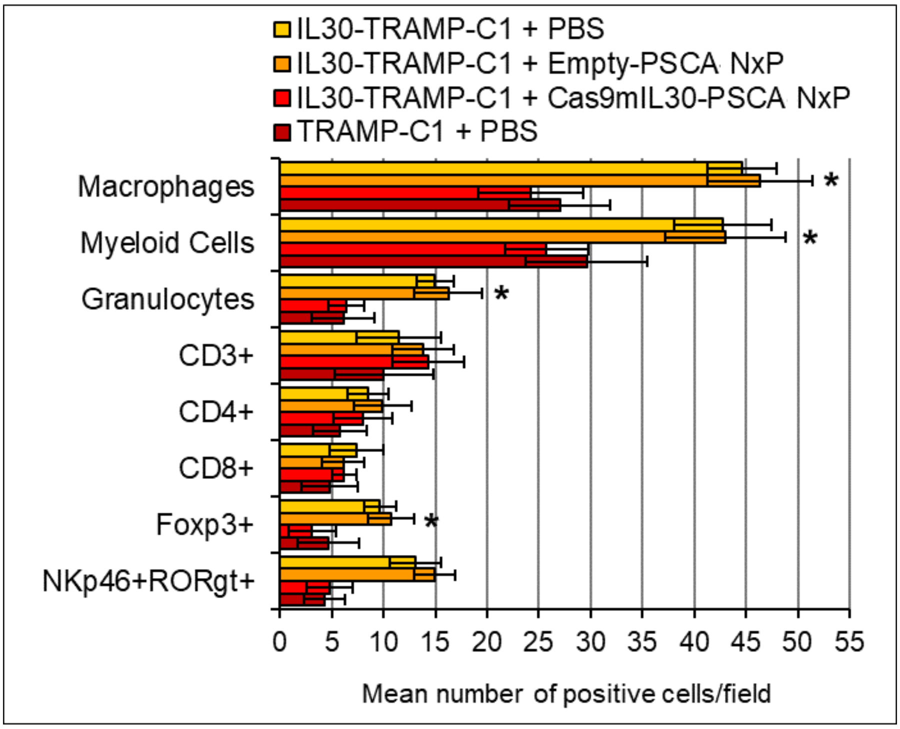

Supplementary Fig. 20. Automated immune cell count in IL30-TRAMP-C1 and wild type TRAMP-C1 tumors from C57BL/6J mice treated with Cas9mIL30-mPSCA NxPs, or Empty-mPSCA NxPs, or PBS. Immune cell populations were assessed by immunohistochemistry, as described in the Supplementary Methods. *ANOVA, $p<0.001$; $p<0.01$, Tukey HSD test compared with IL30-TRAMP-C1 + Cas9mIL30-mPSCA NxPs, IL30-TRAMP-C1 + PBS and TRAMP-C1 + PBS.

Supplementary Table 1. Information about the TrueGuide sgRNAs used for *IL30* gene editing and the corresponding primers for sequencing.

Product code[a]	Target DNA Sequence	PAM Sequence	Target locus	Strand	Primers
**CRISPR947272_SGM[b]**	ACCAGCTTGAAC CAGGAGCA	AGG	Chr.16: 28504007 - 28504029 on GRCh38	For	*For:*CTCACTCACAGAGAGG CGGC<br>*Rev:*TTTTGAGGAGGCAGAG GAATGATG
**CRISPR947284_SGM[c]**	AACCTCGGAGAG CAGCTTCC	TGG	Chr.16: 28503899 - 28503921 on GRCh38	For	*For:*CTCCAGCTCAATCTCCA GCCTCATC<br>*Rev:*TGTTCCCTTCCTTCTCA AGCTCTCT
**CRISPR283503_SGM[d]**	TCGATTGCCAGG AGTGAACC	TGG	Chr.7: 126592656 - 126592678 on GRCm38	For	*For:*TGTCCACAGCTTTGCTG AAT<br>*Rev:*GGGATGACACCTGATT GGGG

[a.] All sgRNAs and primers are designed and synthetized by Thermo Fisher Scientific (Waltham, MA, USA).
[b.] Used in human DU145 cells.
[c.] Used in human PC3 cells.
[d.] Used in murine TRAMP-C1 cells.
For, forward. Rev, reverse.

Supplementary Table 2a. Off-target sites associated with the TrueGuide sgRNA CRISPR947272_SGM, used for *IL30* gene editing in DU145 cells.

Gene	Target DNA Sequence	Score	Mismatch	Position	Strand	Editing (%)
AKAP4	ACCATCTGGAACCAA AAGCA	24	4	chrX:50193177	Rev	0.000009
SLC6A6	ACCAGCA-GCACCAGGAGCA	33	3	chr3:14466549	Rev	0.003935
SEC24B	ACCAGC---AACCAGGAGCA	48	3	chr4:109463089	For	0.000555
SGMS2	AAAAGCTTCAAACAG GAGCA	49	4	chr4:107913159	Rev	0.436129
ABCB8	ACCAGCTGGC-CCAGGAGCA	49	3	chr7:151034312	Rev	0.203314
NONO	ACCAGCTGGCAC-AGGAGCA	56	3	chrX:71298743	Rev	0.042114
WDR45B	GCCGGCTTCAACCAG GACCA	58	4	chr17:82648275	Rev	0.013314
LINC00479	GCCAGCTGACACCAG GAGCA	60	4	chr21:41711871	Rev	0.000314
LINC00479	GCCAGCTGACACCAG GAGCA	60	4	chr21:41711826	Rev	**2.854555**[a]
NPS	ACCAGATTGGA-CAGGAGCA	61	3	chr10:127552507	For	0.433269
Non coding	AACACCATGTACCAG GAGCA	15	4	chr8:34578630	For	0.002144
"	GACAGCTGGAACCAG AAGCA	16	4	chr17:72774187	For	0.001472
"	TCCTGC-TGAACCAGGAGCA	18	3	chr17:36706558	For	0.001424
"	TCCATCTTG-ACCAGGAGCA	22	3	chr5:14472510	For	0.000442
"	ATCAGCTGGAACCAG GAACA	23	3	chr6:42083630	For	0.001366
"	AACCGCTTGAACCCA GGAGCA	26	3	chr11:13060160	For	0.002093
"	GACAGCTTGAA-CAGGAGCA	26	3	chr10:58895592	For	0.002165
"	AATAGCTTGAACCCA GGAGCA	29	3	chr19:54678580	For	0.000883
"	AATAGCTTGAACCCA GGAGCA	29	3	chr9:84347086	For	0.002203
"	ACCACCACGAATCAG GAGCA	29	4	chr2:231427361	Rev	0.000128
"	GTCAGCCTGAATCAG GAGCA	32	4	chr3:137921740	For	0.000343
"	AACTGCTTGAACCCA GGAGCA	33	3	chr16:4567783	Rev	0.000343
"	ACCCACTAGAACCAG AAGCA	34	4	chr11:120840397	Rev	0.000783

“	ACCTGATGGGACCAG GAGCA	35	4	chr6:105503028	Rev	0.001814
“	AATAGCTTGAACCCA GGAGCA	36	3	chr4:138979078	Rev	0.000142
“	AACTGCTTGAACCAG GAGCT	36	3	chr13:40936857	For	0.000280
“	CCAAGCTAGAGCCAG GAGCA	36	4	chrX:111263448	For	0.001166
“	ACCAGCTGGTACAAA GAGCA	36	4	chr11:87596706	For	0.001037
“	GCCAGCGGGTACCAG GAGCA	36	4	chr5:11181102	For	0.000479
“	ACCACCCTGAACCAA AAGCA	36	4	chr11:73279419	Rev	0.001017
“	ACCATCTTG-ACCAGAAGCA	38	3	chr2:190178288	For	0.000941
“	TCCAGCCTGAACAAA GAGCA	38	4	chr13:96897488	For	0.001272
“	CCCAGCTGCAGCCAG GAGCA	39	4	chr2:100106649	Rev	0.001201
“	GCCAGCAATAACCAG GAGCA	39	4	chr7:81209033	For	0.000977
“	GCCGGCTTTAGCCAG GAGCA	40	4	chr5:132668077	Rev	0.001104
“	GCCAGC-TGAACCAGGAACA	41	3	chr3:53222320	For	0.001923
“	GCCAGCTGCCACCAG GAGCA	42	4	chr3:184196930	For	0.001444
“	ATCAGACTTTAACCAG GAGCA	44	3	chr4:31338955	For	0.002269
“	AACAGCTT-AACAAGGAGCA	44	3	chr1:16399771	For	0.000546
“	AACAGCACAAACCAG GAGCA	44	4	chr18:69870664	For	0.000287
“	ACCTG-TTGAATCAGGAGCA	45	3	chr10:78913051	Rev	0.001633
“	TCCACCTGGAAGCAG GAGCA	45	4	chr12:52414929	For	0.000766
“	AACAGATTGAACTAAG AGCA	45	4	chr5:115090075	For	0.000476
“	AATAGCTTCCACCAG GAGCA	45	4	chr8:116828165	For	0.000128
“	TCCAGCTTGAACTGA GAGCA	45	4	chr3:169632096	For	0.000782
“	CACAGCTGGATCCAG GAGCA	46	4	chr3:9009044	Rev	0.002176
“	ACCATCTATGAACCA GGAGTA	47	3	chr3:77696449	Rev	0.000092
“	ACCAG---GAACCAGGAGCA	47	3	chr16:55995281	For	0.0005
“	ACCAGCTGGAATAAA GAGCA	48	4	chr16:24228611	For	0.000601

“	ACAGGCTGGAACCAG GAGAA	48	4	chr5:3962664	For	0.000768
“	ACGATCTGGAACCAG GAGAA	48	4	chr18:62020419	For	0.001784
“	ACAAGCTAGTACCAG GAACA	48	4	chr6:158482446	For	0.000656
“	ATCAGGCTTGAACCA GGAGAA	49	3	chr5:5842928	For	0.001308
“	ACCTGCT-GAAGCAGGAGCA	49	3	chr3:51505610	Rev	0.000785
“	TCCAGCGTG-ACCAGGAGCA	49	3	chr8:143269821	For	0.000829
“	ACCAGCTGGCA-CAGGAGCA	49	3	chr16:69756723	For	0.001543
“	GCCAGCATGAAGCAG GAGCA	49	3	chr22:40721497	Rev	0.001459
“	AAGAGCCTGAACCAG GAGCT	49	4	chr10:27713508	For	0.000091
“	ATCA-CTTGAACCTGGAGCA	50	3	chr20:14608167	For	0.000543
“	ACCTGCTAGAACCAG GAGCT	50	3	chr1:118663538	Rev	0.001168
“	ATCA-CTTGAACCTGGAGCA	50	3	chr22:40217886	For	0.001925
“	ATCA-CTTGAACCCGGAGCA	51	3	chr19:37730314	Rev	0.000024
“	ACCAGCTGGAACCAA AGAGCA	51	3	chr10:93461677	For	0.001402
“	CACAGCTTGAAACAG GAACA	51	4	chr15:72369152	For	0.002265
“	GCCATCTTGAACCAG AAGCT	52	4	chr2:75592683	Rev	0.000425
“	ACTAGCTCTAAACAG GAGCA	53	4	chr17:35665736	For	0.001103
“	ACCAGCAGTAAACAG GAGCA	53	4	chr14:54727874	For	0.001628
“	TCCACCTTAAACCAGA AGCA	54	4	chr20:23916100	For	0.000058
“	AACATCATGAACCAG GAGGA	54	4	chr1:173182307	For	0.000362
“	ACCCACGTGCACCAG GAGCA	54	4	chr2:86055352	For	0.000496
“	ACCAGCTGGGACAGG GAGCA	54	4	chr2:85447046	For	0.001074
“	GCCAGCTTGGACCA-GAGCA	55	3	chr1:225972781	For	0.000603
“	TCCACCTTCACCCAG GAGCA	55	4	chr4:24462921	For	0.001105
“	ACTGGCTTGGACCAG GACCA	57	4	chr20:1632524	Rev	0.000477
“	ACCAGCCT--ACCAGGAGCA	58	3	chr12:70045727	For	0.000000

“	ACCTGCTAGAAACAG AAGCA	58	4	chr4:121653259	For	0.001200
“	ACCACCTGGAACCAA GTGCA	58	4	chr22:46141004	Rev	0.001714
“	ACCACCTGGAACCAA GTGCA	58	4	chr15:20282097	Rev	0.001590
“	ACCAGCAAGCACCAG AAGCA	58	4	chr4:39987587	For	0.001964
“	CCCACCCTGAACCAG GAGAA	58	4	chr19:41135150	Rev	0.002142
“	ACCACCTGGAACCAA GTGCA	58	4	chr16:33572301	For	0.001658
“	ACCAGCTGGAA-- AGGAGCA	59	3	chr6:46028265	Rev	0.000509
“	ACCAGATA- AACCAGGAGCA	59	3	chr4:88444112	Rev	0.000093
“	AACTGCTTGAACCGG GACCA	59	4	chr17:4915784	For	0.002052
“	ACAAGCTTGACCCAG GAACA	60	3	chr10:80697112	Rev	0.001017
“	ACCAAATTGAAACAAG AGCA	60	4	chr8:60809438	For	0.000001
“	ACCACCTTGTACCAG AATCA	60	4	chr6:15759617	For	0.000358
“	ACCAGAGTGGACCAG GAGCA	62	3	chr11:78051348	Rev	0.001021
“	ACCAACTTGTACCAG GAGGA	62	3	chr12:53792472	Rev	0.000020
“	TCCAGGTGGGACCAG GAGCA	62	4	chr11:64137808	For	0.001914
“	GCAAGCCTGAACCAG GAGCC	62	4	chr3:45990269	For	0.000757
“	ATTAGCTTGGAGCAG GAGCA	62	4	chr6:54452253	For	0.001339
“	CCCAGCCTGACCCAG AAGCA	62	4	chrX:119211723	Rev	0.002161
“	ACCAGCTAGAA- CAGGAACA	63	3	chr17:7452536	For	0.001540
“	ACCTGCTTGAAAAAG AAGCA	63	4	chr5:32784780	Rev	0.002067
“	ACCTGCATGCCCCAG GAGCA	63	4	chr18:5994117	Rev	0.001326
“	AACAGCTTCTCCCAG GAGCA	63	4	chr22:20880882	Rev	0.000002
“	CCCAGCATGCCCCAG GAGCA	63	4	chr13:49108453	Rev	0.000725
“	CTCAGCTAGAACCAG GAGCC	63	4	chr5:1049501	Rev	0.001830

[a] Highest off target editing observed.
For, forward.
Rev, reverse.

Supplementary Table 2b. Off-target sites associated with the TrueGuide sgRNA CRISPR947284_SGM, used for *IL30* gene editing in PC3 cells.

Gene	Target DNA Sequence	Score	Mismatch	Position	Strand	Editing (%)
LOC105373212	AGGCTCGAAGAGCGGCTTCC	25	4	chr1:235972047	Rev	0.630341
CLIP3	AACCCCTCAGAGCAGATTCC	38	4	chr19:36015892	Rev	0.393469
LOC100128253	AAACCCGGAGAGAGGCTTCC	41	4	chr12:3306401	For	0.464932
LOC105369520	AGCCTCTGAGAGAAGTTTCC	53	4	chr11:119642509	Rev	0.063768
ROPN1L-AS1	AACTACGGAGAGCTACTTCC	55	4	chr5:10441113	Rev	2.044850
HTT	AGCCT-GGAGAGCAGCTTCT	66	3	chr4:3215118	For	0.003314
DYRK4	AACCTATGACACCAGCTTCC	68	4	chr12:4610239	For	**3.203314**[a]
Non coding	GACCACTCAGAGCAGCTTCC	11	4	chr10:132301396	Rev	0.071200
"	TACCTCTGTAGAGCAGCTTCC	14	3	chrX:108257745	Rev	0.033800
"	AGCCTCTTAGAGCAGCTTCC	20	3	chr2:68063335	For	0.049600
"	ATCCTC-AAGAGCAGCTTCC	21	3	chr6:123638442	For	0.055600
"	CACCCCAGAAAGCAGCTTCC	21	4	chr8:139074941	For	0.018200
"	TGCCTCGGAG-GCAGCTTCC	26	3	chr22:46012541	Rev	0.025200
"	AAGCCCGG-GAGCAGCTTCC	27	3	chr11:2095041	For	0.020200
"	GACCCCAGAGAACAGCTTCC	27	4	chr19:58369547	For	0.059400
"	CATCTCGG-GAGCAGCTTCC	28	3	chr10:132503633	For	0.028400
"	CACCTCAGGAAGCAGCTTCC	28	4	chr1:189583295	For	0.080400
"	TACTTCAGAGAGCAGATTCC	29	4	chr1:237974355	For	0.061600
"	AACC-CAGAGAGCAGCTTCA	30	3	chr4:24145998	For	0.034800
"	CAGCTCTGAAAGCAGCTTCC	30	4	chrX:139093370	Rev	0.068600
"	CACCTCTCAGAGAAGCTTCC	30	4	chr3:83938323	For	0.075800
"	AAGATCTGAGAGCAACTTCC	32	4	chr2:48525311	Rev	0.033600
"	AAACTCAAAGAGCAGATTCC	34	4	chr20:18984624	For	0.012600

“	GACATCAGAGAGAAG CTTCC	34	4	chr6:140438406	For	0.086800
“	AAACGCGGAGAG-AGCTTCC	35	3	chr2:71699954	For	0.000990
“	AACTTCAGAGAGCAA CTTCA	35	4	chr10:89251516	Rev	0.000672
“	AAACTCTGAGA-CAGCTTCC	37	3	chr2:222805739	For	0.000956
“	AACC-CAGAGAGCAGCATCC	38	3	chr16:17481252	Rev	0.000974
“	AGTCTCAGAGAGCAG CATCC	38	4	chr2:87541615	Rev	0.000050
“	AGTCTCAGAGAGCAG CATCC	38	4	chr2:111409177	For	0.000068
“	CACCACCCAGAGCAG CTTCC	40	4	chr3:47949949	For	0.000496
“	ACACTCTGAGAGCAG CTTTC	40	4	chr1:10530709	For	0.000196
“	GACCT--GAGAGCAGCTTCC	42	3	chr3:67234819	For	0.000888
“	TACCT--GAGAGCAGCTTCC	42	3	chr2:24659636	For	0.000542
“	AGCCT--GAGAGCAGCTTCC	42	3	chr7:129824787	Rev	0.000904
“	AGCCT-GGAAAGCAGCTTCC	43	3	chr18:79407143	Rev	0.000348
“	AACCTCAGGGAGCAA ATTCC	44	4	chr8:8675208	For	0.000870
“	GACTTCGCAGAGCAG CTTCT	44	4	chr2:10424155	Rev	0.000496
“	GATCTCTGAGAGCAG CATCC	45	4	chr2:2692136	Rev	0.000036
“	AACCTCTAAGAGCAA CTTCA	45	4	chr11:13553809	For	0.000790
“	AACCTCG--GAGTAGCTTCC	47	3	chr22:44797637	For	0.000984
“	CACCTCAGAGGACAG CTTCC	47	4	chrX:141895615	For	0.000240
“	AGTCTCGGAGAGAAG CTTCA	47	4	chr1:47654433	Rev	0.000474
“	AACCACTGAGGGAAG CTTCC	48	4	chr1:157136254	For	0.000668
“	AATCTCATAGAGCAGC GTCC	48	4	chr14:50674835	For	0.000902
“	AACCTCACAAAGCAG CTTCA	48	4	chr8:46053277	Rev	0.000106
“	AACCTCACAAAGCAG TTTCC	48	4	chr19:24331210	Rev	0.000958
“	TGCCTCAGAGAGCAG CTGCC	49	4	chr5:90224549	Rev	0.000214
“	CACCTCAGAGGGCAG ATTCC	49	4	chrX:142204984	Rev	0.000558

"	AACCTCAATGGGCAG CTTCC	50	4	chr5:90224585	Rev	0.000370
"	CACCTCTCAGAGCAG CCTCC	50	4	chr10:69945284	Rev	0.033200
"	AGCCT-GGAGAGCAGATTCC	51	3	chr17:7040816	Rev	0.048000
"	ACCTTCGCAGAGCAT CTTCC	51	4	chr19:40221429	For	0.037400
"	AAACTTTCAGAGCAG CTTCC	51	4	chr6:79505763	For	0.086800
"	AACCTTG-ATAGCAGCTTCC	52	3	chr21:35856416	Rev	0.015600
"	AACCACACAGAGCAC CTTCC	52	4	chr7:57387440	Rev	0.024400
"	AAGCACGTAGAGCAG CCTCC	52	4	chr11:64511632	Rev	0.038400
"	AACCTCAAACAGCAG CTTCA	53	4	chr6:147699817	Rev	0.095200
"	AACC-CGGAGCCCAGCTTCC	54	3	chr8:102102588	For	0.001200
"	AATCTCACAGAGCTG CTTCC	54	4	chr18:21958564	Rev	0.046600
"	AGCCTCGGGGAGCAC CTTCC	55	3	chr7:1844582	Rev	0.083200
"	AGCCTCAGGGAGCAG CTGCC	56	4	chr2:88915023	For	0.097200
"	AACCTCTG-CAGCAGCTTCC	57	3	chr14:105316534	For	0.041600
"	AACCACCGAGAGAAG CTTCC	57	3	chr6:28252319	Rev	0.003200
"	CACATCGCAGAGCAG CTTCT	57	4	chr5:48060965	Rev	0.030200
"	CACATCGCAGAGCAG CTTCT	57	4	chr19:25430218	Rev	0.026600
"	CACATCGCAGAGCAG CTTCT	57	4	chr5:47876937	Rev	0.011200
"	CACATCGCAGAGCAG CTTCT	57	4	chr5:48390468	Rev	0.075400
"	CACATCGCAGAGCAG CTTCT	57	4	chr19:25989973	Rev	0.036000
"	AAACTCAGAGAGAAG CTCCC	57	4	chr1:14254330	For	0.006400
"	CACATCGCAGAGCAG CTTCT	57	4	chr1:123024776	Rev	0.069400
"	CACATCGCAGAGCAG CTTCT	57	4	chr1:123255028	Rev	0.007800
"	CACATCGCAGAGCAG CTTCT	57	4	chr1:123071000	Rev	0.004800
"	CACATCGCAGAGCAG CTTCT	57	4	chr19:25660470	Rev	0.077000
"	CACATCGCAGAGCAG CTTCT	57	4	chr5:47830713	Rev	0.022200

"	CACATCGCAGAGCAG CTTCT	57	4	chr1:123584531	Rev	0.085000
"	CACATCGCAGAGCAG CTTCT	57	4	chr19:25476442	Rev	0.044400
"	ACCCTAGGAGAGCAG CCTCC	59	3	chr2:113263029	Rev	0.052200
"	AAGGTCTGAGAGCAG CTTCT	61	4	chr3:128165278	Rev	0.059600
"	ACCCGCGGACAGCAG CCTCC	62	4	chr16:85625375	Rev	0.003400
"	AAACTCTGATAGCAGC TTCT	62	4	chr2:192570702	For	0.016600
"	AACCTC-AAGAGCAGCTTCT	63	3	chr2:4605036	For	0.020000
"	TACCTCTGGGAGCTG CTTCC	63	4	chr20:41748206	For	0.086800
"	AACCTCTGA-AGCAGCATCC	64	3	chr8:26321498	Rev	0.086200
"	ACCCACGGAGAGCAG CCTCA	64	4	chr4:1985991	For	0.010600
"	CACCTCAGAGGGCAG CTTCT	64	4	chr7:30554625	For	0.096200
"	AGCCTCTGGGAGCAG CTTGC	65	4	chr21:30935667	Rev	0.065200
"	GATCTCGGACAGCAG CCTCC	65	4	chr7:3015009	Rev	0.064400
"	AGCCTCTGATAGCAG CTTGC	67	4	chrX:114061362	For	0.004000
"	AGCCTCTGAGAGAAG CCTCC	67	4	chr1:9863419	For	0.069200
"	CACCTAGGAGAGCAC CTTCC	68	3	chr3:194838190	For	0.070000
"	ATCCTCCTAGAGAAGC TTCC	68	4	chr17:51658028	For	0.013600
"	AACCCCTGAGATAAG CTTCC	68	4	chr1:165925830	For	0.041800
"	TACCTGAGAAAGCAG CTTCC	68	4	chr10:23928334	For	0.061400
"	CACCTCGGA-AGCAGCTGCC	69	3	chr20:56159590	For	0.053400
"	CACCTCGCAGAGCAG TTTCT	69	4	chr1:124133054	Rev	0.096200
"	AGCCTTGTAGGGCAG CTTCC	69	4	chr17:1471613	Rev	0.061600
"	CACCTCGCAGAGCAG TTTCT	69	4	chr5:48938991	Rev	0.020600
"	CACCTCGCAGAGCAG TTTCT	69	4	chr19:26538496	Rev	0.012200

[a] Highest off target editing observed.
For, forward.
Rev, reverse.

Supplementary Table 2c. Off-target sites associated with the TrueGuide sgRNA CRISPR283503_SGM, used for *IL30* gene editing in IL30-TRAMP-C1 cells.

Gene	Target DNA Sequence	Score	Mismatch	Position	Strand	Editing (%)
WDTC1	TCGCCTGCCAGCAGTGGACC	49	4	chr4:133300655	Rev	**0.006000**[a]
1810055G02RIK	TCTGTTGTCAGGAGTGTACC	76	4	chr19:3717260	Rev	0.003000
CEP350	TCGATTTCCAGCAGTGAGAC	109	4	chr1:155894644	Rev	0.000010
Non coding	TTGAATACCTGGAGTGAACC	11	4	chr5:66951607	For	0.001200
“	TCAATTAGCAGGAGTAAACC	23	4	chr1:94072570	For	0.000200
“	TAGA-TGCCAGGAGTGAATC	32	3	chr6:8463240	For	0.001200
“	TCCTTTGCCTGAAGTGAACC	34	4	chr3:92574388	Rev	0.001100
“	TTGATTTCCAAGAGTGAACA	36	4	chr13:6936108	Rev	0.000500
“	ACGATAAACAGGAGTGAACC	36	4	chr1:61123441	For	0.000900
“	TTGATTGCCAGGAGAGAACC	37	2	chr2:26074021	For	0.001200
“	TTGTTTGCCAAGTGTGAACC	39	4	chr1:78067042	For	0.000800
“	TCCA-TGCCAGGAGTGAGCC	41	3	chr1:36608936	For	0.000400
“	TCAGTTGCCTGGAGTGAATC	43	4	chr19:10042859	Rev	0.000400
“	GCGATT-CCAGGAATGAACC	44	3	chr1:72839398	For	0.000200
“	TTCATTGCCAGCAGTGAATC	45	4	chr18:38555252	Rev	0.000210
“	TCAATTACCAAGAGTGAGCC	47	4	chr15:6470781	Rev	0.000900
“	CCGATAGCCAGG-GTGAACC	50	3	chr8:11914390	Rev	0.000600
“	ATGATTTCCAGGAGTGAACT	51	4	chr2:58393579	For	0.000100
“	TTGACTGACAGGAGAGAACC	54	4	chr17:64729719	For	0.000900
“	TTGTTTCCCAGCAGTGAACC	54	4	chr6:114916687	For	0.000700
“	TCGTGTGCCAGCAGTGAGCC	54	4	chr3:121610064	Rev	0.000900
“	TCCATTTTCAGGAGTTAACC	57	4	chr7:139828421	Rev	0.000900
“	TCCAGTGCCAGGAGTGGACA	58	4	chrX:157995767	Rev	0.000400

"	TAGAATGCCAGGAGCAAACC	62	4	chr1:151010161	Rev	0.000810
"	TCCATTGCCAGGAGTGAGCA	63	3	chr1:51742465	Rev	0.000900
"	TGGACT-CCAGGAGTGAACC	66	3	chr6:145851237	For	0.000550
"	TGGATTGGCAAGAGTAAACC	66	4	chr11:43833431	For	0.000900
"	TAGGTTCCGAGGAGTGAACC	67	4	chr5:130495151	For	0.000800
"	GAGATTGCCAGGGGTGAGCC	67	4	chr18:4987772	Rev	0.000300
"	TCCATAGCCAGGAGAAAACC	70	4	chr11:88214276	Rev	0.000300
"	ATGATTGCCAGGAGAGGACC	70	4	chr3:131963979	For	0.000890
"	TCTAGTGTTAGGAGTGAACC	71	4	chr9:46528822	Rev	0.000300
"	TAGATTGTCAGGAATGAAAC	72	4	chr4:77020257	Rev	0.000800
"	TAGATTGGAAGGAGAGAACC	72	4	chr6:29115430	Rev	0.000300
"	ACAATTGCTAGGAGTGAACA	74	4	chr16:16992896	Rev	0.000200
"	TTTATTGCCAAGAGTGACCC	74	4	chr11:63181303	For	0.000290
"	TCGGTTGCCATGGATTGAACC	75	3	chr18:4759048	For	0.000600
"	TCAATAGCCAGGAGAAAACC	75	4	chr2:162539588	For	0.000500
"	TTGAATCCCAGGAGAGAACC	75	4	chr4:80518711	For	0.000200
"	ACGATTGTCAGGGGTGAAAC	75	4	chr5:134846550	For	0.000850
"	TTGATTGCCAAGAATGAAGC	77	4	chr1:93179007	For	0.004300
"	TAGAATGCAAGGAGTGAACT	77	4	chr6:86081750	Rev	0.000600
"	TTGATTGCCAGGGTTGAAAC	78	3	chr3:14796487	Rev	0.021000
"	TAGATTGTGAGGATTGAACC	78	4	chr1:83474074	For	0.000900
"	ACTATTGCCAGGAATGAACA	78	4	chr11:28911175	Rev	0.000803
"	TAGGTTCCCAGGAGTGAACT	80	4	chr1:12941024	For	0.000897
"	TCTCTTGCCAGCAGAGAACC	80	4	chr7:117222669	For	0.000660
"	TCAATTTCCAGAAGTGAACT	81	4	chr5:124361298	For	0.000454
"	TCCACTGCCAGGAGAGAAAC	83	4	chr2:174119488	Rev	0.000842

"	TTGATTGACAGGGGA GAACC	83	4	chr3:51326838	For	0.000229
"	TCGATTGCCTAGAATG TACC	86	4	chr3:89939106	For	0.000233
"	TAGATTGCCAGCAGT GACCA	86	4	chr3:121096811	Rev	0.000930
"	TGGATTGTCAGGAGTT GAACC	88	3	chr4:11942974	For	0.000340
"	ACGTTTGCCAGGAGT CAGCC	88	4	chr11:93567974	For	0.000615
"	TCGTTTGCCAGGA-TGAAAC	89	3	chr3:66685492	Rev	0.000213
"	TCGA-TGCCAGGAGTCATCC	94	3	chr6:138495920	For	0.000266
"	TCAATTGCCAGGA-TGAAAC	94	3	chr3:38781873	Rev	0.000298
"	TAGGTTGCCATGAGTC AACC	94	4	chr14:98077623	Rev	0.000414
"	TTGATTGCTAGAAATG AACC	95	4	chr13:22826814	Rev	0.000657
"	TTGATTGACAGGAGA GATCC	95	4	chr1:193843913	Rev	0.000533
"	TTGTTTGCCAGGAGT GAAGG	96	4	chr12:56120669	For	0.000465
"	TAGATTCCCAGCAGTG AACT	96	4	chr7:46778346	For	0.000802
"	TCGATTGCCAAGAATG ATTC	98	4	chr19:43439241	Rev	0.000150
"	TCAATTTCCAGGAGTT AGCC	100	4	chr6:54916337	For	0.000448
"	TCAGTTGCCAGGATT GAAAC	100	4	chrX:11545442	Rev	0.000222
"	TCTAT-GCCAGGAGTCAACC	101	3	chr9:69136923	Rev	0.001631
"	TGGATTGCCAGCAGT GAGCC	102	3	chr15:95801826	For	0.000960
"	TAGATGGCCAGGAGT GAATA	103	4	chr6:91203630	For	0.000648
"	TGGATTTGCAGGAGT GAAAC	103	4	chrX:63088035	Rev	0.000347
"	TAGATGGCCAGGAGT GAATA	103	4	chr6:91203718	For	0.000630
"	TCCATTGACAGGATAG AACC	103	4	chr7:38252248	Rev	0.000255
"	TTGATTGACAGGTGTG AAGC	103	4	chr19:58044442	For	0.000356
"	TCAATTGCCAGGGGA GGACC	104	4	chr5:144518114	For	0.001066
"	TCCATTGCCAGAAGT GGACG	105	4	chr14:68377036	For	0.000564
"	TAGATTGCCAGCAGG GACCC	105	4	chr19:45201584	For	0.000761

"	TCTTTTGCAAGGAGTGACCC	107	4	chr16:18425907	For	0.000752
"	TCAATTTCTAGGAGTCAACC	108	4	chr5:4472763	Rev	0.000819
"	TCGATTGGGATGAGAGAACC	110	4	chr11:63692037	For	0.003700
"	TTGATTGACAGGACTGAAGC	112	4	chr2:127511664	Rev	0.005600
"	TGGATTCCCAAAAGTGAACC	113	4	chrX:106510822	For	0.000500
"	TGGGTTGACAGGAGTGTACC	114	4	chr5:35153970	For	0.000606
"	TCTAATCCCAGGATTGAACC	114	4	chr17:60127461	For	0.000260
"	TCGAT-GCCAGGATTGAACT	115	3	chr14:57327360	Rev	0.000832
"	TGGATTGCCAGGA-TGAACA	116	3	chr3:18280038	For	0.000412
"	TGGAT-GCTAGGAGTGAACC	116	3	chr2:142086337	Rev	0.000237
"	GCGATTGCCAGGAGACAATC	116	4	chr7:80851640	Rev	0.002460
"	TCAATGGCCAGGAAAGAACC	117	4	chr13:35107969	Rev	0.003500
"	TCTATTGGCAGGAGTGATGC	117	4	chr1:156923715	For	0.002840
"	TCCATTGCTAGGAGTGACTC	118	4	chr4:63279384	For	0.002300
"	TGGATTGCCTGGAGAGAACA	119	4	chr15:78791224	For	0.003900
"	TCGAGTGCCAGGAGAGCTCC	120	4	chr1:54557341	For	0.002109
"	TGGGTTGCCAGGTGTGAGCC	123	4	chr8:119897638	Rev	0.001900
"	TCCATGGCCAGAATTGAACC	123	4	chrY:242918	For	0.004630
"	TTGATGGCCAGGAATTAACC	123	4	chr1:134020393	For	0.001961
"	TCCATGGCCAGAATTGAACC	123	4	chrY:286486	Rev	0.005200
"	TCGAGTGCCAGGTGGGAACT	123	4	chr11:57717108	Rev	0.002500
"	TCCATGGCCAGAATTGAACC	123	4	chrY:117755	For	0.004400
"	TTGATTGCTAGGAGAGCACC	123	4	chrX:164705743	For	0.003400
"	TCCATGGCCAGAATTGAACC	123	4	chrY:161678	Rev	0.005000

[a] Highest off target editing observed.
For, forward.
Rev, reverse.

Supplementary Table 3. Antibodies used in immunostaining.

Antibody	Clone	Origin	Research Resource Identifiers (RRIDs) or product code	Source
Anti human				
ANG		Goat	RRID:AB_2227144	R& D Systems, Minneapolis, MN, USA
CDH1	NCH-38	Mouse	RRID:AB_2076672	Agilent, Santa Clara, CA, USA
CXCL8		Rabbit	RRID:AB_2536263	Thermo Fisher, Waltham, MA, USA
DKK3		Rabbit	RRID:AB_2852023	Thermo Fisher, Waltham, MA, USA
EGF	10825	Mouse	RRID:AB_2095960	R&D Systems, Minneapolis, MN, USA
HGF		Rabbit	RRID:AB_2676939	Atlas Antibodies, Stockholm, SE
IGF1		Rabbit	RRID:AB_308724	Abcam, Cambridge, UK
IL30		Rabbit	RRID:AB_10898806	"
Ki67	MIB1	Mouse	RRID:AB_2142367	Agilent, Santa Clara, CA, USA
NFKB1*	E381	Rabbit	RRID:AB_776748	Abcam, Cambridge, UK
PSCA		Rabbit	RRID:AB_2662130	Thermo Fisher, Waltham, MA, USA
PTEN	28H6	Mouse	RRID:AB_383709	GeneTex, Hsinchu City, Taiwan
PTGS2		Rabbit	RRID:AB_1951531	"
TGFβ1		Rabbit	RRID:AB_632486	Santa Cruz Biotechnology, Dallas, TX, USA
Anti mouse				
CD11b	EPR1344	Rabbit	RRID:AB_2650514	Abcam, Cambridge, UK
CD31	SZ31	Rat	RRID:AB_2631039	Dianova, Hamburg, Germany
EpCAM	ARC0277	Rabbit	RRID:AB_2849185	Thermo Fisher, Waltham, MA, USA
F4/80	Cl:A3-1	Rat	RRID:AB_323279	Bio-Rad, Hercules, CA, USA
Foxp3	FJK-16s	Rat	RRID:AB_467576	Thermo Fisher, Waltham, MA, USA
Gr-1	RB6-8C5	Rat	RRID:AB_394638	BD Biosciences, Franklin Lakes, NJ, USA
IL30		Goat	RRID: AB_355012	R&D Systems, Minneapolis, MN, USA
Ly-6G	1A8	Rat	RRID:AB_1089179	BioLegend, San Diego, CA, USA
NKp46		Rabbit	RRID:AB_10767953	Biorbyt, Cambridge, UK
PCNA	PC10	Mouse	RRID:AB_2160651	Agilent, Santa Clara, CA, USA
PSCA		Human	HPAB-1900-FY-S(P)	Creative Biolabs, Shirley, NY, USA
RORγt	AFKJS-9	Rat	RRID:AB_1834475	Thermo Fisher, Waltham, MA, USA

*Antibodies used for immunohistochemistry on both human and murine tissues.

Supplementary Table 4. Particle size and zeta potential of Empty-hPSCA and Cas9hIL30-hPSCA NxPs.

NxPs	Size (nm)	Zeta (mV)
**Empty-hPSCA NxPs**	70.2 ± 0.5	29.17 ± 3.56
**Cas9gRNAhIL30-PSCA NxPs**	85.4 ± 0.7	3.42 ± 1.49

Supplementary Table 5. Average number of lung metastases in tumor-bearing NSG mice treated with PBS or NxPs coniugated or not with anti-hPSCA Abs.

Tumor	Treatment		
	PBS	Empty-hPSCA NxPs	Cas9hIL30-hPSCA NxPs
**PC3***	22 ± 9	22 ± 8	28 ± 8
**IL30KO-PC3***	18 ± 12[#]	////	////
**DU145***	5 ± 2	5 ± 2	8 ± 5[†]
**IL30KO-DU145***	4 ± 1[†]	////	////

*****No significant difference was disclosed, by ANOVA (p>0.05), in the average number of lung metastases, between mice implanted with wild type PC3 or DU145 cells, and treated with Ab coniugates NxPs or PBS, and mice implanted with IL30KO-PC3 or IL30KO-DU145 cells and treated with PBS.

[#]In this group the number refers to micrometastases (≤500 µm).

[†]In these groups the number of metastases refers to 40% (DU145 + Cas9hIL30-hPSCA NxPs) or 50% (IL30KO-DU145) of the mice which developed metastases.

Supplementary Table 6. Immunohistochemical analysis of viability and vascularity of tumors from mice treated with Cas9hIL30-hPSCA NxPs, Empty-hPSCA NxPs or PBS.

	PC3			IL30KO-PC3	DU145[b]			ANOVA *p* value[c]
	**PBS**	**Empty-hPSCA NxPs**	**Cas9hIL30-hPSCA NxPs**	**PBS**	**PBS**	**Empty-hPSCA NxPs**	**Cas9hIL30-hPSCA NxPs**	
**Microvessel density[a]**	12.08 ± 2.73	11.10 ± 2.85	3.73[c] ± 2.60	4.57[d] ± 1.95	12.92 ± 3.65	13.27 ± 3.48	4.25[e] ± 2.70	<0.0001
**Proliferation index (%)[a]**	64.13 ± 7.65	62.35 ± 8.13	24.60[c] ± 7.05	19.28[d] ± 5.33	52.60 ± 8.40	51.97 ± 7.64	20.75[e] ± 6.82	<0.0001
**Apoptotic index (%)[a]**	5.90 ± 3.73	6.45 ± 2.70	7.95 ± 3.32	8.02 ± 3.10	4.62 ± 3.57	6.62 ± 3.45	6.83 ± 4.23	<0.0001

[a] Microvessel density, Proliferation and Apoptotic index were assessed by light microscopy, at ×400 in an 85431.59 μm² field, with Qwin image analysis software (version 2.7). Results are expressed as mean ± SD of CD31 positive microvessels per field (microvessel density), or mean percentage of Ki67 (proliferation) or TUNEL (apoptosis) positive cells/number of total cells.
[b] Results obtained from mice bearing IL30KO-DU145 tumors are reported in ref. 14.
[c] One-way ANOVA for comparisons between all groups.
[d] $p<0.01$ Tukey's HSD test compared with PC3 cells treated with PBS or Empty-hPSCA NxPs.
[e] $p<0.01$ Tukey's HSD test compared with DU145 cells treated with PBS or Empty-hPSCA NxPs.

Supplementary Table 7. Immunohistochemical analysis of angiogenic and prostate cancer driver gene expression in IL30KO-PC3 tumors treated with PBS and in wild type PC3 tumors treated with PBS, Empty-hPSCA NxPs or Cas9hIL30-hPSCA NxPs.

**Angiogenic Factors and PC driver genes[a]**	**WT-PC3**	**PC3 + Cas9hIL30-hPSCA NxPs**	**PC3 + Empty-hPSCA NxPs**	**IL30KO-PC3**
**IL30**	9.0 ± 1.5	1.6 ± 0.8[b]	9.3 ± 1.9	0.7 ± 0.3
**IGF1**	15.8 ± 4.2	3.4 ± 1.2[b]	15.0 ± 3.8	3.8 ± 1.4[b]
**CXCL8**	11.0 ± 3.6	1.6 ± 0.9[b]	12.9 ± 3.5	1.9 ± 1.2[b]
**TGFβ1**	19.4 ± 6.2	6.2 ± 1.5[b]	17.8 ± 5.5	4.7 ± 1.2[b]
**ANG**	12.3 ± 2.0	4.7 ± 1.5[b]	11.5 ± 2.3	3.0 ± 1.2[b]
**HGF**	17.3 ± 3.8	3.2 ± 1.9[b]	18.2 ± 4.2	3.6 ± 1.5[b]
**EGF**	16.0 ± 3.5	5.6 ± 1.1[b]	15.5 ± 3.0	6.4 ± 1.7[b]
**PTGS2**	18.8 ± 3.8	7.3 ± 1.7[b]	17.9 ± 3.7	6.9 ± 2.5[b]
**NFKB1**	16.2 ± 4.1	8.0 ± 2.6[b]	15.6 ± 3.9	7.5 ± 2.2[b]
**DKK3**	3.7 ± 1.4	18.2 ± 4.2[b]	3.4 ± 1.6	19.8 ± 4.0[b]

[a]Automated immunostaining quantification of angiogenic and cancer driver gene expression was performed as described in Supplementary Materials and Methods. Expression values of angiogenic and cancer driver genes are represented as the mean percentage ± SD of positively stained areas (evaluated by assessment of both the widening and the strength of the staining)/total area of the examined field (85431.59 μm^2) at ×400.

[b]Values significantly different (Student's *t*-test: $p<0.01$) from the corresponding values in WT-PC3 tumors and in PC3 tumors + Empty-hPSCA NxPs.

Supplementary Table 8. Immunohistochemical analysis of angiogenic and prostate cancer driver gene expression in wild type DU145 tumors treated with PBS, Empty-hPSCA NxPs or Cas9hIL30-hPSCA NxPs.

Angiogenic Factors and PC driver genes[a]	WT-DU145	DU145 + Cas9hIL30-hPSCA NxPs	DU145 + Empty-hPSCA NxPs
**IL30**	11.8 ± 2.7	1.8± 0.7[b]	12.3 ± 3.0
**IGF1**	14.5 ± 4.3	2.8 ± 1.4[b]	13.6 ± 3.3
**CXCL8**	13.2 ± 3.5	1.9 ± 0.5[b]	12.7 ± 2.8
**PTGS2**	17.0 ± 3.3	4.1 ± 1.8[b]	16.7 ± 4.0
**NFKB1**	14.2 ± 3.6	5.0 ± 2.6[b]	15.2 ± 3.4
**DKK3**	4.9 ± 1.3	14.7 ± 3.3[b]	5.2 ± 1.5
**CDH1**	5.6±1.7	10.4±2.5[b]	5.0±1.4
**PTEN**	16.2±3.2	4.5±1.7[b]	15.6±4.0

[a]Automated immunostaining quantification of angiogenic and prostate cancer driver gene expression was performed as described in Supplementary Materials and Methods. Expression values of cancer driver genes are represented as the mean percentage ± SD of positively stained areas (evaluated by assessment of both the widening and the strength of the staining)/total area of the examined field (85431.59 μm^2) at ×400.

[b]Values significantly different (Student's *t*-test: $p<0.01$) from the corresponding values in WT-DU145 tumors and in DU145 tumors + Empty-hPSCA NxPs.

Supplementary Table 9. Levels of metabolic markers and inflammatory cytokines in serum samples from BALB/c mice treated with Cas9hIL30-hPSCA NxPs or PBS.

	**BALB/c**	
	**PBS**	**Cas9hIL30-hPSCA NxPs**†
Metabolic markers*		
ALT	16.71 ± 2.66 IU/l	17.41 ± 2.78 IU/l
AST	26.12 ± 2.50 IU/l	27.44 ± 3.80 IU/l
BUN	22.34 ± 3.10 mg/dl	25.34 ± 4.50 mg/dl
CK	158.91 ± 10.33 u/l	162.91 ± 11.21 u/l
Cr	0.57 ± 0.06 mg/dl	0.67 ± 0.16 mg/dl
cTn1	35.30 ± 3.85 ng/ml	38.30 ± 5.91 ng/ml
LDH	280.32 ± 14.77 u/ml	290.32 ± 18.05 u/ml
Cytokines#		
IL6	100.22 ± 18.11 pg/ml	110.45 ± 19.76 pg/ml
TNFα	55.08 ± 20.34 pg/ml	59.10 ± 15.44 pg/ml

ALT, alanine aminotransferase; **AST**, aspartate aminotransferase; **BUN**, blood urea nitrogen; **CK**, creatine kinase; **Cr**, creatinine; **cTn1**, cardiac troponin-1; **IU/l**, international units per liter; **LDH**, lactate dehydrogenase; **u/l**, units per liter; **u/ml**, units per milliliter.
*****Levels measured 21 days after starting treatment.
#Levels measured 24h after the first treatment.
†Results from mice treated with naked Cas9hIL30 complex or Empty-hPSCA NxPs were comparable to those from mice treated with Cas9hIL30-hPSCA NxPs.

Supplementary Table 10. Body weight changes in BALB/c mice treated with PBS, naked CRISPR/Cas9gRNA-hIL30 complex (Cas9hIL30), unconjugated NxPs (Cas9hIL30 NxPs), Empty-hPSCA NxPs and Cas9hIL30-hPSCA NxPs.

**Mouse number**	**Group 1*** *PBS*	**Group 2*** *Cas9hIL30*	**Group 3*** *Cas9hIL30 NxPs*	**Group 4*** *Empty-hPSCA NxPs*	**Group 5*** *Cas9hIL30-hPSCA NxPs*
	Before starting treatment				
1	26	25	27	26	26
2	25	26	25	26	27
3	27	26	26	27	26
4	27	27	27	26	25
5	27	26	26	25	26
Mean	*26.40*	*26.00*	*26.20*	*26.00*	*26.00*
SD	*0.89*	*0.71*	*0.84*	*0.71*	*0.71*
	30 days after starting treatment				
1	26	25	27	26	26
2	25	26	25	26	27
3	28	28	26	27	28
4	27	27	27	26	25
5	27	26	26	25	26
Mean	*26.60*	*26.40*	*26.20*	*26.00*	*26.40*
SD	*1.14*	*1.14*	*0.84*	*0.71*	*1.14*
	60 days after starting treatment				
1	27	26	27	26	27
2	26	27	26	26	27
3	28	28	27	28	28
4	27	27	25	28	26
5	28	26	25	26	27
Mean	*27.20*	*26.80*	*26.00*	*26.80*	*27.00*
SD	*0.84*	*0.84*	*1.00*	*1.10*	*0.71*
	74 days after starting treatment				
1	27	27	29	27	27
2	27	27	27	27	27
3	29	28	28	29	28
4	27	27	26	28	27
5	28	27	26	26	28
Mean	*27.60*	*27.20*	*27.20*	*27.40*	*27.40*
SD	*0.89*	*0.45*	*1.30*	*1.14*	*0.55*

*The body weight is expressed in grams.

Supplementary Table 11. Particle size and zeta potential of Empty-mPSCA and Cas9mIL30-mPSCA NxPs.

NxPs	Size (nm)	Zeta (mV)
**Empty-mPSCA NxPs**	69.5 ± 0.7	30.23 ± 4.77
**Cas9gRNAmIL30-mPSCA NxPs**	86.2 ± 0.6	4.27 ± 1.82

Supplementary Table 12. Levels of metabolic markers and inflammatory cytokines in serum samples from C57BL/6J mice treated with Cas9mIL30-mPSCA NxPs or PBS.

	**C57BL/6J**	
	**PBS**	**Cas9mIL30-mPSCA NxPs†**
Metabolic markers*		
ALT	14.96 ± 1.82 IU/l	15.00 ± 2.90 IU/l
AST	28.55 ± 3.92 IU/l	30.10 ± 4.00 IU/l
BUN	19.12 ± 2.72 mg/dl	22.23 ± 3.95 mg/dl
CK	165.34 ± 17.42 u/l	170.91 ± 15.92 u/l
Cr	0.62 ± 0.18 mg/dl	0.69 ± 0.08 mg/dl
cTn1	37.88 ± 4.29 ng/ml	41.44 ± 6.58 ng/ml
LDH	288.18 ± 10.80 u/ml	295.28 ± 12.30 u/ml
Cytokines#		
IL6	105.59 ± 15.98 pg/ml	112.79 ± 18.86 pg/ml
TNFα	57.08 ± 18.43 pg/ml	62.11 ± 20.50 pg/ml

ALT, alanine aminotransferase; **AST**, aspartate aminotransferase; **BUN**, blood urea nitrogen; **CK**, creatine kinase; **Cr**, creatinine; **cTn1**, cardiac troponin-1; **IU/l**, international units per liter; **LDH**, lactate dehydrogenase; **u/l**, units per liter; **u/ml**, units per milliliter.

*****Levels measured 21 days after starting treatment.

#Levels measured 24h after the first treatment.

†Results from mice treated with naked Cas9mIL30 complex or Empty-mPSCA NxPs were comparable to those from mice treated with Cas9mIL30-mPSCA NxPs.

Supplementary Table 13. Immunohistochemical analysis of proliferation and vascularization in IL30-TRAMP-C1 tumors from mice treated with Cas9mIL30-mPSCA NxPs, Empty-mPSCA NxPs or PBS, and in wild type TRAMP-C1 tumors from mice treated with PBS.

	IL30-TRAMP-C1			TRAMP-C1	ANOVA *p* value[b]
	**PBS**	**Empty-mPSCA NxPs**	**Cas9mIL30-mPSCA NxPs**	**PBS**	
Microvessel density[a]	21.64 ± 3.75	22.36 ± 4.25	9.15[c] ± 3.20	13.57[c] ± 2.85	<0.0001
Proliferation index (%)[a]	61.53 ± 8.95	63.15 ± 7.75	23.12[c] ± 6.04	29.66[c] ± 5.43	<0.0001

[a] Microvessel density and proliferation index were assessed by light microscopy, at ×400 in an 85431.59 μm^2 field, with Qwin image analysis software (version 2.7). Results are expressed as mean ± SD of CD31 positive microvessels per field (microvessel density), or mean percentage of PCNA positive cells/number of total cells (proliferation index).

[b] One-way ANOVA for comparisons between all groups.

[c] p<0.01 Tukey's HSD test compared with IL30-TRAMP-C1 cells treated with PBS or Empty-mPSCA NxPs.
